# Association Between the Digital Clock Drawing Test and Neuropsychological Test Performance: Large Community-Based Prospective Cohort (Framingham Heart Study)

**DOI:** 10.2196/27407

**Published:** 2021-06-08

**Authors:** Jing Yuan, David J Libon, Cody Karjadi, Alvin F A Ang, Sherral Devine, Sanford H Auerbach, Rhoda Au, Honghuang Lin

**Affiliations:** 1 Department of Neurology Peking Union Medical College Hospital Chinese Academy of Medical Sciences Beijing China; 2 Department of Anatomy and Neurobiology School of Medicine Boston University Boston, MA United States; 3 Department of Geriatrics and Gerontology and the Department of Psychology New Jersey Institute for Successful Aging School of Osteopathic Medicine, Rowan University Stratford, NJ United States; 4 Framingham Heart Study School of Medicine Boston University Boston, MA United States; 5 Slone Epidemiology Center School of Medicine Boston University Boston, MA United States; 6 Department of Neurology School of Medicine Boston University Boston, MA United States; 7 Department of Epidemiology School of Public Health Boston University Boston, MA United States; 8 Computational Biomedicine School of Medicine Boston University Boston, MA United States

**Keywords:** clock drawing test, neuropsychological test, cognition, technology, digital assessment, mild cognitive impairment, association, neurology, Framingham Heart Study

## Abstract

**Background:**

The Clock Drawing Test (CDT) has been widely used in clinic for cognitive assessment. Recently, a digital Clock Drawing Text (dCDT) that is able to capture the entire sequence of clock drawing behaviors was introduced. While a variety of domain-specific features can be derived from the dCDT, it has not yet been evaluated in a large community-based population whether the features derived from the dCDT correlate with cognitive function.

**Objective:**

We aimed to investigate the association between dCDT features and cognitive performance across multiple domains.

**Methods:**

Participants from the Framingham Heart Study, a large community-based cohort with longitudinal cognitive surveillance, who did not have dementia were included. Participants were administered both the dCDT and a standard protocol of neuropsychological tests that measured a wide range of cognitive functions. A total of 105 features were derived from the dCDT, and their associations with 18 neuropsychological tests were assessed with linear regression models adjusted for age and sex. Associations between a composite score from dCDT features were also assessed for associations with each neuropsychological test and cognitive status (clinically diagnosed mild cognitive impairment compared to normal cognition).

**Results:**

The study included 2062 participants (age: mean 62, SD 13 years, 51.6% women), among whom 36 were diagnosed with mild cognitive impairment. Each neuropsychological test was associated with an average of 50 dCDT features. The composite scores derived from dCDT features were significantly associated with both neuropsychological tests and mild cognitive impairment.

**Conclusions:**

The dCDT can potentially be used as a tool for cognitive assessment in large community-based populations.

## Introduction

The Clock Drawing Test (CDT) is a widely used neuropsychological test to screen cognitive impairment and dementia because of its ease of administration and clinical assessment capability [1,2]. The test is typically administered by specifying a time, for example, ten past eleven, and asking patients or participants to draw a clock showing that time (the command condition), followed by asking patients or participants to copy a predrawn clock image (the copy condition). Both test conditions require multiple cognitive domains. The command test condition requires intact attention, auditory comprehension, semantic memory, executive function, and visuoconstructional abilities, whereas the copy test condition relies primarily upon visuospatial, attention, and executive function skills [3-6]. Keen observation of the process by which drawings are produced is key to the evaluation of the type and severity of cognitive impairment [7,8]. Multiple manual scoring systems have been created to objectively quantify test performance. However, none of these scoring systems can capture the full breadth of cognitive skills used in completing the test [3].

Recently, a digital version of the CDT (dCDT) that uses a digital ballpoint pen and smart paper was developed as an alternative to the standard clock drawing scoring systems [9,10]. The digital pen can record its position with a timestamp and has excellent precision in capturing all graphomotor, spatial, and temporal information [11-13]; however, the characterization of these features and their correlations with standard neuropsychological tests has yet to be examined in a large community-based setting.

The objective of this investigation was to examine the association between dCDT features and cognitive functions in the Framingham Heart Study (FHS) cohort. We also investigated the association between dCDT features and cognitive status (clinically diagnosed mild cognitive impairment compared to those with normal cognition).

## Methods

### Study Sample

The FHS is a community-based prospective cohort study that was established in 1948. Three generations of participants have been enrolled. Details about the FHS cohort have been previously published [14-16]. This study included participants who completed at least one dCDT and neuropsychological assessment. Participants with prevalent dementia (n=23) or who had not been reviewed by the expert panel (n=138) were excluded. The Boston University Medical Campus Institutional Review Board approved the study procedures and protocols. Written informed consent was obtained from all participants.

### The dCDT

**Figure 1:**
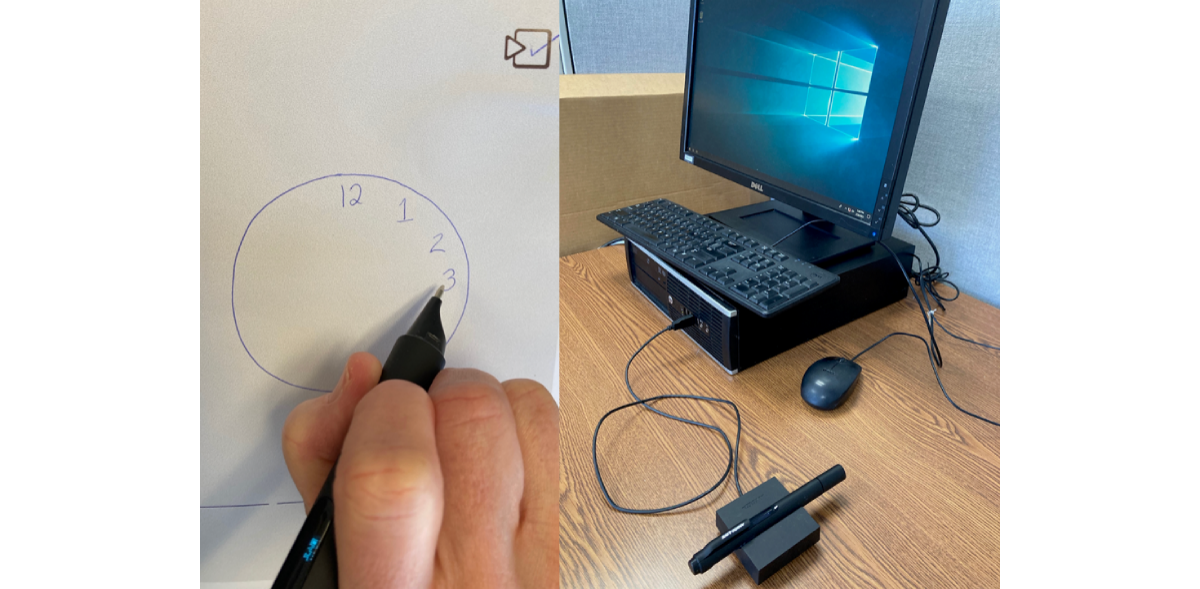
Digital Clock Drawing Test digital pen, smart paper, and docking setup.

Since October 2011, FHS participants who have come for their regular neuropsychological test visit were simultaneously administered the dCDT using a digital pen. The test was jointly developed by the Massachusetts Institute of Technology and the Lahey Hospital and Medical Center with the collaboration of the Clock Sketch Consortium [9-11,17]. Participants used the digital pen (Anoto Inc), to draw a clock on smart paper with a faint dot pattern (Figure 1). The digital pen functions as a regular ballpoint pen does but also measures the pen’s position 80 times per second at a spatial resolution of 0.002 inches with a built-in camera [10,11]. Drawings are automatically classified into different categories, such as numbers, hands, and lines. For quality control, an external rater can replay and deconstruct each drawing to ensure appropriate classification. It typically takes 1 to 2 minutes to classify clocks drawn by healthy or mildly impaired individuals. Any classification errors can be corrected by the rater using a user-friendly drag-and-drop interface. Additional time may be required for classification of more complex clock images, however, most tasks are completed within 5 minutes.

More than 100 dCDT features have been derived to measure the entire drawing process, including capturing all strokes and their corresponding latencies. These features reflect a range of cognitive functions related to drawing efficiency, simple motor operations, information processing speed, and spatial reasoning (Multimedia Appendix 1, Table S1). The rank-based inverse normal transformation was later applied to all dCDT features to reduce the distribution skewness.

### Neuropsychological Assessment

A neuropsychological test protocol, measuring multiple cognitive domains of verbal memory, visual memory, attention and concentration, executive function, abstract reasoning, language, and visuoperceptual organization, was administered to all FHS participants [18]: Wechsler Memory Scale [19] Logical Memory—Immediate Recall, Delayed Recall, and Recognition; Visual Reproduction—Immediate Recall, Delayed Recall, and Recognition; Paired Associate Learning—Immediate Recall, Delayed Recall, and Recognition; the Wechsler Adult Intelligence Scale [20] Digit Span—Forward, Backward, and Similarities; Boston Naming Test—30-item version [21]; Trail Making Test A and B [22]; Hooper Visual Organization Test [23]; Verbal Fluency and Verbal Fluency—Animal [24,25]. All tests were administered by trained raters.

### Ascertainment of Mild Cognitive Impairment

In addition to regular research center visits, FHS participants underwent neuropsychological assessments every 4 to 5 years [26,27]. For participants with possible cognitive impairment, regular neuropsychological tests were conducted every 1 to 2 years and neurological exams were performed on a subset of participants. When potential cognitive impairment decline was present, a clinical review was conducted by a panel with at least one neurologist and one neuropsychologist. Mild cognitive impairment diagnosis was determined by the review panel, which required that the patient exhibit evidence of a decline in cognitive performance in one or more cognitive domains, have no records indicating functional decline, and did not meet criteria for dementia. Although the Clinical Dementia Rating scale [28] was not formally applied, the panel used the Clinical Dementia Rating scoring scale (0-3) to quantify the severity of impairment; for mild cognitive impairment, a rating of 0.5 was given.

### Statistical Analyses

Linear regression models were used to assess the association between each dCDT feature and neuropsychological tests. The models were adjusted for age and sex. Bonferroni correction was used to adjust for multiple testing, and significant associations were claimed if *P*<.05/*n*_t_, where *n*_t_ was the number of tests performed; therefore, tests were significant if *P*<4.8x10^–4^, with α=.05 and 105 tests. A composite score was also created for each neuropsychological test based on dCDT features that were significantly associated with the test. The score for sample *i* is defined as


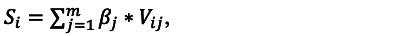


where *m* is the number of dCDT features significantly associated with the neuropsychological test, β_j_ is the estimate of effect size for feature *j*, and *V*_ij_ is the normalized dCDT feature *j* for sample *i*. The score represented a weighted combination of all dCDT features for the neuropsychological test. The associations between the composite score and each neuropsychological test were also tested with linear regression models adjusted for age and sex.

The association between neuropsychological tests and dCDT composite scores with mild cognitive impairment was assessed by logistic regression models adjusted for age, sex, and education. Age was treated as a continuous variable, whereas sex was treated as a dichotomous variable. Education was treated as a categorical variable (eg, no high school degree, high school degree, some college, and college graduate). The difference between groups was assessed with the Wilcoxon rank-sum test for continuous variables or the chi-square test for dichotomous and categorical variables. Bonferroni correction was used to adjust for multiple tests, and associations were significant if *P*< 2.8×10^–3^, given that 18 neuropsychological tests were used. All statistical analyses were performed using R software (version 4.0.3, The R Project).

## Results

As shown in Table 1, our study sample included 2,062 participants (age: mean 62, SD 13 years; 51.6% women; and 43.4% received college-level education or higher). Among them, 36 participants had been diagnosed with mild cognitive impairment. As expected, participants with mild cognitive impairment were generally older and had worse cognitive performance than those in the normal cognition group.

**Table 1:** Clinical characterization.

Variable			All (N=2062)		Mild cognitive impairment (n=36)		Normal cognition (n=2026)		*P* value
Age (years), mean (SD)			62 (13)		79 (7)		62 (13)		<.001
**Gender, n (%)**									.50
	Women	1065 (51.6)		21 (58.3)		1044 (51.5)			
	Men	997 (48.4)		15 (41.7)		982 (48.5)			
**Education, n (%)**									>.99
	No high school	229 (11.1)		4 (11.1)		225 (11.1)			
	High school	386 (18.7)		7 (19.4)		379 (18.7)			
	Some college	551 (26.7)		9 (25.0)		542 (26.8)			
	College and higher	896 (43.4)		16 (44.4)		880 (43.4)			
**Neuropsychological test score, mean (SD)**									
	Logical Memory—Immediate Recall	12 (3)		10 (3)		13 (3)		<.001	
	Logical Memory—Delayed Recall	12 (4)		8 (3)		12 (4)		<.001	
	Logical Memory—Recognition	10 (1)		9 (2)		10 (1)		.002	
	Visual Reproduction—Immediate Recall	8 (3)		5 (2)		8 (3)		<.001	
	Visual Reproduction—Delayed Recall	8 (3)		3 (2)		8 (3)		<.001	
	Visual Reproduction—Recognition	3 (1)		2 (1)		3 (1)		<.001	
	Paired Associate Learning—Immediate Recall	15 (4)		11 (3)		15 (3)		<.001	
	Paired Associate Learning—Delayed Recall	9 (1)		7 (2)		9 (1)		<.001	
	Paired Associate Learning—Recognition	10 (2)		9 (2)		10 (2)		<.001	
	Digit Span—Forward	7 (1)		6 (1)		7 (1)		<.001	
	Digit Span—Backward	5 (1)		4 (1)		5 (1)		<.001	
	Similarities	17 (3)		14 (4)		17 (3)		<.001	
	Boston Naming Test—30-item version	26 (7)		23 (6)		26 (7)		<.001	
	Trail Making Test A (seconds)	32 (17)		47 (13)		32 (17)		<.001	
	Trail Making Test B (seconds)	88 (73)		213 (131)		86 (70)		<.001	
	Hooper Visual Organization Test	25 (3)		22 (3)		26 (3)		<.001	
	Verbal Fluency	41 (12)		31 (13)		41 (12)		<.001	
	Verbal Fluency—Animal	19 (6)		13 (5)		19 (6)		<.001	

A total of 105 distinct dCDT features were derived from each dCDT drawing. Associations between each individual dCDT feature and 18 neuropsychological tests assessing different cognitive functions are shown in Table S1 (Multimedia Appendix 1). In addition, dCDT features that were significantly associated different cognitive domains were summarized in Table S2 (Multimedia Appendix 1). On average, each neuropsychological test was associated with 50 dCDT features after adjusting for multiple testing.

The weighted composite scores built from significant dCDT features for each neuropsychological test were all significantly associated with their corresponding neuropsychological tests (Table 2).

**Table 2:** Association between digital Clock Drawing Test (dCDT) composite scores and neuropsychological tests.

Neuropsychological test	Significant dCDT features, n	Participants, n	Effect size	Standard error	Bonferroni-corrected *P* value^a^
Logical Memory—Immediate Recall	48	2048	0.0625	0.0061	7.4×10^-24^
Logical Memory—Delayed Recall	54	2047	0.0571	0.0059	8.1×10^-22^
Logical Memory—Recognition	28	2037	0.0681	0.0086	4.2×10^-15^
Visual Reproduction—Immediate Recall	62	2049	0.0592	0.0041	3.0×10^-45^
Visual Reproduction—Delayed Recall	61	2048	0.0596	0.0042	8.0×10^-44^
Visual Reproduction—Recognition	69	2043	0.0548	0.0044	5.3×10^-35^
Paired Associate Learning—Immediate Recall	32	1991	0.0966	0.0104	5.8×10^-20^
Paired Associate Learning—Delayed Recall	50	2026	0.0612	0.0064	5.2×10^-21^
Paired Associate Learning—Recognition	13	2062	0.1561	0.0287	5.8×10^-8^
Digit Span—Forward	32	2053	0.0773	0.0092	8.4×10^-17^
Digit Span—Backward	35	2032	0.0780	0.0090	8.0×10^-18^
Trail Making Test A	81	2043	0.0404	0.0019	6.5×10^-87^
Trail Making Test B	86	1999	0.0428	0.0026	1.0×10^-56^
Similarities	46	2034	0.0783	0.0072	4.1×10^-27^
Hooper Visual Organization Test	66	2007	0.0583	0.0041	2.5×10^-44^
Boston Naming Test—30-item version	47	2062	0.0615	0.0063	2.7×10^-22^
Verbal Fluency	66	2007	0.0523	0.0048	4.0×10^-27^
Verbal Fluency—Animal	31	2062	0.0637	0.0068	1.2×10^-20^

^a^All *P* values remained significant after Bonferroni correction (*P*<2.8×10^–3^).

Eight neuropsychological tests were significantly associated with mild cognitive impairment (*P*<2.8×10^–3^), including Visual Reproduction—Delayed Recall, Visual Reproduction—Immediate Recall, Visual Reproduction—Recognition, Paired Associate Learning—Immediate Recall, Paired Associate Learning—Delayed Recall, Digit Span—Backward, Trail Making Test B, and Logical Memory—Delayed Recall (Table 3). All dCDT composite scores were significantly associated with mild cognitive impairment (*P*<2.8x10^–3^).

**Table 3:** Association between neuropsychological tests and digital Clock Drawing Test (dCDT) composite scores and mild cognitive impairment.

Test type	Neuropsychological tests				dCDT composite scores		
	Coefficient estimate	Standard error	*P* value^a^	Coefficient estimate		Standard error	*P* value
Logical Memory—Immediate Recall	–0.1256	0.0459	6.2×10^–3^	–0.0590		0.0138	2.0×10^–5^
Logical Memory—Delayed Recall	–0.1401	0.0437	1.4×10^–3^	–0.0523		0.0124	2.6×10^–5^
Logical Memory—Recognition	–0.2469	0.1067	2.1×10^–2^	–0.2131		0.0504	2.3×10^–5^
Visual Reproduction—Immediate Recall	–0.3044	0.0712	1.9×10^–5^	–0.0498		0.0116	1.8×10^–5^
Visual Reproduction—Delayed Recall	–0.3558	0.0757	2.6×10^–6^	–0.0465		0.0110	2.2×10^–5^
Visual Reproduction—Recognition	–0.4896	0.1519	1.3×10^–3^	–0.1357		0.0333	4.5×10^–5^
Paired Associate Learning—Immediate Recall	–0.1939	0.0542	3.5×10^–4^	–0.1157		0.0236	9.2×10^–7^
Paired Associate Learning—Delayed Recall	–0.3861	0.1100	4.5×10^–4^	–0.1575		0.0349	6.4×10^–6^
Paired Associate Learning—Recognition	–0.0860	0.0675	2.0×10^–1^	–0.5349		0.1334	6.0×10^–5^
Digit Span—Forward	–0.2922	0.1549	5.9×10^–2^	–0.2417		0.0560	1.6×10^–5^
Digit Span—Backward	–0.6303	0.1858	6.9×10^–4^	–0.2493		0.0551	6.1×10^–6^
Trail Making Test A	0.0022	0.0050	6.6×10^–1^	0.0033		0.0010	1.0×10^–3^
Trail Making Test B	0.0039	0.0012	1.1×10^–3^	0.0010		0.0003	3.3×10^–4^
Similarities	–0.1118	0.0421	7.9×10^–3^	–0.0668		0.0154	1.4×10^–5^
Hooper Visual Organization Test	–0.1030	0.0435	1.8×10^–2^	–0.0449		0.0102	1.2×10^–5^
Boston Naming Test—30-item version	–0.0236	0.0212	2.7×10^–1^	–0.0300		0.0070	1.8×10^–5^
Verbal Fluency	–0.0396	0.0161	1.4×10^–2^	–0.0115		0.0029	6.3×10^–5^
Verbal Fluency—Animal	–0.0727	0.0283	1.0×10^–2^	–0.0366		0.0088	3.1×10^–5^

## Discussion

Neuropsychological tests have been widely used in the assessment of cognitive performance. All 18 neuropsychological tests, for the assessment of multiple cognitive domains, were significantly associated with an average of 50 dCDT features (range 13 to 86 features), and dCDT composite scores were significantly associated with mild cognitive impairment compared to normal cognition.

The CDT examines a wide range of cognitive abilities [5]. The command condition requires that an individual first understand the verbal command, recall all clock related attributes from semantic memory, understand the visuospatial relationships between clock features, and execute the command using necessary mental planning and visuoconstructional abilities. For the copy condition, successful performance requires that an individual recognize the visuospatial attributes in the model to be copied and then marshal the necessary executive abilities to execute output in an organized fashion. However, the standard pencil-and-paper CDT for dementia assessment is usually subjective and time intensive. Given only a limited number of features can be scored, the standard CDT has relatively inferior sensitivity and variable specificity for mild or questionable dementia [2]. On the other hand, the dCDT provides comprehensive and objective assessment of multiple cognitive domains with far greater time efficiency [29,30]. It was reported that the total completion time of dCDT was positively correlated with cognitive functions, whereas the post–clock face latency and pre–first-hand latency were negatively associated with the working memory and processing speed [31]. Our study extended this work by including more than 100 dCDT features and assessing their association with 18 different neuropsychological tests. The composite scores built from dCDT features were significantly associated (all *P*<.001) with multiple neuropsychological tests, such as Trail Making Test A, Trail Making Test B, Hooper Visual Organization Test, and Visual Reproduction subtests. Our results suggested that dCDT composite scores represent better surrogates for their corresponding neuropsychological tests than individual dCDT features. The results also underscore the psychometric characteristics of the dCDT for measuring multiple cognitive domains, such as attention, executive function, visuoperceptual organization, and visual memory, findings which are consistent with those of prior studies [1,5].

A complete neuropsychological test protocol must be administered by a trained rater and interpreted by a neuropsychological expert, which takes at least 45 minutes; therefore, financial and medical resource requirements limit the application of a complete neuropsychological test protocol in general clinics. In contrast, the dCDT is much more convenient, and the test generally takes less than 2 minutes. It is also worth noting that some patients with mild cognitive impairment may show normal performance in some of cognitive domains of neuropsychological test, demonstrating reduced sensitivity of these neuropsychological tests in detecting mild cognitive impairment for some patient groups [32]. As dCDT composite scores were derived from a combination of multiple features associated with neuropsychological tests, they have the potential to identify more subtle cognitive impairment than individual neuropsychological tests. In an earlier study, Dion et al [31] analyzed 202 older adults without dementia and found that participants with mild cognitive impairment tended to take more time to complete the entire test—more “Think than Ink” (ie, percentage of time thinking vs percentage of time drawing)—and drew smaller clock face areas than those drawn by participants with normal cognition. In another study consisting of 138 patients with mild cognitive impairment and amnesia, 106 patients with mild Alzheimer disease, and 137 normal cognition participants; a tablet-based dCDT provided a slightly higher diagnostic accuracy for patients with mild cognitive impairment and amnesia than the CERAD (Consortium to Establish a Registry for Alzheimer's Disease) total score (81.5% vs 77.5%) [33]. The dCDT features have also been used to differentiate between other neurological diseases, such as memory impairment disorders, vascular cognitive disorders, and Parkinson disease [17].

Several study limitations merit consideration. First, this was a cross-sectional study, which cannot reveal temporal relationship between dCDT performance and mild cognitive impairment. It would be interesting to perform longitudinal analysis to investigate early cognitive markers of dCDT features that predict future cognitive decline. Second, only a moderate number of patients with mild cognitive impairment were included. The number of patients with dementia was even smaller, and therefore, patients with dementia were excluded. Third, neuropsychological tests were used to diagnose mild cognitive impairment, which possibly caused some circularity and overestimated diagnostic performance of neuropsychological tests. On the other hand, neither dCDT features nor derived composite scores were used for the mild cognitive impairment diagnosis, which reduced the bias of potential overestimation. Fourth, due to the increasing exposure to digitalized clock displays, a recent study found that some participants drew digital clocks instead of analog clocks required by the test [34]. It is thus important to continue to explore novel cognitive assessment strategies to better capture new features from different neuropsychological tests to avoid potential bias caused by this new technology trend. Finally, yet importantly, FHS participants were mostly of European ancestry and English speakers, therefore, the applicability of these findings to populations of other race and ethnicity is unknown. Notwithstanding these limitations, our study had several strengths. We studied the association between dCDT and a standard epidemiologic neuropsychological test protocol with community-based FHS study data. FHS data have been collected consistently with rigorous quality control and clinical diagnosis by consensus review. Notably, unlike tablet-based apps, the digital pen used in our study offers an almost identical user experience as that of a traditional ballpoint pen; no extra training is needed, which is particularly important for older adult participants who might be unfamiliar with new digital technologies. The performance was thus less likely distorted [10,11].

Associations between dCDT features and standard neuropsychological test data, as well as composite scores from dCDT features as an alternative to neuropsychological tests for the classification of mild cognitive impairment, from more than 2000 participants from a large community-based cohort suggest the potential of dCDT as a cost-effective and easy-to-administer tool for general practitioners, with potential for use in low-resource countries or regions where clinical dementia expertise is limited.

## Appendix

Multimedia Appendix 1 Supplementary tables.

## Abbreviations

CDT: Clock Drawing Test
dCDT: digital Clock Drawing Test
FHS: Framingham Heart Study
