# Supplementary material for: Association Between the Digital Clock Drawing Test and Neuropsychological Test Performance: Large Community-Based Prospective Cohort (Framingham Heart Study)
Source: J Med Internet Res. 2021 Jun 8;23(6):e27407. doi: 10.2196/27407 (PMC8241432; doi:10.2196/27407)
Supplement: Multimedia Appendix 1 [file jmir_v23i6e27407_app1.pdf]

Supplemental Table 1 Association between each dCDT feature and NP tests

NP tests	dCDT features	Effect size	Standard error	*P* value [a]
LMi	DCTScore	0.6911	0.0766	**4.2×10^{-19}**
LMi	COMComponentPlacement_s	0.6192	0.0741	**1.1×10^{-16}**
LMi	COMComponentPlacement	-0.6275	0.0756	**1.9×10^{-16}**
LMi	COMSpatialReasoning_s	0.6085	0.0735	**2.2×10^{-16}**
LMi	COMSpatialReasoning	0.6060	0.0748	**8.9×10^{-16}**
LMi	COMLatencyVariability	-0.5286	0.0740	**1.3×10^{-12}**
LMi	COMInformationProcessing	0.5217	0.0743	**3.0×10^{-12}**
LMi	COMLatencyVariability_s	0.5069	0.0732	**5.7×10^{-12}**
LMi	COMRelativeLongLatency	-0.5094	0.0743	**9.5×10^{-12}**
LMi	COMDrawingEfficiency	0.5038	0.0744	**1.7×10^{-11}**
LMi	COMDrawingProcessEfficiency	0.5029	0.0750	**2.5×10^{-11}**
LMi	COMLongestLatency	-0.4949	0.0738	**2.6×10^{-11}**
LMi	COMInformationProcessing_s	0.4896	0.0735	**3.4×10^{-11}**
LMi	COMRelativeLongLatency_s	0.4801	0.0735	**8.4×10^{-11}**
LMi	COMLongestLatency_s	0.4753	0.0731	**9.7×10^{-11}**
LMi	COMDrawingEfficiency_s	0.4750	0.0738	**1.5×10^{-10}**
LMi	COMDrawingProcessEfficiency_s	0.4605	0.0739	**5.6×10^{-10}**
LMi	COMPercentInkTime	0.4581	0.0737	**6.3×10^{-10}**
LMi	COMLongLatencyCount_s	0.4516	0.0737	**1.1×10^{-9}**
LMi	COMPercentThinkTime	-0.4476	0.0738	**1.6×10^{-9}**
LMi	COMLongLatencyCount	-0.4600	0.0770	**2.7×10^{-9}**
LMi	COMAverageLatency	-0.4490	0.0754	**3.0×10^{-9}**
LMi	COMPercentThinkTime_s	0.4368	0.0738	**3.7×10^{-9}**
LMi	COMPercentInkTime_s	-0.4261	0.0739	**9.2×10^{-9}**
LMi	COMAverageLatency_s	0.4176	0.0741	**2.0×10^{-8}**
LMi	COPComponentPlacement_s	0.3802	0.0737	**2.7×10^{-7}**
LMi	COPComponentPlacement	-0.3825	0.0752	**3.9×10^{-7}**
LMi	COPSpatialReasoning	0.3642	0.0749	**1.3×10^{-6}**
LMi	COPLatencyVariability	-0.3652	0.0757	**1.5×10^{-6}**
LMi	COPLongestLatency	-0.3555	0.0754	**2.6×10^{-6}**
LMi	COPLongLatencyCount	-0.3633	0.0780	**3.4×10^{-6}**
LMi	COPSpatialReasoning_s	0.3410	0.0739	**4.2×10^{-6}**
LMi	COPInformationProcessing	0.3470	0.0760	**5.2×10^{-6}**
LMi	COPRelativeLongLatency	-0.3462	0.0762	**5.9×10^{-6}**
LMi	COMDrawingSize	0.3302	0.0740	**8.5×10^{-6}**
LMi	COMTotalTime	-0.3356	0.0756	**9.5×10^{-6}**
LMi	COMDrawingSize_s	0.3212	0.0739	**1.4×10^{-5}**
LMi	COPDrawingSize_s	0.3047	0.0734	**3.4×10^{-5}**
LMi	COMTotalTime_s	0.3066	0.0741	**3.7×10^{-5}**
LMi	COPLatencyVariability_s	0.3084	0.0750	**4.1×10^{-5}**

LMi	COPLongestLatency_s	0.3060	0.0747	**4.3×10^{-5}**
LMi	COPOscillatoryMotion	-0.3083	0.0762	**5.4×10^{-5}**
LMi	COPOscillatoryMotion_s	0.2963	0.0741	**6.5×10^{-5}**
LMi	COPDrawingSize	0.2924	0.0735	**7.2×10^{-5}**
LMi	COPInformationProcessing_s	0.2942	0.0753	**9.6×10^{-5}**
LMi	COPRelativeLongLatency_s	0.2942	0.0754	**9.9×10^{-5}**
LMi	COPDrawingEfficiency	0.2679	0.0756	**4.0×10^{-4}**
LMi	COMOscillatoryMotion_s	0.2684	0.0760	**4.2×10^{-4}**
LMi	COPSimpleMotor	0.2579	0.0753	6.2×10^{-4}
LMi	COPLongLatencyCount_s	0.2533	0.0743	6.7×10^{-4}
LMi	COPClockfaceCircularity_s	0.2482	0.0735	7.5×10^{-4}
LMi	COMOscillatoryMotion	-0.2613	0.0786	9.0×10^{-4}
LMi	COPDrawingEfficiency_s	0.2401	0.0749	1.4×10^{-3}
LMi	COPAverageLatency	-0.2435	0.0764	1.4×10^{-3}
LMi	COPDrawingProcessEfficiency	0.2367	0.0756	1.8×10^{-3}
LMi	COMVerticalSpatialPlacement_s	0.2265	0.0731	2.0×10^{-3}
LMi	COMSimpleMotor	0.2302	0.0758	2.4×10^{-3}
LMi	COPClockfaceCircularity	-0.2211	0.0737	2.7×10^{-3}
LMi	COMInkLength	0.2225	0.0746	2.9×10^{-3}
LMi	COMInkLength_s	0.2223	0.0746	2.9×10^{-3}
LMi	COPTotalTime	-0.2257	0.0773	3.6×10^{-3}
LMi	COPSimpleMotor_s	0.2128	0.0736	3.9×10^{-3}
LMi	COMNoise	-0.2082	0.0763	6.4×10^{-3}
LMi	COPDrawingProcessEfficiency_s	0.1979	0.0748	8.2×10^{-3}
LMi	COMSimpleMotor_s	0.1956	0.0746	8.8×10^{-3}
LMi	COPAverageLatency_s	0.1888	0.0753	1.2×10^{-2}
LMi	COMVerticalSpatialPlacement	-0.1836	0.0733	1.2×10^{-2}
LMi	COMNoise_s	0.1840	0.0747	1.4×10^{-2}
LMi	COPPercentInkTime	0.1822	0.0748	1.5×10^{-2}
LMi	COPPercentThinkTime	-0.1764	0.0750	1.9×10^{-2}
LMi	COPPercentThinkTime_s	0.1700	0.0749	2.3×10^{-2}
LMi	COMHorizontalSpatialPlacement_s	0.1620	0.0731	2.7×10^{-2}
LMi	COPPercentInkTime_s	-0.1654	0.0750	2.8×10^{-2}
LMi	COPHorizontalSpatialPlacement	-0.1553	0.0734	3.4×10^{-2}
LMi	COPTotalTime_s	0.1574	0.0752	3.6×10^{-2}
LMi	COPHorizontalSpatialPlacement_s	0.1439	0.0734	5.0×10^{-2}
LMi	COMHorizontalSpatialPlacement	-0.1249	0.0732	8.8×10^{-2}
LMi	COPStrokeCountConformity	-0.1355	0.0808	9.4×10^{-2}
LMi	COMInitiationSpeed	0.1206	0.0735	1.0×10^{-1}
LMi	COPVerticalSpatialPlacement	-0.1185	0.0739	1.1×10^{-1}
LMi	COPVerticalSpatialPlacement_s	0.1097	0.0736	1.4×10^{-1}
LMi	COMClockfaceCircularity_s	0.1023	0.0735	1.6×10^{-1}
LMi	COMStrokeCountConformity	-0.1060	0.0775	1.7×10^{-1}

LMi	COMAverageSpeed	0.0985	0.0737	1.8×10^{-1}
LMi	COMInitiationSpeed_s	0.0982	0.0735	1.8×10^{-1}
LMi	COPInitiationSpeed	0.0929	0.0736	2.1×10^{-1}
LMi	COPAverageSpeed	0.0832	0.0738	2.6×10^{-1}
LMi	COMClockfaceCircularity	-0.0820	0.0742	2.7×10^{-1}
LMi	COPTerminationSpeed	0.0796	0.0740	2.8×10^{-1}
LMi	COPMaxSpeed	0.0783	0.0734	2.9×10^{-1}
LMi	COMTerminationSpeed	0.0723	0.0737	3.3×10^{-1}
LMi	COPNoise	-0.0746	0.0760	3.3×10^{-1}
LMi	COMMaxSpeed	0.0696	0.0733	3.4×10^{-1}
LMi	COMAverageSpeed_s	0.0677	0.0736	3.6×10^{-1}
LMi	COPStrokeCountConformity_s	-0.0658	0.0742	3.8×10^{-1}
LMi	COPInitiationSpeed_s	0.0517	0.0734	4.8×10^{-1}
LMi	COMTerminationSpeed_s	0.0445	0.0736	5.5×10^{-1}
LMi	COPTerminationSpeed_s	0.0428	0.0737	5.6×10^{-1}
LMi	COMMaxSpeed_s	0.0404	0.0733	5.8×10^{-1}
LMi	COPAverageSpeed_s	0.0378	0.0735	6.1×10^{-1}
LMi	COPMaxSpeed_s	0.0328	0.0732	6.5×10^{-1}
LMi	COMStrokeCountConformity_s	-0.0294	0.0742	6.9×10^{-1}
LMi	COPInkLength_s	0.0232	0.0739	7.5×10^{-1}
LMi	COPInkLength	0.0232	0.0744	7.6×10^{-1}
LMi	COPNoise_s	0.0181	0.0751	8.1×10^{-1}
LMd	DCTScore	0.7082	0.0810	**4.8×10^{-18}**
LMd	COMComponentPlacement_s	0.6352	0.0783	**8.4×10^{-16}**
LMd	COMSpatialReasoning_s	0.6228	0.0777	**1.8×10^{-15}**
LMd	COMComponentPlacement	-0.6380	0.0799	**2.4×10^{-15}**
LMd	COMSpatialReasoning	0.6167	0.0790	**9.6×10^{-15}**
LMd	COMLatencyVariability	-0.5227	0.0784	**3.3×10^{-11}**
LMd	COMRelativeLongLatency	-0.5207	0.0786	**4.4×10^{-11}**
LMd	COMInformationProcessing	0.5185	0.0786	**5.5×10^{-11}**
LMd	COMDrawingProcessEfficiency	0.5108	0.0792	**1.4×10^{-10}**
LMd	COMLatencyVariability_s	0.4968	0.0774	**1.7×10^{-10}**
LMd	COMInformationProcessing_s	0.4925	0.0777	**2.9×10^{-10}**
LMd	COMLongestLatency	-0.4928	0.0781	**3.5×10^{-10}**
LMd	COMLongLatencyCount_s	0.4911	0.0779	**3.6×10^{-10}**
LMd	COMDrawingEfficiency	0.4959	0.0787	**3.6×10^{-10}**
LMd	COMRelativeLongLatency_s	0.4866	0.0778	**4.8×10^{-10}**
LMd	COMLongLatencyCount	-0.4967	0.0814	**1.3×10^{-9}**
LMd	COMDrawingEfficiency_s	0.4731	0.0780	**1.6×10^{-9}**
LMd	COMLongestLatency_s	0.4686	0.0773	**1.6×10^{-9}**
LMd	COMDrawingProcessEfficiency_s	0.4703	0.0781	**2.1×10^{-9}**
LMd	COMAverageLatency	-0.4503	0.0797	**1.8×10^{-8}**

LMd	COMPercentInkTime	0.4344	0.0781	**3.0×10^{-8}**
LMd	COMPercentThinkTime	-0.4299	0.0782	**4.3×10^{-8}**
LMd	COPLongLatencyCount	-0.4507	0.0821	**4.6×10^{-8}**
LMd	COPComponentPlacement_s	0.4192	0.0778	**7.9×10^{-8}**
LMd	COMAverageLatency_s	0.4179	0.0783	**1.1×10^{-7}**
LMd	COPComponentPlacement	-0.4189	0.0794	**1.4×10^{-7}**
LMd	COMPercentThinkTime_s	0.4108	0.0782	**1.6×10^{-7}**
LMd	COMPercentInkTime_s	-0.4061	0.0782	**2.3×10^{-7}**
LMd	COPInformationProcessing	0.3936	0.0802	**1.0×10^{-6}**
LMd	COPLatencyVariability	-0.3914	0.0800	**1.1×10^{-6}**
LMd	COPSpatialReasoning	0.3815	0.0791	**1.5×10^{-6}**
LMd	COPLongestLatency	-0.3824	0.0797	**1.7×10^{-6}**
LMd	COPRelativeLongLatency	-0.3813	0.0805	**2.3×10^{-6}**
LMd	COMTotalTime	-0.3706	0.0799	**3.7×10^{-6}**
LMd	COPSpatialReasoning_s	0.3619	0.0781	**3.8×10^{-6}**
LMd	COPLongLatencyCount_s	0.3489	0.0783	**8.9×10^{-6}**
LMd	COMTotalTime_s	0.3416	0.0784	**1.4×10^{-5}**
LMd	COPInformationProcessing_s	0.3446	0.0794	**1.5×10^{-5}**
LMd	COPLongestLatency_s	0.3366	0.0789	**2.1×10^{-5}**
LMd	COPLatencyVariability_s	0.3381	0.0793	**2.1×10^{-5}**
LMd	COPRelativeLongLatency_s	0.3359	0.0797	**2.6×10^{-5}**
LMd	COMDrawingSize	0.3122	0.0782	**6.8×10^{-5}**
LMd	COPSimpleMotor	0.3119	0.0795	**9.1×10^{-5}**
LMd	COPDrawingSize_s	0.3030	0.0775	**9.5×10^{-5}**
LMd	COMDrawingSize_s	0.3012	0.0781	**1.2×10^{-4}**
LMd	COPDrawingSize	0.2947	0.0776	**1.5×10^{-4}**
LMd	COPDrawingProcessEfficiency	0.3003	0.0798	**1.7×10^{-4}**
LMd	COPDrawingEfficiency	0.2990	0.0798	**1.8×10^{-4}**
LMd	COPAverageLatency	-0.3001	0.0806	**2.0×10^{-4}**
LMd	COPTotalTime	-0.2983	0.0816	**2.6×10^{-4}**
LMd	COPDrawingEfficiency_s	0.2834	0.0789	**3.4×10^{-4}**
LMd	COPOscillatoryMotion_s	0.2804	0.0784	**3.6×10^{-4}**
LMd	COPOscillatoryMotion	-0.2875	0.0807	**3.8×10^{-4}**
LMd	COPSimpleMotor_s	0.2739	0.0777	**4.4×10^{-4}**
LMd	COMSimpleMotor	0.2764	0.0800	5.6×10^{-4}
LMd	COPDrawingProcessEfficiency_s	0.2693	0.0789	6.6×10^{-4}
LMd	COMNoise	-0.2579	0.0805	1.4×10^{-3}
LMd	COMVerticalSpatialPlacement_s	0.2469	0.0771	1.4×10^{-3}
LMd	COPAverageLatency_s	0.2517	0.0794	1.6×10^{-3}
LMd	COMOscillatoryMotion_s	0.2529	0.0804	1.7×10^{-3}
LMd	COMSimpleMotor_s	0.2460	0.0788	1.8×10^{-3}
LMd	COPTotalTime_s	0.2430	0.0794	2.2×10^{-3}
LMd	COMNoise_s	0.2239	0.0789	4.6×10^{-3}

LMd	COMOscillatoryMotion	-0.2359	0.0832	4.6×10^{-3}
LMd	COMVerticalSpatialPlacement	-0.2081	0.0773	7.1×10^{-3}
LMd	COPClockfaceCircularity_s	0.2084	0.0778	7.4×10^{-3}
LMd	COMInkLength	0.1947	0.0788	1.3×10^{-2}
LMd	COMInkLength_s	0.1938	0.0788	1.4×10^{-2}
LMd	COPClockfaceCircularity	-0.1783	0.0780	2.2×10^{-2}
LMd	COPTerminationSpeed	0.1786	0.0783	2.3×10^{-2}
LMd	COPPercentInkTime	0.1787	0.0790	2.4×10^{-2}
LMd	COPMaxSpeed	0.1746	0.0776	2.5×10^{-2}
LMd	COPStrokeCountConformity	-0.1912	0.0853	2.5×10^{-2}
LMd	COPAverageSpeed	0.1747	0.0781	2.5×10^{-2}
LMd	COPHorizontalSpatialPlacement_s	0.1736	0.0776	2.5×10^{-2}
LMd	COPHorizontalSpatialPlacement	-0.1710	0.0776	2.8×10^{-2}
LMd	COPPercentThinkTime	-0.1741	0.0792	2.8×10^{-2}
LMd	COMAverageSpeed	0.1660	0.0778	3.3×10^{-2}
LMd	COPPercentThinkTime_s	0.1685	0.0791	3.3×10^{-2}
LMd	COPInitiationSpeed	0.1646	0.0778	3.4×10^{-2}
LMd	COMTerminationSpeed	0.1639	0.0779	3.5×10^{-2}
LMd	COPPercentInkTime_s	-0.1654	0.0793	3.7×10^{-2}
LMd	COMInitiationSpeed	0.1601	0.0776	3.9×10^{-2}
LMd	COMMaxSpeed	0.1563	0.0774	4.4×10^{-2}
LMd	COPTerminationSpeed_s	0.1445	0.0780	6.4×10^{-2}
LMd	COMInitiationSpeed_s	0.1407	0.0776	7.0×10^{-2}
LMd	COMStrokeCountConformity	-0.1485	0.0820	7.0×10^{-2}
LMd	COMAverageSpeed_s	0.1400	0.0777	7.2×10^{-2}
LMd	COMTerminationSpeed_s	0.1387	0.0777	7.5×10^{-2}
LMd	COPAverageSpeed_s	0.1346	0.0777	8.4×10^{-2}
LMd	COPMaxSpeed_s	0.1323	0.0774	8.8×10^{-2}
LMd	COMMaxSpeed_s	0.1289	0.0774	9.6×10^{-2}
LMd	COPInitiationSpeed_s	0.1280	0.0776	9.9×10^{-2}
LMd	COMHorizontalSpatialPlacement_s	0.1250	0.0773	1.1×10^{-1}
LMd	COPVerticalSpatialPlacement	-0.1065	0.0779	1.7×10^{-1}
LMd	COPVerticalSpatialPlacement_s	0.1055	0.0778	1.7×10^{-1}
LMd	COPNoise	-0.1026	0.0803	2.0×10^{-1}
LMd	COMHorizontalSpatialPlacement	-0.0864	0.0774	2.6×10^{-1}
LMd	COPNoise_s	0.0859	0.0792	2.8×10^{-1}
LMd	COMStrokeCountConformity_s	0.0811	0.0785	3.0×10^{-1}
LMd	COMClockfaceCircularity_s	0.0694	0.0777	3.7×10^{-1}
LMd	COMClockfaceCircularity	-0.0448	0.0784	5.7×10^{-1}
LMd	COPStrokeCountConformity_s	0.0290	0.0784	7.1×10^{-1}
LMd	COPInkLength	-0.0041	0.0786	9.6×10^{-1}
LMd	COPInkLength_s	-0.0036	0.0781	9.6×10^{-1}

LMr	COMComponentPlacement	-0.2135	0.0300	**1.5×10^{-12}**
LMr	COMSpatialReasoning	0.2100	0.0296	**1.9×10^{-12}**
LMr	COMSpatialReasoning_s	0.2048	0.0292	**3.1×10^{-12}**
LMr	COMComponentPlacement_s	0.2060	0.0294	**3.4×10^{-12}**
LMr	DCTScore	0.2047	0.0306	**2.7×10^{-11}**
LMr	COMDrawingProcessEfficiency	0.1842	0.0296	**6.2×10^{-10}**
LMr	COMDrawingProcessEfficiency_s	0.1776	0.0292	**1.4×10^{-9}**
LMr	COMDrawingEfficiency	0.1792	0.0295	**1.4×10^{-9}**
LMr	COMDrawingEfficiency_s	0.1746	0.0292	**2.6×10^{-9}**
LMr	COMInformationProcessing	0.1745	0.0295	**3.7×10^{-9}**
LMr	COMRelativeLongLatency	-0.1736	0.0295	**4.5×10^{-9}**
LMr	COMLongLatencyCount	-0.1777	0.0304	**6.2×10^{-9}**
LMr	COMInformationProcessing_s	0.1696	0.0291	**6.5×10^{-9}**
LMr	COMRelativeLongLatency_s	0.1689	0.0291	**7.8×10^{-9}**
LMr	COMAverageLatency	-0.1664	0.0298	**2.6×10^{-8}**
LMr	COMAverageLatency_s	0.1626	0.0292	**3.1×10^{-8}**
LMr	COMLongLatencyCount_s	0.1606	0.0292	**4.4×10^{-8}**
LMr	COMLatencyVariability	-0.1607	0.0294	**5.3×10^{-8}**
LMr	COMLatencyVariability_s	0.1581	0.0291	**5.9×10^{-8}**
LMr	COMLongestLatency	-0.1471	0.0293	**5.8×10^{-7}**
LMr	COMTotalTime	-0.1496	0.0298	**5.9×10^{-7}**
LMr	COMLongestLatency_s	0.1448	0.0290	**6.6×10^{-7}**
LMr	COMTotalTime_s	0.1444	0.0293	**8.7×10^{-7}**
LMr	COMPercentInkTime	0.1320	0.0292	**6.7×10^{-6}**
LMr	COMPercentThinkTime	-0.1299	0.0293	**9.6×10^{-6}**
LMr	COMPercentThinkTime_s	0.1284	0.0292	**1.2×10^{-5}**
LMr	COMPercentInkTime_s	-0.1261	0.0293	**1.7×10^{-5}**
LMr	COPLongLatencyCount	-0.1123	0.0308	**2.8×10^{-4}**
LMr	COPComponentPlacement_s	0.0959	0.0292	1.0×10^{-3}
LMr	COPComponentPlacement	-0.0977	0.0298	1.1×10^{-3}
LMr	COPSimpleMotor	0.0937	0.0298	1.7×10^{-3}
LMr	COPLongLatencyCount_s	0.0915	0.0293	1.8×10^{-3}
LMr	COPSimpleMotor_s	0.0859	0.0291	3.1×10^{-3}
LMr	COPInformationProcessing	0.0863	0.0301	4.2×10^{-3}
LMr	COPSpatialReasoning	0.0846	0.0297	4.4×10^{-3}
LMr	COMAverageSpeed	0.0820	0.0291	4.8×10^{-3}
LMr	COMSimpleMotor	0.0842	0.0299	5.0×10^{-3}
LMr	COPSpatialReasoning_s	0.0806	0.0293	6.0×10^{-3}
LMr	COPDrawingEfficiency	0.0818	0.0298	6.2×10^{-3}
LMr	COMAverageSpeed_s	0.0785	0.0290	6.8×10^{-3}
LMr	COPInformationProcessing_s	0.0805	0.0298	6.9×10^{-3}
LMr	COMInitiationSpeed	0.0782	0.0290	7.0×10^{-3}
LMr	COMTerminationSpeed	0.0777	0.0291	7.5×10^{-3}

LMr	COMDrawingSize_s	0.0785	0.0294	7.6×10^{-3}
LMr	COMDrawingSize	0.0785	0.0294	7.7×10^{-3}
LMr	COMSimpleMotor_s	0.0786	0.0295	7.7×10^{-3}
LMr	COMInitiationSpeed_s	0.0763	0.0290	8.6×10^{-3}
LMr	COPRelativeLongLatency_s	0.0784	0.0299	8.8×10^{-3}
LMr	COPDrawingEfficiency_s	0.0768	0.0295	9.3×10^{-3}
LMr	COPLatencyVariability	-0.0777	0.0300	9.7×10^{-3}
LMr	COPRelativeLongLatency	-0.0783	0.0302	9.7×10^{-3}
LMr	COMNoise	-0.0779	0.0301	9.8×10^{-3}
LMr	COPLatencyVariability_s	0.0756	0.0297	1.1×10^{-2}
LMr	COPTerminationSpeed	0.0741	0.0291	1.1×10^{-2}
LMr	COMTerminationSpeed_s	0.0733	0.0290	1.2×10^{-2}
LMr	COPDrawingSize_s	0.0729	0.0290	1.2×10^{-2}
LMr	COPDrawingSize	0.0721	0.0291	1.3×10^{-2}
LMr	COMStrokeCountConformity	-0.0762	0.0308	1.3×10^{-2}
LMr	COMNoise_s	0.0727	0.0294	1.4×10^{-2}
LMr	COPOscillatoryMotion_s	0.0722	0.0293	1.4×10^{-2}
LMr	COMMaxSpeed	0.0705	0.0289	1.5×10^{-2}
LMr	COPLongestLatency	-0.0715	0.0299	1.7×10^{-2}
LMr	COPTerminationSpeed_s	0.0692	0.0290	1.7×10^{-2}
LMr	COPLongestLatency_s	0.0704	0.0296	1.7×10^{-2}
LMr	COMMaxSpeed_s	0.0676	0.0289	1.9×10^{-2}
LMr	COPAverageLatency_s	0.0686	0.0297	2.1×10^{-2}
LMr	COPDrawingProcessEfficiency	0.0690	0.0299	2.1×10^{-2}
LMr	COPAverageLatency	-0.0680	0.0302	2.4×10^{-2}
LMr	COPAverageSpeed	0.0653	0.0291	2.5×10^{-2}
LMr	COPMaxSpeed	0.0625	0.0289	3.1×10^{-2}
LMr	COPTotalTime	-0.0657	0.0306	3.2×10^{-2}
LMr	COPDrawingProcessEfficiency_s	0.0631	0.0296	3.3×10^{-2}
LMr	COPInitiationSpeed	0.0619	0.0290	3.3×10^{-2}
LMr	COPTotalTime_s	0.0623	0.0297	3.6×10^{-2}
LMr	COMVerticalSpatialPlacement_s	0.0607	0.0289	3.6×10^{-2}
LMr	COPAverageSpeed_s	0.0593	0.0289	4.1×10^{-2}
LMr	COPHorizontalSpatialPlacement_s	0.0590	0.0291	4.3×10^{-2}
LMr	COPMaxSpeed_s	0.0563	0.0288	5.1×10^{-2}
LMr	COPInitiationSpeed_s	0.0549	0.0289	5.8×10^{-2}
LMr	COMInkLength	0.0554	0.0295	6.1×10^{-2}
LMr	COMInkLength_s	0.0550	0.0295	6.3×10^{-2}
LMr	COMClockfaceCircularity_s	0.0542	0.0291	6.3×10^{-2}
LMr	COMOscillatoryMotion_s	0.0555	0.0301	6.6×10^{-2}
LMr	COPStrokeCountConformity	-0.0570	0.0320	7.5×10^{-2}
LMr	COPOscillatoryMotion	-0.0513	0.0302	9.0×10^{-2}
LMr	COMHorizontalSpatialPlacement_s	0.0485	0.0289	9.4×10^{-2}

LMr	COMStrokeCountConformity_s	0.0472	0.0293	1.1×10^{-1}
LMr	COPClockfaceCircularity_s	0.0466	0.0291	1.1×10^{-1}
LMr	COPPercentInkTime	0.0393	0.0296	1.8×10^{-1}
LMr	COPHorizontalSpatialPlacement	-0.0379	0.0291	1.9×10^{-1}
LMr	COPPercentThinkTime_s	0.0355	0.0296	2.3×10^{-1}
LMr	COPPercentThinkTime	-0.0309	0.0297	3.0×10^{-1}
LMr	COPNoise	-0.0291	0.0301	3.3×10^{-1}
LMr	COMVerticalSpatialPlacement	-0.0274	0.0290	3.4×10^{-1}
LMr	COPPercentInkTime_s	-0.0274	0.0297	3.6×10^{-1}
LMr	COPVerticalSpatialPlacement_s	0.0219	0.0290	4.5×10^{-1}
LMr	COMOscillatoryMotion	-0.0228	0.0312	4.6×10^{-1}
LMr	COMClockfaceCircularity	-0.0200	0.0294	5.0×10^{-1}
LMr	COPNoise_s	0.0184	0.0296	5.3×10^{-1}
LMr	COPClockfaceCircularity	-0.0178	0.0292	5.4×10^{-1}
LMr	COMHorizontalSpatialPlacement	-0.0154	0.0289	5.9×10^{-1}
LMr	COPInkLength	-0.0057	0.0294	8.5×10^{-1}
LMr	COPStrokeCountConformity_s	-0.0029	0.0293	9.2×10^{-1}
LMr	COPInkLength_s	-0.0028	0.0292	9.2×10^{-1}
LMr	COPVerticalSpatialPlacement	-0.0026	0.0291	9.3×10^{-1}
VRi	DCTScore	0.7669	0.0583	**4.9×10^{-38}**
VRi	COMComponentPlacement_s	0.7314	0.0561	**2.3×10^{-37}**
VRi	COMComponentPlacement	-0.7445	0.0573	**3.5×10^{-37}**
VRi	COMSpatialReasoning	0.7222	0.0567	**8.1×10^{-36}**
VRi	COMSpatialReasoning_s	0.7094	0.0558	**1.1×10^{-35}**
VRi	COPComponentPlacement	-0.5026	0.0576	**5.1×10^{-18}**
VRi	COPComponentPlacement_s	0.4872	0.0565	**1.3×10^{-17}**
VRi	COPSpatialReasoning	0.4737	0.0574	**2.8×10^{-16}**
VRi	COMDrawingProcessEfficiency	0.4733	0.0579	**4.9×10^{-16}**
VRi	COMInformationProcessing	0.4563	0.0573	**2.8×10^{-15}**
VRi	COMDrawingEfficiency	0.4574	0.0575	**2.8×10^{-15}**
VRi	COPSpatialReasoning_s	0.4491	0.0567	**3.8×10^{-15}**
VRi	COMLatencyVariability	-0.4515	0.0571	**4.4×10^{-15}**
VRi	COMDrawingEfficiency_s	0.4430	0.0570	**1.2×10^{-14}**
VRi	COMDrawingProcessEfficiency_s	0.4422	0.0571	**1.4×10^{-14}**
VRi	COMInformationProcessing_s	0.4359	0.0566	**2.1×10^{-14}**
VRi	COMLatencyVariability_s	0.4317	0.0564	**3.1×10^{-14}**
VRi	COMRelativeLongLatency	-0.4358	0.0573	**4.4×10^{-14}**
VRi	COMAverageLatency	-0.4396	0.0580	**5.4×10^{-14}**
VRi	COMLongLatencyCount_s	0.4307	0.0569	**5.6×10^{-14}**
VRi	COMLongestLatency	-0.4230	0.0570	**1.6×10^{-13}**
VRi	COMAverageLatency_s	0.4187	0.0570	**2.9×10^{-13}**
VRi	COMRelativeLongLatency_s	0.4131	0.0567	**4.5×10^{-13}**

VRi	COMLongestLatency_s	0.4034	0.0564	**1.2×10^{-12}**
VRi	COMTotalTime	-0.4158	0.0581	**1.2×10^{-12}**
VRi	COMOscillatoryMotion	-0.4237	0.0603	**2.8×10^{-12}**
VRi	COMOscillatoryMotion_s	0.4073	0.0583	**3.9×10^{-12}**
VRi	COMTotalTime_s	0.3901	0.0570	**1.0×10^{-11}**
VRi	COPDrawingEfficiency	0.3961	0.0580	**1.1×10^{-11}**
VRi	COPInformationProcessing	0.3926	0.0584	**2.4×10^{-11}**
VRi	COPTotalTime	-0.3904	0.0594	**6.3×10^{-11}**
VRi	COPDrawingEfficiency_s	0.3772	0.0575	**6.7×10^{-11}**
VRi	COPDrawingProcessEfficiency	0.3803	0.0581	**7.3×10^{-11}**
VRi	COPRelativeLongLatency	-0.3771	0.0588	**1.7×10^{-10}**
VRi	COMClockfaceCircularity_s	0.3609	0.0564	**2.0×10^{-10}**
VRi	COMLongLatencyCount	-0.3813	0.0596	**2.0×10^{-10}**
VRi	COPTotalTime_s	0.3649	0.0578	**3.4×10^{-10}**
VRi	COPDrawingProcessEfficiency_s	0.3598	0.0575	**4.8×10^{-10}**
VRi	COPLatencyVariability	-0.3615	0.0584	**7.1×10^{-10}**
VRi	COPOscillatoryMotion_s	0.3518	0.0570	**7.9×10^{-10}**
VRi	COPRelativeLongLatency_s	0.3579	0.0582	**9.1×10^{-10}**
VRi	COPInformationProcessing_s	0.3564	0.0580	**9.7×10^{-10}**
VRi	COPAverageLatency	-0.3606	0.0587	**9.8×10^{-10}**
VRi	COMClockfaceCircularity	-0.3492	0.0569	**1.0×10^{-9}**
VRi	COPSimpleMotor	0.3518	0.0580	**1.5×10^{-9}**
VRi	COPOscillatoryMotion	-0.3540	0.0587	**1.9×10^{-9}**
VRi	COMSimpleMotor	0.3497	0.0583	**2.3×10^{-9}**
VRi	COPLongestLatency	-0.3477	0.0581	**2.6×10^{-9}**
VRi	COPAverageLatency_s	0.3426	0.0579	**3.8×10^{-9}**
VRi	COPLatencyVariability_s	0.3400	0.0579	**4.9×10^{-9}**
VRi	COPLongLatencyCount_s	0.3316	0.0573	**8.4×10^{-9}**
VRi	COPLongestLatency_s	0.3297	0.0576	**1.2×10^{-8}**
VRi	COMSimpleMotor_s	0.3178	0.0574	**3.5×10^{-8}**
VRi	COPSimpleMotor_s	0.3106	0.0567	**4.9×10^{-8}**
VRi	COPLongLatencyCount	-0.3079	0.0603	**3.6×10^{-7}**
VRi	COPClockfaceCircularity_s	0.2737	0.0567	**1.5×10^{-6}**
VRi	COMNoise_s	0.2737	0.0576	**2.1×10^{-6}**
VRi	COPClockfaceCircularity	-0.2413	0.0568	**2.3×10^{-5}**
VRi	COMVerticalSpatialPlacement_s	0.2200	0.0565	**1.0×10^{-4}**
VRi	COMNoise	-0.2229	0.0590	**1.6×10^{-4}**
VRi	COMInitiationSpeed	0.2128	0.0567	**1.8×10^{-4}**
VRi	COMInitiationSpeed_s	0.2032	0.0567	**3.4×10^{-4}**
VRi	COMVerticalSpatialPlacement	-0.1949	0.0566	5.8×10^{-4}
VRi	COMPercentInkTime	0.1893	0.0574	9.9×10^{-4}
VRi	COMPercentThinkTime	-0.1871	0.0574	1.1×10^{-3}
VRi	COPDrawingSize_s	0.1849	0.0568	1.1×10^{-3}

VRi	COMDrawingSize_s	0.1864	0.0574	1.2×10^{-3}
VRi	COMHorizontalSpatialPlacement_s	0.1826	0.0565	1.3×10^{-3}
VRi	COMAverageSpeed	0.1834	0.0569	1.3×10^{-3}
VRi	COMPercentThinkTime_s	0.1842	0.0574	1.3×10^{-3}
VRi	COMDrawingSize	0.1842	0.0575	1.4×10^{-3}
VRi	COMPercentInkTime_s	-0.1819	0.0574	1.6×10^{-3}
VRi	COPDrawingSize	0.1789	0.0568	1.7×10^{-3}
VRi	COMTerminationSpeed	0.1784	0.0569	1.7×10^{-3}
VRi	COPInitiationSpeed	0.1754	0.0569	2.1×10^{-3}
VRi	COPVerticalSpatialPlacement_s	0.1728	0.0567	2.4×10^{-3}
VRi	COMAverageSpeed_s	0.1695	0.0568	2.9×10^{-3}
VRi	COMHorizontalSpatialPlacement	-0.1668	0.0566	3.2×10^{-3}
VRi	COMTerminationSpeed_s	0.1638	0.0568	4.0×10^{-3}
VRi	COPTerminationSpeed	0.1598	0.0572	5.2×10^{-3}
VRi	COPAverageSpeed	0.1566	0.0571	6.2×10^{-3}
VRi	COPInitiationSpeed_s	0.1531	0.0567	7.0×10^{-3}
VRi	COPVerticalSpatialPlacement	-0.1488	0.0569	8.9×10^{-3}
VRi	COPTerminationSpeed_s	0.1411	0.0570	1.3×10^{-2}
VRi	COPPercentInkTime	0.1426	0.0580	1.4×10^{-2}
VRi	COPPercentThinkTime_s	0.1421	0.0581	1.5×10^{-2}
VRi	COPAverageSpeed_s	0.1325	0.0568	2.0×10^{-2}
VRi	COPPercentThinkTime	-0.1257	0.0582	3.1×10^{-2}
VRi	COPPercentInkTime_s	-0.1248	0.0583	3.2×10^{-2}
VRi	COMStrokeCountConformity_s	0.1184	0.0574	3.9×10^{-2}
VRi	COMInkLength_s	0.1168	0.0577	4.3×10^{-2}
VRi	COMStrokeCountConformity	-0.1219	0.0604	4.4×10^{-2}
VRi	COPNoise_s	0.1170	0.0580	4.4×10^{-2}
VRi	COMInkLength	0.1124	0.0577	5.2×10^{-2}
VRi	COPStrokeCountConformity_s	0.1071	0.0573	6.2×10^{-2}
VRi	COPNoise	-0.1089	0.0589	6.4×10^{-2}
VRi	COMMaxSpeed	0.0947	0.0568	9.5×10^{-2}
VRi	COMMaxSpeed_s	0.0809	0.0567	1.5×10^{-1}
VRi	COPMaxSpeed	0.0690	0.0569	2.3×10^{-1}
VRi	COPStrokeCountConformity	-0.0617	0.0629	3.3×10^{-1}
VRi	COPMaxSpeed_s	0.0449	0.0567	4.3×10^{-1}
VRi	COPInkLength_s	0.0307	0.0573	5.9×10^{-1}
VRi	COPInkLength	0.0232	0.0576	6.9×10^{-1}
VRi	COPHorizontalSpatialPlacement_s	0.0154	0.0570	7.9×10^{-1}
VRi	COPHorizontalSpatialPlacement	0.0092	0.0569	8.7×10^{-1}
VRd	DCTScore	0.8440	0.0634	$\mathbf{8.2\times10^{-39}}$
VRd	COMComponentPlacement_s	0.7997	0.0611	$\mathbf{1.3\times10^{-37}}$
VRd	COMComponentPlacement	-0.7990	0.0625	$\mathbf{5.1\times10^{-36}}$

VRd	COMSpatialReasoning_s	0.7519	0.0609	**8.7×10-34**
VRd	COMSpatialReasoning	0.7542	0.0620	**6.7×10-33**
VRd	COPSpatialReasoning	0.5409	0.0625	**9.6×10^{-18}**
VRd	COPSpatialReasoning_s	0.5170	0.0617	**9.3×10^{-17}**
VRd	COPComponentPlacement	-0.5027	0.0630	**2.3×10^{-15}**
VRd	COPComponentPlacement_s	0.4898	0.0617	**3.5×10^{-15}**
VRd	COMDrawingEfficiency	0.4886	0.0626	**9.4×10^{-15}**
VRd	COMDrawingProcessEfficiency	0.4841	0.0631	**2.6×10^{-14}**
VRd	COMDrawingEfficiency_s	0.4749	0.0620	**2.9×10^{-14}**
VRd	COMLongLatencyCount_s	0.4717	0.0620	**4.1×10^{-14}**
VRd	COMInformationProcessing	0.4614	0.0626	**2.4×10^{-13}**
VRd	COMDrawingProcessEfficiency_s	0.4550	0.0622	**3.7×10^{-13}**
VRd	COMRelativeLongLatency	-0.4567	0.0626	**4.2×10^{-13}**
VRd	COMLatencyVariability	-0.4553	0.0624	**4.3×10^{-13}**
VRd	COMInformationProcessing_s	0.4442	0.0618	**9.5×10^{-13}**
VRd	COPDrawingEfficiency	0.4536	0.0633	**1.0×10^{-12}**
VRd	COMAverageLatency	-0.4508	0.0634	**1.6×10^{-12}**
VRd	COPInformationProcessing	0.4510	0.0637	**2.0×10^{-12}**
VRd	COMLatencyVariability_s	0.4328	0.0617	**3.1×10^{-12}**
VRd	COMRelativeLongLatency_s	0.4282	0.0619	**6.2×10^{-12}**
VRd	COMLongestLatency	-0.4301	0.0622	**6.4×10^{-12}**
VRd	COMTotalTime	-0.4375	0.0634	**7.2×10^{-12}**
VRd	COMAverageLatency_s	0.4282	0.0623	**8.2×10^{-12}**
VRd	COPDrawingEfficiency_s	0.4303	0.0627	**8.6×10^{-12}**
VRd	COPDrawingProcessEfficiency	0.4255	0.0634	**2.5×10^{-11}**
VRd	COMLongestLatency_s	0.4073	0.0616	**4.8×10^{-11}**
VRd	COPTotalTime	-0.4289	0.0649	**4.8×10^{-11}**
VRd	COPRelativeLongLatency	-0.4229	0.0641	**5.4×10^{-11}**
VRd	COMTotalTime_s	0.4068	0.0623	**8.1×10^{-11}**
VRd	COPLatencyVariability	-0.4145	0.0636	**9.1×10^{-11}**
VRd	COPAverageLatency	-0.4156	0.0640	**1.1×10^{-10}**
VRd	COPInformationProcessing_s	0.4097	0.0633	**1.2×10^{-10}**
VRd	COPOscillatoryMotion_s	0.3943	0.0620	**2.4×10^{-10}**
VRd	COPDrawingProcessEfficiency_s	0.3993	0.0628	**2.5×10^{-10}**
VRd	COPTotalTime_s	0.3981	0.0631	**3.4×10^{-10}**
VRd	COPRelativeLongLatency_s	0.3995	0.0635	**3.7×10^{-10}**
VRd	COPLongestLatency	-0.3965	0.0634	**4.8×10^{-10}**
VRd	COPAverageLatency_s	0.3937	0.0631	**5.4×10^{-10}**
VRd	COMOscillatoryMotion	-0.4095	0.0658	**6.0×10^{-10}**
VRd	COPSimpleMotor	0.3894	0.0631	**8.1×10^{-10}**
VRd	COPLatencyVariability_s	0.3877	0.0630	**9.4×10^{-10}**
VRd	COMOscillatoryMotion_s	0.3916	0.0637	**9.5×10^{-10}**
VRd	COPLongLatencyCount_s	0.3839	0.0625	**9.8×10^{-10}**

VRd	COMClockfaceCircularity_s	0.3697	0.0616	**2.3×10^{-9}**
VRd	COMClockfaceCircularity	-0.3729	0.0622	**2.3×10^{-9}**
VRd	COPLongestLatency_s	0.3753	0.0628	**2.6×10^{-9}**
VRd	COPOscillatoryMotion	-0.3668	0.0639	**1.1×10^{-8}**
VRd	COPClockfaceCircularity_s	0.3528	0.0616	**1.2×10^{-8}**
VRd	COMLongLatencyCount	-0.3712	0.0651	**1.4×10^{-8}**
VRd	COPSimpleMotor_s	0.3505	0.0617	**1.5×10^{-8}**
VRd	COMSimpleMotor	0.3439	0.0636	**7.0×10^{-8}**
VRd	COPLongLatencyCount	-0.3438	0.0659	**2.0×10^{-7}**
VRd	COMSimpleMotor_s	0.3167	0.0626	**4.6×10^{-7}**
VRd	COPClockfaceCircularity	-0.2977	0.0618	**1.6×10^{-6}**
VRd	COMNoise_s	0.2792	0.0628	**9.1×10^{-6}**
VRd	COPVerticalSpatialPlacement_s	0.2343	0.0617	**1.5×10^{-4}**
VRd	COPDrawingSize_s	0.2293	0.0618	**2.1×10^{-4}**
VRd	COPDrawingSize	0.2214	0.0619	**3.5×10^{-4}**
VRd	COMPercentThinkTime	-0.2154	0.0626	5.8×10^{-4}
VRd	COMNoise	-0.2206	0.0644	6.3×10^{-4}
VRd	COMPercentInkTime	0.2124	0.0625	6.9×10^{-4}
VRd	COMDrawingSize_s	0.2120	0.0625	7.1×10^{-4}
VRd	COMDrawingSize	0.2119	0.0626	7.3×10^{-4}
VRd	COMInitiationSpeed	0.2068	0.0619	8.4×10^{-4}
VRd	COMPercentInkTime_s	-0.2056	0.0626	1.0×10^{-3}
VRd	COMHorizontalSpatialPlacement_s	0.2011	0.0616	1.1×10^{-3}
VRd	COMPercentThinkTime_s	0.2029	0.0625	1.2×10^{-3}
VRd	COPTerminationSpeed	0.1993	0.0623	1.4×10^{-3}
VRd	COPVerticalSpatialPlacement	-0.1976	0.0619	1.4×10^{-3}
VRd	COMInitiationSpeed_s	0.1942	0.0619	1.7×10^{-3}
VRd	COMHorizontalSpatialPlacement	-0.1932	0.0617	1.8×10^{-3}
VRd	COMTerminationSpeed	0.1862	0.0620	2.7×10^{-3}
VRd	COMAverageSpeed	0.1825	0.0620	3.3×10^{-3}
VRd	COPInitiationSpeed	0.1804	0.0619	3.6×10^{-3}
VRd	COPAverageSpeed	0.1792	0.0622	4.0×10^{-3}
VRd	COPTerminationSpeed_s	0.1783	0.0621	4.1×10^{-3}
VRd	COMVerticalSpatialPlacement_s	0.1729	0.0617	5.1×10^{-3}
VRd	COMTerminationSpeed_s	0.1702	0.0619	6.0×10^{-3}
VRd	COMAverageSpeed_s	0.1662	0.0620	7.4×10^{-3}
VRd	COPInitiationSpeed_s	0.1570	0.0617	1.1×10^{-2}
VRd	COMVerticalSpatialPlacement	-0.1562	0.0618	1.2×10^{-2}
VRd	COPPercentInkTime	0.1584	0.0632	1.2×10^{-2}
VRd	COPAverageSpeed_s	0.1531	0.0619	1.3×10^{-2}
VRd	COPPercentThinkTime_s	0.1496	0.0633	1.8×10^{-2}
VRd	COPPercentThinkTime	-0.1391	0.0634	2.8×10^{-2}
VRd	COPPercentInkTime_s	-0.1309	0.0635	3.9×10^{-2}

VRd	COMStrokeCountConformity_s	0.1179	0.0626	6.0×10^{-2}
VRd	COMStrokeCountConformity	-0.1115	0.0660	9.1×10^{-2}
VRd	COPStrokeCountConformity_s	0.1018	0.0626	1.0×10^{-1}
VRd	COMMaxSpeed	0.1006	0.0618	1.0×10^{-1}
VRd	COMInkLength	0.0966	0.0629	1.2×10^{-1}
VRd	COMInkLength_s	0.0960	0.0629	1.3×10^{-1}
VRd	COPMaxSpeed	0.0930	0.0620	1.3×10^{-1}
VRd	COPNoise_s	0.0935	0.0633	1.4×10^{-1}
VRd	COPHorizontalSpatialPlacement	0.0857	0.0620	1.7×10^{-1}
VRd	COMMaxSpeed_s	0.0838	0.0618	1.8×10^{-1}
VRd	COPNoise	-0.0733	0.0643	2.5×10^{-1}
VRd	COPMaxSpeed_s	0.0679	0.0618	2.7×10^{-1}
VRd	COPInkLength_s	0.0530	0.0623	4.0×10^{-1}
VRd	COPInkLength	0.0443	0.0628	4.8×10^{-1}
VRd	COPHorizontalSpatialPlacement_s	-0.0429	0.0621	4.9×10^{-1}
VRd	COPStrokeCountConformity	-0.0355	0.0687	6.1×10^{-1}
VRr	DCTScore	0.2542	0.0218	**2.1×10^{-30}**
VRr	COMComponentPlacement_s	0.2204	0.0212	**8.9×10^{-25}**
VRr	COMSpatialReasoning_s	0.2186	0.0210	**9.0×10^{-25}**
VRr	COMComponentPlacement	-0.2220	0.0216	**4.3×10^{-24}**
VRr	COMSpatialReasoning	0.2183	0.0214	**6.4×10^{-24}**
VRr	COMLongLatencyCount_s	0.1700	0.0211	**1.3×10^{-15}**
VRr	COMDrawingProcessEfficiency	0.1702	0.0215	**4.0×10^{-15}**
VRr	COPSpatialReasoning	0.1657	0.0213	**1.3×10^{-14}**
VRr	COMDrawingProcessEfficiency_s	0.1610	0.0212	**4.7×10^{-14}**
VRr	COPSpatialReasoning_s	0.1590	0.0211	**6.6×10^{-14}**
VRr	COMLongestLatency	-0.1575	0.0212	**1.5×10^{-13}**
VRr	COMInformationProcessing	0.1587	0.0213	**1.6×10^{-13}**
VRr	COMLatencyVariability	-0.1574	0.0213	**2.0×10^{-13}**
VRr	COMInformationProcessing_s	0.1546	0.0211	**3.2×10^{-13}**
VRr	COMDrawingEfficiency	0.1564	0.0214	**3.6×10^{-13}**
VRr	COMDrawingEfficiency_s	0.1521	0.0212	**9.5×10^{-13}**
VRr	COMLatencyVariability_s	0.1508	0.0210	**1.0×10^{-12}**
VRr	COMLongestLatency_s	0.1499	0.0210	**1.2×10^{-12}**
VRr	COMLongLatencyCount	-0.1564	0.0221	**2.0×10^{-12}**
VRr	COMTotalTime	-0.1526	0.0216	**2.2×10^{-12}**
VRr	COMRelativeLongLatency	-0.1508	0.0214	**2.3×10^{-12}**
VRr	COPComponentPlacement_s	0.1468	0.0211	**4.6×10^{-12}**
VRr	COPComponentPlacement	-0.1471	0.0215	**1.1×10^{-11}**
VRr	COMAverageLatency	-0.1476	0.0217	**1.2×10^{-11}**
VRr	COMTotalTime_s	0.1426	0.0212	**2.3×10^{-11}**
VRr	COMRelativeLongLatency_s	0.1410	0.0211	**3.4×10^{-11}**

VRr	COMAverageLatency_s	0.1401	0.0213	**5.5×10^{-11}**
VRr	COPDrawingProcessEfficiency	0.1382	0.0216	**1.8×10^{-10}**
VRr	COPTotalTime	-0.1396	0.0221	**3.0×10^{-10}**
VRr	COPDrawingEfficiency	0.1341	0.0216	**6.0×10^{-10}**
VRr	COPInformationProcessing	0.1341	0.0217	**8.1×10^{-10}**
VRr	COPDrawingProcessEfficiency_s	0.1306	0.0214	**1.1×10^{-9}**
VRr	COPTotalTime_s	0.1303	0.0214	**1.5×10^{-9}**
VRr	COPLongLatencyCount	-0.1353	0.0223	**1.7×10^{-9}**
VRr	COPDrawingEfficiency_s	0.1275	0.0213	**2.8×10^{-9}**
VRr	COMOscillatoryMotion	-0.1324	0.0225	**4.4×10^{-9}**
VRr	COPRelativeLongLatency	-0.1262	0.0218	**8.9×10^{-9}**
VRr	COPAverageLatency	-0.1248	0.0218	**1.2×10^{-8}**
VRr	COMOscillatoryMotion_s	0.1242	0.0217	**1.2×10^{-8}**
VRr	COPInformationProcessing_s	0.1212	0.0216	**2.1×10^{-8}**
VRr	COMSimpleMotor	0.1213	0.0216	**2.3×10^{-8}**
VRr	COPRelativeLongLatency_s	0.1190	0.0216	**4.1×10^{-8}**
VRr	COPAverageLatency_s	0.1176	0.0215	**5.0×10^{-8}**
VRr	COMSimpleMotor_s	0.1140	0.0213	**9.9×10^{-8}**
VRr	COPLongLatencyCount_s	0.1136	0.0213	**1.0×10^{-7}**
VRr	COPLatencyVariability	-0.1151	0.0217	**1.3×10^{-7}**
VRr	COPLatencyVariability_s	0.1069	0.0215	**7.5×10^{-7}**
VRr	COPLongestLatency	-0.1072	0.0216	**8.0×10^{-7}**
VRr	COPClockfaceCircularity_s	0.1004	0.0211	**2.0×10^{-6}**
VRr	COMClockfaceCircularity	-0.1009	0.0213	**2.3×10^{-6}**
VRr	COMClockfaceCircularity_s	0.0993	0.0211	**2.7×10^{-6}**
VRr	COPLongestLatency_s	0.1007	0.0214	**2.8×10^{-6}**
VRr	COPSimpleMotor	0.0963	0.0216	**8.7×10^{-6}**
VRr	COPOscillatoryMotion_s	0.0880	0.0213	**3.6×10^{-5}**
VRr	COMInitiationSpeed	0.0857	0.0210	**4.8×10^{-5}**
VRr	COPSimpleMotor_s	0.0858	0.0211	**5.0×10^{-5}**
VRr	COMVerticalSpatialPlacement_s	0.0814	0.0209	**1.0×10^{-4}**
VRr	COMInitiationSpeed_s	0.0818	0.0210	**1.0×10^{-4}**
VRr	COMAverageSpeed	0.0802	0.0211	**1.5×10^{-4}**
VRr	COPClockfaceCircularity	-0.0801	0.0211	**1.5×10^{-4}**
VRr	COMDrawingSize	0.0807	0.0213	**1.6×10^{-4}**
VRr	COMVerticalSpatialPlacement	-0.0789	0.0209	**1.7×10^{-4}**
VRr	COPVerticalSpatialPlacement_s	0.0786	0.0210	**1.9×10^{-4}**
VRr	COMDrawingSize_s	0.0787	0.0213	**2.2×10^{-4}**
VRr	COPDrawingSize_s	0.0769	0.0210	**2.6×10^{-4}**
VRr	COMTerminationSpeed	0.0754	0.0211	**3.5×10^{-4}**
VRr	COPOscillatoryMotion	-0.0780	0.0219	**3.8×10^{-4}**
VRr	COPDrawingSize	0.0749	0.0211	**3.8×10^{-4}**
VRr	COMAverageSpeed_s	0.0746	0.0211	**4.1×10^{-4}**

VRr	COMTerminationSpeed_s	0.0704	0.0210	8.3×10^{-4}
VRr	COPVerticalSpatialPlacement	-0.0698	0.0211	9.5×10^{-4}
VRr	COMPercentThinkTime	-0.0699	0.0213	1.1×10^{-3}
VRr	COMPercentInkTime	0.0681	0.0213	1.4×10^{-3}
VRr	COPInitiationSpeed	0.0662	0.0211	1.7×10^{-3}
VRr	COMPercentInkTime_s	-0.0654	0.0213	2.2×10^{-3}
VRr	COMNoise	-0.0667	0.0219	2.4×10^{-3}
VRr	COMPercentThinkTime_s	0.0637	0.0213	2.8×10^{-3}
VRr	COMNoise_s	0.0632	0.0215	3.3×10^{-3}
VRr	COPStrokeCountConformity	-0.0665	0.0233	4.4×10^{-3}
VRr	COPInitiationSpeed_s	0.0590	0.0210	5.0×10^{-3}
VRr	COPAverageSpeed	0.0581	0.0212	6.2×10^{-3}
VRr	COPTerminationSpeed	0.0538	0.0212	1.1×10^{-2}
VRr	COMStrokeCountConformity	-0.0561	0.0224	1.2×10^{-2}
VRr	COMMaxSpeed	0.0512	0.0210	1.5×10^{-2}
VRr	COPAverageSpeed_s	0.0493	0.0211	1.9×10^{-2}
VRr	COMMaxSpeed_s	0.0457	0.0210	3.0×10^{-2}
VRr	COPTerminationSpeed_s	0.0459	0.0211	3.0×10^{-2}
VRr	COPPercentInkTime	0.0447	0.0215	3.8×10^{-2}
VRr	COPMaxSpeed	0.0410	0.0211	5.2×10^{-2}
VRr	COMInkLength_s	0.0413	0.0214	5.4×10^{-2}
VRr	COPPercentThinkTime_s	0.0412	0.0216	5.6×10^{-2}
VRr	COPNoise	-0.0407	0.0219	6.3×10^{-2}
VRr	COMInkLength	0.0399	0.0214	6.3×10^{-2}
VRr	COPPercentThinkTime	-0.0383	0.0216	7.6×10^{-2}
VRr	COPPercentInkTime_s	-0.0347	0.0216	1.1×10^{-1}
VRr	COPMaxSpeed_s	0.0320	0.0210	1.3×10^{-1}
VRr	COPNoise_s	0.0305	0.0216	1.6×10^{-1}
VRr	COPHorizontalSpatialPlacement	0.0286	0.0212	1.8×10^{-1}
VRr	COPStrokeCountConformity_s	0.0279	0.0213	1.9×10^{-1}
VRr	COPHorizontalSpatialPlacement_s	-0.0167	0.0212	4.3×10^{-1}
VRr	COPInkLength_s	0.0133	0.0212	5.3×10^{-1}
VRr	COMStrokeCountConformity_s	0.0133	0.0213	5.3×10^{-1}
VRr	COMHorizontalSpatialPlacement_s	0.0114	0.0211	5.9×10^{-1}
VRr	COPInkLength	0.0107	0.0214	6.2×10^{-1}
VRr	COMHorizontalSpatialPlacement	-0.0100	0.0211	6.4×10^{-1}
PASi	COMComponentPlacement_s	0.6706	0.0741	**3.3×10^{-19}**
PASi	COMComponentPlacement	-0.6805	0.0758	**6.0×10^{-19}**
PASi	COMSpatialReasoning_s	0.6566	0.0735	**9.3×10^{-19}**
PASi	COMSpatialReasoning	0.6633	0.0748	**1.6×10^{-18}**
PASi	DCTScore	0.6029	0.0777	**1.3×10^{-14}**
PASi	COPComponentPlacement	-0.4085	0.0759	**8.2×10^{-8}**

PASi	COPComponentPlacement_s	0.3978	0.0744	**9.9×10^{-8}**
PASi	COPSpatialReasoning	0.3693	0.0755	**1.1×10^{-6}**
PASi	COMDrawingProcessEfficiency	0.3691	0.0761	**1.3×10^{-6}**
PASi	COPSpatialReasoning_s	0.3541	0.0744	**2.1×10^{-6}**
PASi	COMDrawingEfficiency	0.3593	0.0758	**2.3×10^{-6}**
PASi	COMLongLatencyCount	-0.3599	0.0781	**4.3×10^{-6}**
PASi	COMDrawingEfficiency_s	0.3434	0.0751	**5.1×10^{-6}**
PASi	COMDrawingProcessEfficiency_s	0.3426	0.0750	**5.2×10^{-6}**
PASi	COMLongLatencyCount_s	0.3370	0.0746	**6.6×10^{-6}**
PASi	COMRelativeLongLatency	-0.3190	0.0755	**2.5×10^{-5}**
PASi	COMLatencyVariability	-0.3108	0.0754	**3.9×10^{-5}**
PASi	COMInformationProcessing	0.3106	0.0757	**4.2×10^{-5}**
PASi	COPClockfaceCircularity_s	0.3015	0.0738	**4.6×10^{-5}**
PASi	COMClockfaceCircularity	-0.2959	0.0743	**7.0×10^{-5}**
PASi	COMClockfaceCircularity_s	0.2929	0.0737	**7.4×10^{-5}**
PASi	COMLongestLatency	-0.2972	0.0752	**8.0×10^{-5}**
PASi	COMAverageLatency	-0.2973	0.0764	**1.0×10^{-4}**
PASi	COMInformationProcessing_s	0.2882	0.0748	**1.2×10^{-4}**
PASi	COPClockfaceCircularity	-0.2808	0.0740	**1.5×10^{-4}**
PASi	COMRelativeLongLatency_s	0.2830	0.0747	**1.6×10^{-4}**
PASi	COMLatencyVariability_s	0.2793	0.0745	**1.8×10^{-4}**
PASi	COMLongestLatency_s	0.2705	0.0745	**2.9×10^{-4}**
PASi	COPLongLatencyCount	-0.2848	0.0786	**3.0×10^{-4}**
PASi	COMTotalTime	-0.2755	0.0765	**3.3×10^{-4}**
PASi	COPLongLatencyCount_s	0.2660	0.0747	**3.8×10^{-4}**
PASi	COMDrawingSize	0.2644	0.0752	**4.5×10^{-4}**
PASi	COMDrawingSize_s	0.2603	0.0750	5.3×10^{-4}
PASi	COMAverageLatency_s	0.2596	0.0751	5.6×10^{-4}
PASi	COPInformationProcessing	0.2592	0.0767	7.4×10^{-4}
PASi	COPRelativeLongLatency	-0.2486	0.0770	1.3×10^{-3}
PASi	COMTotalTime_s	0.2424	0.0751	1.3×10^{-3}
PASi	COPLatencyVariability	-0.2459	0.0765	1.3×10^{-3}
PASi	COPLongestLatency	-0.2448	0.0762	1.3×10^{-3}
PASi	COMVerticalSpatialPlacement_s	0.2270	0.0735	2.0×10^{-3}
PASi	COPInformationProcessing_s	0.2330	0.0759	2.2×10^{-3}
PASi	COPRelativeLongLatency_s	0.2315	0.0762	2.4×10^{-3}
PASi	COPLongestLatency_s	0.2236	0.0755	3.1×10^{-3}
PASi	COPLatencyVariability_s	0.2203	0.0758	3.7×10^{-3}
PASi	COMVerticalSpatialPlacement	-0.2127	0.0736	3.9×10^{-3}
PASi	COMOscillatoryMotion_s	0.2132	0.0766	5.4×10^{-3}
PASi	COMOscillatoryMotion	-0.2186	0.0793	5.9×10^{-3}
PASi	COMPercentThinkTime	-0.2058	0.0748	6.0×10^{-3}
PASi	COPTotalTime	-0.2119	0.0780	6.6×10^{-3}

PASi	COMPercentInkTime	0.1971	0.0747	8.4×10^{-3}
PASi	COMNoise_s	0.1959	0.0752	9.3×10^{-3}
PASi	COMPercentInkTime_s	-0.1925	0.0748	1.0×10^{-2}
PASi	COMNoise	-0.1961	0.0771	1.1×10^{-2}
PASi	COPTotalTime_s	0.1911	0.0757	1.2×10^{-2}
PASi	COPAverageLatency	-0.1924	0.0769	1.2×10^{-2}
PASi	COMPercentThinkTime_s	0.1841	0.0748	1.4×10^{-2}
PASi	COPAverageLatency_s	0.1790	0.0757	1.8×10^{-2}
PASi	COPDrawingEfficiency	0.1792	0.0761	1.9×10^{-2}
PASi	COPDrawingEfficiency_s	0.1678	0.0753	2.6×10^{-2}
PASi	COMInitiationSpeed	0.1646	0.0741	2.6×10^{-2}
PASi	COMSimpleMotor	0.1650	0.0763	3.1×10^{-2}
PASi	COPDrawingProcessEfficiency	0.1629	0.0761	3.2×10^{-2}
PASi	COMInkLength_s	0.1578	0.0755	3.7×10^{-2}
PASi	COMInkLength	0.1577	0.0755	3.7×10^{-2}
PASi	COMInitiationSpeed_s	0.1518	0.0741	4.0×10^{-2}
PASi	COPPercentInkTime	0.1493	0.0755	4.8×10^{-2}
PASi	COPPercentThinkTime_s	0.1455	0.0756	5.4×10^{-2}
PASi	COPDrawingProcessEfficiency_s	0.1418	0.0752	6.0×10^{-2}
PASi	COPOscillatoryMotion_s	0.1398	0.0747	6.1×10^{-2}
PASi	COMSimpleMotor_s	0.1406	0.0752	6.2×10^{-2}
PASi	COPOscillatoryMotion	-0.1405	0.0770	6.8×10^{-2}
PASi	COPDrawingSize_s	0.1301	0.0740	7.9×10^{-2}
PASi	COPPercentThinkTime	-0.1324	0.0757	8.0×10^{-2}
PASi	COPPercentInkTime_s	-0.1295	0.0758	8.8×10^{-2}
PASi	COPDrawingSize	0.1228	0.0741	9.8×10^{-2}
PASi	COPStrokeCountConformity	-0.1294	0.0814	1.1×10^{-1}
PASi	COMAverageSpeed	0.1069	0.0741	1.5×10^{-1}
PASi	COPSimpleMotor	0.1097	0.0762	1.5×10^{-1}
PASi	COPStrokeCountConformity_s	0.1001	0.0750	1.8×10^{-1}
PASi	COPHorizontalSpatialPlacement_s	0.0917	0.0741	2.2×10^{-1}
PASi	COPVerticalSpatialPlacement_s	0.0907	0.0740	2.2×10^{-1}
PASi	COMAverageSpeed_s	0.0890	0.0740	2.3×10^{-1}
PASi	COPHorizontalSpatialPlacement	-0.0887	0.0741	2.3×10^{-1}
PASi	COPVerticalSpatialPlacement	-0.0868	0.0741	2.4×10^{-1}
PASi	COMHorizontalSpatialPlacement_s	0.0846	0.0737	2.5×10^{-1}
PASi	COPInkLength	-0.0805	0.0750	2.8×10^{-1}
PASi	COMStrokeCountConformity	-0.0832	0.0784	2.9×10^{-1}
PASi	COMTerminationSpeed	0.0767	0.0742	3.0×10^{-1}
PASi	COPSimpleMotor_s	0.0767	0.0745	3.0×10^{-1}
PASi	COPInkLength_s	-0.0765	0.0745	3.0×10^{-1}
PASi	COMMaxSpeed	0.0716	0.0738	3.3×10^{-1}
PASi	COMHorizontalSpatialPlacement	-0.0708	0.0737	3.4×10^{-1}

PASi	COMTerminationSpeed_s	0.0590	0.0740	4.3×10^{-1}
PASi	COMMaxSpeed_s	0.0553	0.0737	4.5×10^{-1}
PASi	COPNoise	-0.0400	0.0767	6.0×10^{-1}
PASi	COMStrokeCountConformity_s	0.0381	0.0747	6.1×10^{-1}
PASi	COPNoise_s	0.0335	0.0757	6.6×10^{-1}
PASi	COPTerminationSpeed	0.0326	0.0747	6.6×10^{-1}
PASi	COPInitiationSpeed	0.0249	0.0746	7.4×10^{-1}
PASi	COPAverageSpeed_s	-0.0205	0.0742	7.8×10^{-1}
PASi	COPMaxSpeed_s	-0.0176	0.0738	8.1×10^{-1}
PASi	COPMaxSpeed	0.0103	0.0741	8.9×10^{-1}
PASi	COPTerminationSpeed_s	0.0096	0.0744	9.0×10^{-1}
PASi	COPAverageSpeed	0.0060	0.0746	9.4×10^{-1}
PASi	COPInitiationSpeed_s	-0.0005	0.0743	9.9×10^{-1}
PASd	DCTScore	0.2601	0.0310	**9.3×10^{-17}**
PASd	COMSpatialReasoning_s	0.2557	0.0297	**1.3×10^{-17}**
PASd	COMComponentPlacement_s	0.2575	0.0299	**1.4×10^{-17}**
PASd	COMComponentPlacement	-0.2626	0.0305	**1.5×10^{-17}**
PASd	COMSpatialReasoning	0.2577	0.0301	**2.4×10^{-17}**
PASd	COMDrawingProcessEfficiency	0.2020	0.0303	**3.2×10^{-11}**
PASd	COMDrawingEfficiency	0.1981	0.0301	**5.7×10^{-11}**
PASd	COMDrawingEfficiency_s	0.1914	0.0298	**1.7×10^{-10}**
PASd	COMDrawingProcessEfficiency_s	0.1878	0.0298	**3.8×10^{-10}**
PASd	COMLongLatencyCount	-0.1943	0.0313	**6.1×10^{-10}**
PASd	COMLongLatencyCount_s	0.1771	0.0300	**4.0×10^{-9}**
PASd	COMLatencyVariability	-0.1701	0.0300	**1.6×10^{-8}**
PASd	COPComponentPlacement_s	0.1685	0.0297	**1.6×10^{-8}**
PASd	COMInformationProcessing	0.1702	0.0301	**1.8×10^{-8}**
PASd	COPComponentPlacement	-0.1706	0.0303	**2.1×10^{-8}**
PASd	COPSpatialReasoning	0.1693	0.0302	**2.3×10^{-8}**
PASd	COMLongestLatency	-0.1637	0.0299	**4.8×10^{-8}**
PASd	COMRelativeLongLatency	-0.1647	0.0301	**4.9×10^{-8}**
PASd	COMInformationProcessing_s	0.1616	0.0297	**6.1×10^{-8}**
PASd	COPSpatialReasoning_s	0.1610	0.0298	**7.1×10^{-8}**
PASd	COMLatencyVariability_s	0.1579	0.0296	**1.1×10^{-7}**
PASd	COMAverageLatency	-0.1577	0.0305	**2.5×10^{-7}**
PASd	COMLongestLatency_s	0.1527	0.0296	**2.7×10^{-7}**
PASd	COMRelativeLongLatency_s	0.1512	0.0298	**4.2×10^{-7}**
PASd	COPClockfaceCircularity_s	0.1496	0.0295	**4.3×10^{-7}**
PASd	COMAverageLatency_s	0.1443	0.0299	**1.5×10^{-6}**
PASd	COPLongLatencyCount	-0.1462	0.0315	**3.8×10^{-6}**
PASd	COPLongLatencyCount_s	0.1391	0.0300	**3.9×10^{-6}**
PASd	COMTotalTime	-0.1392	0.0305	**5.5×10^{-6}**

PASd	COPClockfaceCircularity	-0.1345	0.0296	**5.8×10^{-6}**
PASd	COPInformationProcessing	0.1386	0.0307	**6.5×10^{-6}**
PASd	COMTotalTime_s	0.1281	0.0300	**2.0×10^{-5}**
PASd	COPInformationProcessing_s	0.1287	0.0304	**2.4×10^{-5}**
PASd	COPRelativeLongLatency_s	0.1258	0.0305	**3.8×10^{-5}**
PASd	COPRelativeLongLatency	-0.1267	0.0308	**4.1×10^{-5}**
PASd	COPLongestLatency	-0.1248	0.0304	**4.3×10^{-5}**
PASd	COPLatencyVariability	-0.1234	0.0306	**5.6×10^{-5}**
PASd	COPLongestLatency_s	0.1215	0.0301	**5.7×10^{-5}**
PASd	COPTotalTime_s	0.1215	0.0303	**6.4×10^{-5}**
PASd	COPDrawingProcessEfficiency	0.1190	0.0305	**9.9×10^{-5}**
PASd	COPTotalTime	-0.1217	0.0312	**1.0×10^{-4}**
PASd	COPLatencyVariability_s	0.1175	0.0303	**1.1×10^{-4}**
PASd	COMDrawingSize	0.1143	0.0299	**1.4×10^{-4}**
PASd	COPAverageLatency_s	0.1153	0.0303	**1.5×10^{-4}**
PASd	COMDrawingSize_s	0.1124	0.0299	**1.7×10^{-4}**
PASd	COPAverageLatency	-0.1153	0.0308	**1.9×10^{-4}**
PASd	COPDrawingProcessEfficiency_s	0.1130	0.0302	**1.9×10^{-4}**
PASd	COPDrawingEfficiency_s	0.1123	0.0302	**2.0×10^{-4}**
PASd	COPDrawingEfficiency	0.1133	0.0305	**2.1×10^{-4}**
PASd	COMPercentThinkTime	-0.1067	0.0299	**3.7×10^{-4}**
PASd	COMPercentInkTime	0.1046	0.0299	4.8×10^{-4}
PASd	COMInitiationSpeed	0.1034	0.0298	5.3×10^{-4}
PASd	COMPercentInkTime_s	-0.1018	0.0299	6.8×10^{-4}
PASd	COMVerticalSpatialPlacement	-0.0998	0.0295	7.2×10^{-4}
PASd	COMNoise	-0.1043	0.0309	7.4×10^{-4}
PASd	COMVerticalSpatialPlacement_s	0.0983	0.0294	8.3×10^{-4}
PASd	COMPercentThinkTime_s	0.0997	0.0299	8.8×10^{-4}
PASd	COMInitiationSpeed_s	0.0985	0.0298	9.6×10^{-4}
PASd	COMInkLength_s	0.0959	0.0301	1.5×10^{-3}
PASd	COMInkLength	0.0953	0.0301	1.6×10^{-3}
PASd	COMClockfaceCircularity	-0.0935	0.0298	1.8×10^{-3}
PASd	COMClockfaceCircularity_s	0.0888	0.0296	2.7×10^{-3}
PASd	COMNoise_s	0.0788	0.0302	9.1×10^{-3}
PASd	COMOscillatoryMotion	-0.0794	0.0318	1.3×10^{-2}
PASd	COPPercentInkTime	0.0737	0.0302	1.5×10^{-2}
PASd	COPPercentThinkTime_s	0.0730	0.0303	1.6×10^{-2}
PASd	COMSimpleMotor	0.0718	0.0306	1.9×10^{-2}
PASd	COPSimpleMotor	0.0715	0.0305	1.9×10^{-2}
PASd	COMOscillatoryMotion_s	0.0706	0.0307	2.2×10^{-2}
PASd	COMAverageSpeed	0.0682	0.0298	2.2×10^{-2}
PASd	COMStrokeCountConformity	-0.0693	0.0315	2.8×10^{-2}
PASd	COPOscillatoryMotion_s	0.0643	0.0300	3.2×10^{-2}

PASd	COPPercentThinkTime	-0.0630	0.0303	3.8×10^{-2}
PASd	COPPercentInkTime_s	-0.0627	0.0303	3.9×10^{-2}
PASd	COMAverageSpeed_s	0.0614	0.0298	3.9×10^{-2}
PASd	COMSimpleMotor_s	0.0618	0.0301	4.1×10^{-2}
PASd	COPInitiationSpeed	0.0609	0.0298	4.1×10^{-2}
PASd	COMMaxSpeed	0.0595	0.0296	4.5×10^{-2}
PASd	COPSimpleMotor_s	0.0583	0.0298	5.1×10^{-2}
PASd	COPDrawingSize_s	0.0562	0.0296	5.8×10^{-2}
PASd	COPOscillatoryMotion	-0.0584	0.0309	5.9×10^{-2}
PASd	COPDrawingSize	0.0541	0.0297	6.8×10^{-2}
PASd	COPVerticalSpatialPlacement_s	0.0539	0.0297	7.0×10^{-2}
PASd	COMMaxSpeed_s	0.0525	0.0296	7.6×10^{-2}
PASd	COPInitiationSpeed_s	0.0492	0.0297	9.8×10^{-2}
PASd	COPVerticalSpatialPlacement	-0.0463	0.0298	1.2×10^{-1}
PASd	COPStrokeCountConformity	-0.0509	0.0328	1.2×10^{-1}
PASd	COPAverageSpeed	0.0438	0.0299	1.4×10^{-1}
PASd	COPMaxSpeed	0.0432	0.0297	1.5×10^{-1}
PASd	COMTerminationSpeed	0.0426	0.0298	1.5×10^{-1}
PASd	COPNoise	-0.0428	0.0308	1.6×10^{-1}
PASd	COMHorizontalSpatialPlacement	-0.0369	0.0295	2.1×10^{-1}
PASd	COMTerminationSpeed_s	0.0353	0.0297	2.4×10^{-1}
PASd	COMHorizontalSpatialPlacement_s	0.0339	0.0295	2.5×10^{-1}
PASd	COPTerminationSpeed	0.0341	0.0299	2.5×10^{-1}
PASd	COPAverageSpeed_s	0.0330	0.0298	2.7×10^{-1}
PASd	COPMaxSpeed_s	0.0314	0.0296	2.9×10^{-1}
PASd	COPStrokeCountConformity_s	0.0294	0.0302	3.3×10^{-1}
PASd	COPNoise_s	0.0272	0.0305	3.7×10^{-1}
PASd	COPTerminationSpeed_s	0.0250	0.0298	4.0×10^{-1}
PASd	COMStrokeCountConformity_s	0.0138	0.0301	6.5×10^{-1}
PASd	COPHorizontalSpatialPlacement	0.0104	0.0296	7.2×10^{-1}
PASd	COPHorizontalSpatialPlacement_s	-0.0029	0.0296	9.2×10^{-1}
PASd	COPInkLength	0.0020	0.0300	9.5×10^{-1}
PASd	COPInkLength_s	0.0019	0.0298	9.5×10^{-1}
PASr	DCTScore	0.1509	0.0367	**4.1×10^{-5}**
PASr	COPLongLatencyCount_s	0.1474	0.0350	**2.6×10^{-5}**
PASr	COPRelativeLongLatency	-0.1457	0.0359	**5.1×10^{-5}**
PASr	COPComponentPlacement	-0.1379	0.0355	**1.1×10^{-4}**
PASr	COPComponentPlacement_s	0.1351	0.0348	**1.1×10^{-4}**
PASr	COPLatencyVariability	-0.1370	0.0357	**1.3×10^{-4}**
PASr	COPInformationProcessing	0.1338	0.0358	**1.9×10^{-4}**
PASr	COMComponentPlacement_s	0.1316	0.0354	**2.0×10^{-4}**
PASr	COPRelativeLongLatency_s	0.1301	0.0355	**2.6×10^{-4}**

PASr	COMLongLatencyCount_s	0.1274	0.0350	**2.8×10^{-4}**
PASr	COPLongLatencyCount	-0.1333	0.0368	**3.0×10^{-4}**
PASr	COPInformationProcessing_s	0.1260	0.0355	**3.9×10^{-4}**
PASr	COPLongestLatency	-0.1255	0.0356	**4.2×10^{-4}**
PASr	COMComponentPlacement	-0.1251	0.0361	5.4×10^{-4}
PASr	COMSpatialReasoning_s	0.1211	0.0351	5.8×10^{-4}
PASr	COPAverageLatency	-0.1224	0.0359	6.7×10^{-4}
PASr	COPLatencyVariability_s	0.1171	0.0354	9.5×10^{-4}
PASr	COPTotalTime	-0.1202	0.0364	9.8×10^{-4}
PASr	COMSpatialReasoning	0.1162	0.0357	1.2×10^{-3}
PASr	COMAverageLatency	-0.1161	0.0357	1.2×10^{-3}
PASr	COMAverageLatency_s	0.1119	0.0350	1.4×10^{-3}
PASr	COMRelativeLongLatency	-0.1119	0.0353	1.5×10^{-3}
PASr	COMTotalTime	-0.1098	0.0357	2.1×10^{-3}
PASr	COPAverageLatency_s	0.1081	0.0354	2.3×10^{-3}
PASr	COPLongestLatency_s	0.1067	0.0352	2.5×10^{-3}
PASr	COPTotalTime_s	0.1064	0.0354	2.7×10^{-3}
PASr	COMRelativeLongLatency_s	0.1045	0.0349	2.8×10^{-3}
PASr	COMTotalTime_s	0.1041	0.0350	3.0×10^{-3}
PASr	COMInformationProcessing	0.1032	0.0353	3.5×10^{-3}
PASr	COMInformationProcessing_s	0.1010	0.0349	3.8×10^{-3}
PASr	COMDrawingProcessEfficiency	0.1026	0.0357	4.0×10^{-3}
PASr	COMDrawingEfficiency_s	0.0987	0.0351	4.9×10^{-3}
PASr	COMDrawingEfficiency	0.0980	0.0354	5.7×10^{-3}
PASr	COPSimpleMotor	0.0983	0.0356	5.8×10^{-3}
PASr	COPOscillatoryMotion	-0.0993	0.0360	5.9×10^{-3}
PASr	COMLatencyVariability	-0.0959	0.0352	6.5×10^{-3}
PASr	COMDrawingProcessEfficiency_s	0.0951	0.0351	6.8×10^{-3}
PASr	COPOscillatoryMotion_s	0.0947	0.0350	6.9×10^{-3}
PASr	COPSpatialReasoning	0.0957	0.0354	7.0×10^{-3}
PASr	COPSimpleMotor_s	0.0938	0.0348	7.0×10^{-3}
PASr	COMLatencyVariability_s	0.0902	0.0348	9.5×10^{-3}
PASr	COPDrawingProcessEfficiency	0.0923	0.0356	9.6×10^{-3}
PASr	COPSpatialReasoning_s	0.0903	0.0349	9.8×10^{-3}
PASr	COPDrawingProcessEfficiency_s	0.0878	0.0352	1.3×10^{-2}
PASr	COMPercentThinkTime	-0.0871	0.0350	1.3×10^{-2}
PASr	COMPercentInkTime	0.0859	0.0350	1.4×10^{-2}
PASr	COMPercentInkTime_s	-0.0841	0.0350	1.6×10^{-2}
PASr	COMPercentThinkTime_s	0.0827	0.0350	1.8×10^{-2}
PASr	COMLongLatencyCount	-0.0846	0.0366	2.1×10^{-2}
PASr	COMLongestLatency	-0.0787	0.0351	2.5×10^{-2}
PASr	COMLongestLatency_s	0.0743	0.0347	3.2×10^{-2}
PASr	COMOscillatoryMotion	-0.0784	0.0372	3.5×10^{-2}

PASr	COMSimpleMotor	0.0733	0.0358	4.1×10^{-2}
PASr	COPPercentThinkTime	-0.0701	0.0353	4.7×10^{-2}
PASr	COMOscillatoryMotion_s	0.0708	0.0360	4.9×10^{-2}
PASr	COPDrawingEfficiency_s	0.0673	0.0352	5.6×10^{-2}
PASr	COMInitiationSpeed	0.0662	0.0347	5.6×10^{-2}
PASr	COPDrawingEfficiency	0.0680	0.0356	5.6×10^{-2}
PASr	COMSimpleMotor_s	0.0663	0.0352	6.0×10^{-2}
PASr	COMNoise_s	0.0658	0.0352	6.2×10^{-2}
PASr	COMInitiationSpeed_s	0.0639	0.0346	6.5×10^{-2}
PASr	COPPercentInkTime	0.0647	0.0352	6.6×10^{-2}
PASr	COPPercentInkTime_s	-0.0635	0.0354	7.3×10^{-2}
PASr	COPStrokeCountConformity	-0.0672	0.0380	7.7×10^{-2}
PASr	COPTerminationSpeed	0.0614	0.0349	7.8×10^{-2}
PASr	COMNoise	-0.0625	0.0360	8.3×10^{-2}
PASr	COPAverageSpeed	0.0584	0.0348	9.3×10^{-2}
PASr	COMHorizontalSpatialPlacement_s	0.0577	0.0345	9.5×10^{-2}
PASr	COPPercentThinkTime_s	0.0580	0.0353	1.0×10^{-1}
PASr	COMHorizontalSpatialPlacement	-0.0566	0.0345	1.0×10^{-1}
PASr	COPTerminationSpeed_s	0.0569	0.0347	1.0×10^{-1}
PASr	COMAverageSpeed	0.0558	0.0347	1.1×10^{-1}
PASr	COPNoise	-0.0556	0.0359	1.2×10^{-1}
PASr	COPAverageSpeed_s	0.0533	0.0346	1.2×10^{-1}
PASr	COMAverageSpeed_s	0.0527	0.0347	1.3×10^{-1}
PASr	COPNoise_s	0.0529	0.0353	1.3×10^{-1}
PASr	COPMaxSpeed	0.0484	0.0346	1.6×10^{-1}
PASr	COPMaxSpeed_s	0.0432	0.0345	2.1×10^{-1}
PASr	COMTerminationSpeed	0.0434	0.0347	2.1×10^{-1}
PASr	COMStrokeCountConformity_s	0.0408	0.0350	2.4×10^{-1}
PASr	COPStrokeCountConformity_s	0.0405	0.0349	2.5×10^{-1}
PASr	COMTerminationSpeed_s	0.0395	0.0347	2.5×10^{-1}
PASr	COMMaxSpeed	0.0391	0.0346	2.6×10^{-1}
PASr	COMClockfaceCircularity_s	0.0372	0.0347	2.8×10^{-1}
PASr	COMClockfaceCircularity	-0.0359	0.0350	3.0×10^{-1}
PASr	COMMaxSpeed_s	0.0354	0.0345	3.1×10^{-1}
PASr	COPInitiationSpeed	0.0302	0.0347	3.8×10^{-1}
PASr	COPInkLength	-0.0277	0.0351	4.3×10^{-1}
PASr	COMVerticalSpatialPlacement_s	0.0269	0.0344	4.3×10^{-1}
PASr	COPInkLength_s	-0.0263	0.0348	4.5×10^{-1}
PASr	COMVerticalSpatialPlacement	-0.0252	0.0345	4.6×10^{-1}
PASr	COPInitiationSpeed_s	0.0244	0.0346	4.8×10^{-1}
PASr	COMInkLength_s	-0.0154	0.0352	6.6×10^{-1}
PASr	COMInkLength	-0.0135	0.0352	7.0×10^{-1}
PASr	COMDrawingSize_s	-0.0113	0.0350	7.5×10^{-1}

PASr	COPVerticalSpatialPlacement	-0.0094	0.0347	7.9×10^{-1}
PASr	COMDrawingSize	-0.0090	0.0350	8.0×10^{-1}
PASr	COPClockfaceCircularity	0.0089	0.0348	8.0×10^{-1}
PASr	COPClockfaceCircularity_s	-0.0060	0.0347	8.6×10^{-1}
PASr	COPDrawingSize_s	0.0055	0.0347	8.7×10^{-1}
PASr	COPVerticalSpatialPlacement_s	0.0054	0.0347	8.8×10^{-1}
PASr	COPDrawingSize	0.0047	0.0347	8.9×10^{-1}
PASr	COPHorizontalSpatialPlacement_s	0.0025	0.0347	9.4×10^{-1}
PASr	COMStrokeCountConformity	-0.0020	0.0366	9.6×10^{-1}
PASr	COPHorizontalSpatialPlacement	-0.0003	0.0346	9.9×10^{-1}
DSf	COMDrawingEfficiency	0.2030	0.0286	**1.8×10^{-12}**
DSf	COMDrawingProcessEfficiency	0.2020	0.0288	**3.4×10^{-12}**
DSf	COMDrawingEfficiency_s	0.1983	0.0284	**3.6×10^{-12}**
DSf	COMDrawingProcessEfficiency_s	0.1966	0.0284	**5.9×10^{-12}**
DSf	COMLongLatencyCount	-0.1966	0.0296	**4.2×10^{-11}**
DSf	COMComponentPlacement	-0.1893	0.0293	**1.3×10^{-10}**
DSf	COMComponentPlacement_s	0.1806	0.0287	**3.8×10^{-10}**
DSf	DCTScore	0.1675	0.0299	**2.5×10^{-8}**
DSf	COMSpatialReasoning	0.1613	0.0290	**3.1×10^{-8}**
DSf	COMSpatialReasoning_s	0.1577	0.0285	**3.7×10^{-8}**
DSf	COMInformationProcessing	0.1555	0.0287	**6.7×10^{-8}**
DSf	COMLongLatencyCount_s	0.1505	0.0285	**1.4×10^{-7}**
DSf	COMInformationProcessing_s	0.1494	0.0284	**1.5×10^{-7}**
DSf	COMRelativeLongLatency	-0.1473	0.0287	**3.1×10^{-7}**
DSf	COMLatencyVariability	-0.1467	0.0286	**3.3×10^{-7}**
DSf	COMLatencyVariability_s	0.1443	0.0283	**3.6×10^{-7}**
DSf	COMRelativeLongLatency_s	0.1447	0.0284	**3.7×10^{-7}**
DSf	COMLongestLatency	-0.1441	0.0285	**4.7×10^{-7}**
DSf	COMLongestLatency_s	0.1417	0.0282	**5.5×10^{-7}**
DSf	COMDrawingSize	0.1327	0.0285	**3.4×10^{-6}**
DSf	COMDrawingSize_s	0.1313	0.0284	**4.1×10^{-6}**
DSf	COMTotalTime	-0.1310	0.0291	**7.1×10^{-6}**
DSf	COPDrawingEfficiency	0.1305	0.0290	**7.2×10^{-6}**
DSf	COMAverageLatency	-0.1303	0.0291	**7.9×10^{-6}**
DSf	COMTotalTime_s	0.1265	0.0285	**9.7×10^{-6}**
DSf	COMAverageLatency_s	0.1263	0.0286	**1.0×10^{-5}**
DSf	COPStrokeCountConformity	-0.1337	0.0310	**1.7×10^{-5}**
DSf	COPDrawingEfficiency_s	0.1234	0.0287	**1.8×10^{-5}**
DSf	COPClockfaceCircularity_s	0.1181	0.0284	**3.3×10^{-5}**
DSf	COPClockfaceCircularity	-0.1118	0.0284	**8.8×10^{-5}**
DSf	COMClockfaceCircularity	-0.1095	0.0285	**1.3×10^{-4}**
DSf	COPDrawingProcessEfficiency	0.1086	0.0291	**1.9×10^{-4}**

DSf	COPDrawingProcessEfficiency_s	0.1003	0.0288	5.0×10^{-4}
DSf	COMClockfaceCircularity_s	0.0978	0.0283	5.6×10^{-4}
DSf	COPTotalTime	-0.1012	0.0297	6.8×10^{-4}
DSf	COPDrawingSize_s	0.0942	0.0283	8.9×10^{-4}
DSf	COPDrawingSize	0.0931	0.0283	1.0×10^{-3}
DSf	COPNoise	-0.0959	0.0293	1.1×10^{-3}
DSf	COMInkLength_s	0.0895	0.0287	1.9×10^{-3}
DSf	COPComponentPlacement_s	0.0883	0.0285	1.9×10^{-3}
DSf	COMInkLength	0.0885	0.0287	2.1×10^{-3}
DSf	COPComponentPlacement	-0.0885	0.0290	2.3×10^{-3}
DSf	COPTotalTime_s	0.0878	0.0289	2.4×10^{-3}
DSf	COPLongLatencyCount	-0.0894	0.0301	3.0×10^{-3}
DSf	COMStrokeCountConformity	-0.0874	0.0299	3.5×10^{-3}
DSf	COMNoise	-0.0854	0.0294	3.7×10^{-3}
DSf	COPRelativeLongLatency	-0.0841	0.0294	4.3×10^{-3}
DSf	COPRelativeLongLatency_s	0.0791	0.0291	6.6×10^{-3}
DSf	COPInformationProcessing	0.0777	0.0293	8.0×10^{-3}
DSf	COMPercentInkTime	0.0758	0.0286	8.1×10^{-3}
DSf	COMPercentThinkTime_s	0.0741	0.0286	9.6×10^{-3}
DSf	COMPercentThinkTime	-0.0733	0.0286	1.0×10^{-2}
DSf	COPLongestLatency	-0.0729	0.0291	1.2×10^{-2}
DSf	COPInformationProcessing_s	0.0725	0.0290	1.3×10^{-2}
DSf	COMPercentInkTime_s	-0.0715	0.0286	1.3×10^{-2}
DSf	COPAverageLatency	-0.0732	0.0294	1.3×10^{-2}
DSf	COPLongestLatency_s	0.0712	0.0288	1.3×10^{-2}
DSf	COPSpatialReasoning	0.0668	0.0290	2.1×10^{-2}
DSf	COPLatencyVariability	-0.0670	0.0292	2.2×10^{-2}
DSf	COMSimpleMotor	0.0668	0.0292	2.2×10^{-2}
DSf	COPLatencyVariability_s	0.0654	0.0289	2.4×10^{-2}
DSf	COPAverageLatency_s	0.0653	0.0290	2.4×10^{-2}
DSf	COPSpatialReasoning_s	0.0639	0.0286	2.5×10^{-2}
DSf	COMSimpleMotor_s	0.0629	0.0288	2.9×10^{-2}
DSf	COMNoise_s	0.0611	0.0288	3.4×10^{-2}
DSf	COMMaxSpeed	0.0587	0.0282	3.7×10^{-2}
DSf	COMMaxSpeed_s	0.0554	0.0282	4.9×10^{-2}
DSf	COMOscillatoryMotion	-0.0588	0.0304	5.3×10^{-2}
DSf	COPNoise_s	0.0551	0.0289	5.7×10^{-2}
DSf	COPLongLatencyCount_s	0.0537	0.0287	6.2×10^{-2}
DSf	COMOscillatoryMotion_s	0.0514	0.0294	8.1×10^{-2}
DSf	COMTerminationSpeed	0.0444	0.0284	1.2×10^{-1}
DSf	COPPercentInkTime	0.0450	0.0288	1.2×10^{-1}
DSf	COMInitiationSpeed	0.0430	0.0283	1.3×10^{-1}
DSf	COPStrokeCountConformity_s	0.0428	0.0286	1.3×10^{-1}

DSf	COPPercentThinkTime	-0.0430	0.0289	1.4×10^{-1}
DSf	COPPercentThinkTime_s	0.0425	0.0289	1.4×10^{-1}
DSf	COMTerminationSpeed_s	0.0413	0.0283	1.4×10^{-1}
DSf	COMAverageSpeed	0.0407	0.0284	1.5×10^{-1}
DSf	COPPercentInkTime_s	-0.0405	0.0290	1.6×10^{-1}
DSf	COMInitiationSpeed_s	0.0396	0.0283	1.6×10^{-1}
DSf	COMAverageSpeed_s	0.0371	0.0283	1.9×10^{-1}
DSf	COPInitiationSpeed	0.0330	0.0284	2.5×10^{-1}
DSf	COPMaxSpeed	0.0321	0.0283	2.6×10^{-1}
DSf	COPSimpleMotor	0.0330	0.0291	2.6×10^{-1}
DSf	COPAverageSpeed	0.0317	0.0285	2.6×10^{-1}
DSf	COPInkLength	0.0315	0.0287	2.7×10^{-1}
DSf	COPTerminationSpeed	0.0312	0.0285	2.7×10^{-1}
DSf	COPInitiationSpeed_s	0.0286	0.0283	3.1×10^{-1}
DSf	COPMaxSpeed_s	0.0279	0.0282	3.2×10^{-1}
DSf	COPInkLength_s	0.0281	0.0285	3.2×10^{-1}
DSf	COPSimpleMotor_s	0.0268	0.0284	3.5×10^{-1}
DSf	COPTerminationSpeed_s	0.0260	0.0284	3.6×10^{-1}
DSf	COPAverageSpeed_s	0.0254	0.0283	3.7×10^{-1}
DSf	COMHorizontalSpatialPlacement	-0.0251	0.0282	3.7×10^{-1}
DSf	COMStrokeCountConformity_s	0.0242	0.0286	4.0×10^{-1}
DSf	COPVerticalSpatialPlacement	0.0221	0.0284	4.4×10^{-1}
DSf	COPVerticalSpatialPlacement_s	-0.0198	0.0283	4.8×10^{-1}
DSf	COPHorizontalSpatialPlacement	0.0174	0.0283	5.4×10^{-1}
DSf	COMHorizontalSpatialPlacement_s	0.0153	0.0282	5.9×10^{-1}
DSf	COMVerticalSpatialPlacement_s	-0.0149	0.0282	6.0×10^{-1}
DSf	COPHorizontalSpatialPlacement_s	-0.0138	0.0284	6.3×10^{-1}
DSf	COPOscillatoryMotion_s	0.0070	0.0287	8.1×10^{-1}
DSf	COPOscillatoryMotion	-0.0066	0.0295	8.2×10^{-1}
DSf	COMVerticalSpatialPlacement	0.0050	0.0282	8.6×10^{-1}
DSb	COMComponentPlacement	-0.2302	0.0282	$\mathbf{6.3\times10^{-16}}$
DSb	COMComponentPlacement_s	0.2213	0.0277	$\mathbf{2.3\times10^{-15}}$
DSb	COMSpatialReasoning	0.2180	0.0280	$\mathbf{1.0\times10^{-14}}$
DSb	COMSpatialReasoning_s	0.2117	0.0275	$\mathbf{2.3\times10^{-14}}$
DSb	DCTScore	0.1917	0.0290	$\mathbf{4.5\times10^{-11}}$
DSb	COMDrawingProcessEfficiency	0.1813	0.0281	$\mathbf{1.3\times10^{-10}}$
DSb	COMDrawingEfficiency	0.1783	0.0279	$\mathbf{2.0\times10^{-10}}$
DSb	COMDrawingProcessEfficiency_s	0.1747	0.0277	$\mathbf{3.3\times10^{-10}}$
DSb	COMDrawingEfficiency_s	0.1738	0.0276	$\mathbf{3.9\times10^{-10}}$
DSb	COPClockfaceCircularity_s	0.1506	0.0273	$\mathbf{4.0\times10^{-8}}$
DSb	COPClockfaceCircularity	-0.1356	0.0274	$\mathbf{8.3\times10^{-7}}$
DSb	COMInformationProcessing	0.1378	0.0280	$\mathbf{9.4\times10^{-7}}$

DSb	COMAverageLatency_s	0.1350	0.0278	**1.3×10^{-6}**
DSb	COMAverageLatency	-0.1370	0.0283	**1.4×10^{-6}**
DSb	COMInformationProcessing_s	0.1332	0.0277	**1.6×10^{-6}**
DSb	COPComponentPlacement	-0.1342	0.0281	**2.0×10^{-6}**
DSb	COPComponentPlacement_s	0.1299	0.0276	**2.6×10^{-6}**
DSb	COMRelativeLongLatency	-0.1276	0.0280	**5.7×10^{-6}**
DSb	COMRelativeLongLatency_s	0.1260	0.0277	**5.8×10^{-6}**
DSb	COMLatencyVariability_s	0.1251	0.0276	**6.1×10^{-6}**
DSb	COMLatencyVariability	-0.1259	0.0279	**6.9×10^{-6}**
DSb	COMLongLatencyCount	-0.1254	0.0290	**1.7×10^{-5}**
DSb	COMLongestLatency	-0.1191	0.0278	**1.9×10^{-5}**
DSb	COMLongestLatency_s	0.1175	0.0275	**2.0×10^{-5}**
DSb	COMDrawingSize	0.1123	0.0277	**5.2×10^{-5}**
DSb	COMDrawingSize_s	0.1118	0.0277	**5.5×10^{-5}**
DSb	COMLongLatencyCount_s	0.1117	0.0280	**6.7×10^{-5}**
DSb	COMClockfaceCircularity_s	0.1068	0.0274	**1.0×10^{-4}**
DSb	COMTotalTime	-0.1106	0.0284	**1.0×10^{-4}**
DSb	COMTotalTime_s	0.1073	0.0279	**1.2×10^{-4}**
DSb	COMPercentInkTime	0.1026	0.0277	**2.2×10^{-4}**
DSb	COMPercentThinkTime_s	0.1022	0.0277	**2.4×10^{-4}**
DSb	COMClockfaceCircularity	-0.1016	0.0277	**2.5×10^{-4}**
DSb	COMPercentThinkTime	-0.0987	0.0278	**3.9×10^{-4}**
DSb	COMPercentInkTime_s	-0.0983	0.0278	**4.1×10^{-4}**
DSb	COMInkLength_s	0.0944	0.0278	7.1×10^{-4}
DSb	COMInkLength	0.0932	0.0278	8.3×10^{-4}
DSb	COPDrawingEfficiency	0.0942	0.0282	8.5×10^{-4}
DSb	COPDrawingEfficiency_s	0.0885	0.0279	1.5×10^{-3}
DSb	COMNoise	-0.0848	0.0285	3.0×10^{-3}
DSb	COMOscillatoryMotion_s	0.0832	0.0285	3.6×10^{-3}
DSb	COPSpatialReasoning	0.0801	0.0281	4.4×10^{-3}
DSb	COPSpatialReasoning_s	0.0759	0.0277	6.2×10^{-3}
DSb	COMOscillatoryMotion	-0.0806	0.0295	6.3×10^{-3}
DSb	COMSimpleMotor	0.0750	0.0283	8.3×10^{-3}
DSb	COPNoise	-0.0721	0.0283	1.1×10^{-2}
DSb	COMSimpleMotor_s	0.0689	0.0279	1.4×10^{-2}
DSb	COPDrawingProcessEfficiency	0.0689	0.0283	1.5×10^{-2}
DSb	COPDrawingSize_s	0.0667	0.0275	1.5×10^{-2}
DSb	COMNoise_s	0.0670	0.0279	1.7×10^{-2}
DSb	COPDrawingSize	0.0639	0.0275	2.0×10^{-2}
DSb	COPDrawingProcessEfficiency_s	0.0622	0.0280	2.6×10^{-2}
DSb	COMStrokeCountConformity	-0.0619	0.0291	3.4×10^{-2}
DSb	COPStrokeCountConformity	-0.0632	0.0302	3.7×10^{-2}
DSb	COPTotalTime	-0.0588	0.0289	4.2×10^{-2}

DSb	COMHorizontalSpatialPlacement_s	0.0542	0.0273	4.8×10^{-2}
DSb	COPRelativeLongLatency_s	0.0554	0.0283	5.1×10^{-2}
DSb	COPRelativeLongLatency	-0.0551	0.0286	5.4×10^{-2}
DSb	COPTotalTime_s	0.0539	0.0281	5.6×10^{-2}
DSb	COPInformationProcessing	0.0511	0.0285	7.3×10^{-2}
DSb	COPAverageLatency	-0.0512	0.0286	7.3×10^{-2}
DSb	COMHorizontalSpatialPlacement	-0.0487	0.0273	7.5×10^{-2}
DSb	COPAverageLatency_s	0.0499	0.0281	7.6×10^{-2}
DSb	COMInitiationSpeed	0.0473	0.0275	8.6×10^{-2}
DSb	COPLongLatencyCount	-0.0503	0.0292	8.6×10^{-2}
DSb	COPNoise_s	0.0474	0.0281	9.1×10^{-2}
DSb	COMInitiationSpeed_s	0.0446	0.0275	1.0×10^{-1}
DSb	COPPercentInkTime	0.0436	0.0281	1.2×10^{-1}
DSb	COMMaxSpeed	0.0412	0.0274	1.3×10^{-1}
DSb	COPPercentThinkTime_s	0.0421	0.0281	1.3×10^{-1}
DSb	COPInformationProcessing_s	0.0421	0.0282	1.4×10^{-1}
DSb	COMAverageSpeed	0.0410	0.0275	1.4×10^{-1}
DSb	COPVerticalSpatialPlacement	0.0410	0.0275	1.4×10^{-1}
DSb	COMVerticalSpatialPlacement_s	0.0384	0.0273	1.6×10^{-1}
DSb	COMTerminationSpeed	0.0384	0.0275	1.6×10^{-1}
DSb	COMMaxSpeed_s	0.0381	0.0273	1.6×10^{-1}
DSb	COMAverageSpeed_s	0.0379	0.0275	1.7×10^{-1}
DSb	COPVerticalSpatialPlacement_s	-0.0354	0.0274	2.0×10^{-1}
DSb	COMTerminationSpeed_s	0.0353	0.0275	2.0×10^{-1}
DSb	COMVerticalSpatialPlacement	-0.0351	0.0273	2.0×10^{-1}
DSb	COPPercentThinkTime	-0.0353	0.0282	2.1×10^{-1}
DSb	COPPercentInkTime_s	-0.0343	0.0282	2.2×10^{-1}
DSb	COPLatencyVariability	-0.0332	0.0284	2.4×10^{-1}
DSb	COPLatencyVariability_s	0.0319	0.0281	2.6×10^{-1}
DSb	COPOscillatoryMotion_s	0.0279	0.0278	3.2×10^{-1}
DSb	COPOscillatoryMotion	-0.0285	0.0287	3.2×10^{-1}
DSb	COPSimpleMotor	0.0277	0.0283	3.3×10^{-1}
DSb	COPLongLatencyCount_s	0.0273	0.0279	3.3×10^{-1}
DSb	COPLongestLatency_s	0.0226	0.0280	4.2×10^{-1}
DSb	COPLongestLatency	-0.0221	0.0283	4.4×10^{-1}
DSb	COPHorizontalSpatialPlacement	0.0202	0.0275	4.6×10^{-1}
DSb	COPSimpleMotor_s	0.0188	0.0276	5.0×10^{-1}
DSb	COMStrokeCountConformity_s	0.0163	0.0277	5.6×10^{-1}
DSb	COPHorizontalSpatialPlacement_s	-0.0147	0.0275	5.9×10^{-1}
DSb	COPMaxSpeed_s	-0.0144	0.0274	6.0×10^{-1}
DSb	COPInkLength	0.0137	0.0278	6.2×10^{-1}
DSb	COPInkLength_s	0.0131	0.0276	6.4×10^{-1}
DSb	COPTerminationSpeed	0.0105	0.0277	7.0×10^{-1}

DSb	COPInitiationSpeed_s	-0.0088	0.0275	7.5×10^{-1}
DSb	COPMaxSpeed	-0.0085	0.0275	7.6×10^{-1}
DSb	COPStrokeCountConformity_s	0.0070	0.0277	8.0×10^{-1}
DSb	COPAverageSpeed_s	-0.0061	0.0275	8.2×10^{-1}
DSb	COPTerminationSpeed_s	0.0050	0.0276	8.6×10^{-1}
DSb	COPInitiationSpeed	-0.0038	0.0276	8.9×10^{-1}
DSb	COPAverageSpeed	-0.0005	0.0276	9.9×10^{-1}
Trails A	COPTotalTime	5.8693	0.3425	$\mathbf{1.4\times10^{-61}}$
Trails A	COPAverageLatency	5.7823	0.3381	$\mathbf{2.3\times10^{-61}}$
Trails A	COPTotalTime_s	-5.6118	0.3338	$\mathbf{1.7\times10^{-59}}$
Trails A	COPAverageLatency_s	-5.5551	0.3343	$\mathbf{3.0\times10^{-58}}$
Trails A	COPDrawingProcessEfficiency	-5.5321	0.3367	$\mathbf{4.7\times10^{-57}}$
Trails A	COPDrawingProcessEfficiency_s	-5.4572	0.3333	$\mathbf{1.0\times10^{-56}}$
Trails A	COPRelativeLongLatency	5.3965	0.3414	$\mathbf{3.6\times10^{-53}}$
Trails A	COPInformationProcessing	-5.3130	0.3408	$\mathbf{7.2\times10^{-52}}$
Trails A	COPRelativeLongLatency_s	-5.1639	0.3390	$\mathbf{1.1\times10^{-49}}$
Trails A	COPInformationProcessing_s	-5.1268	0.3385	$\mathbf{3.5\times10^{-49}}$
Trails A	COPLatencyVariability	4.9931	0.3413	$\mathbf{3.6\times10^{-46}}$
Trails A	COMDrawingProcessEfficiency	-4.9612	0.3399	$\mathbf{5.7\times10^{-46}}$
Trails A	COPDrawingEfficiency_s	-4.8463	0.3380	$\mathbf{1.7\times10^{-44}}$
Trails A	COMTotalTime	4.8657	0.3409	$\mathbf{3.9\times10^{-44}}$
Trails A	COPDrawingEfficiency	-4.8643	0.3417	$\mathbf{6.6\times10^{-44}}$
Trails A	COMDrawingProcessEfficiency_s	-4.7277	0.3355	$\mathbf{4.2\times10^{-43}}$
Trails A	DCTScore	-4.9328	0.3527	$\mathbf{1.6\times10^{-42}}$
Trails A	COMTotalTime_s	-4.6773	0.3348	$\mathbf{2.0\times10^{-42}}$
Trails A	COPLatencyVariability_s	-4.7316	0.3395	$\mathbf{3.1\times10^{-42}}$
Trails A	COPLongLatencyCount	4.8675	0.3547	$\mathbf{4.6\times10^{-41}}$
Trails A	COMDrawingEfficiency	-4.5288	0.3402	$\mathbf{7.9\times10^{-39}}$
Trails A	COMAverageLatency	4.5225	0.3428	$\mathbf{3.4\times10^{-38}}$
Trails A	COPLongestLatency	4.5211	0.3428	$\mathbf{3.6\times10^{-38}}$
Trails A	COMDrawingEfficiency_s	-4.4451	0.3371	$\mathbf{3.7\times10^{-38}}$
Trails A	COPLongLatencyCount_s	-4.4062	0.3392	$\mathbf{3.9\times10^{-37}}$
Trails A	COMAverageLatency_s	-4.3475	0.3369	$\mathbf{1.1\times10^{-36}}$
Trails A	COPLongestLatency_s	-4.2937	0.3404	$\mathbf{3.5\times10^{-35}}$
Trails A	COMRelativeLongLatency	3.8545	0.3422	$\mathbf{1.4\times10^{-28}}$
Trails A	COMInformationProcessing	-3.7956	0.3426	$\mathbf{9.5\times10^{-28}}$
Trails A	COMInformationProcessing_s	-3.6664	0.3387	$\mathbf{1.3\times10^{-26}}$
Trails A	COMAverageSpeed	-3.6397	0.3381	$\mathbf{2.5\times10^{-26}}$
Trails A	COMRelativeLongLatency_s	-3.6428	0.3391	$\mathbf{3.2\times10^{-26}}$
Trails A	COMAverageSpeed_s	-3.5773	0.3382	$\mathbf{1.7\times10^{-25}}$
Trails A	COPAverageSpeed	-3.5221	0.3400	$\mathbf{1.5\times10^{-24}}$
Trails A	COMTerminationSpeed	-3.4898	0.3385	$\mathbf{2.5\times10^{-24}}$

Trails A	COMSimpleMotor	-3.5672	0.3486	**5.3×10^{-24}**
Trails A	COMTerminationSpeed_s	-3.4211	0.3382	**1.7×10^{-23}**
Trails A	COMSimpleMotor_s	-3.4416	0.3437	**4.5×10^{-23}**
Trails A	COPAverageSpeed_s	-3.3940	0.3390	**4.6×10^{-23}**
Trails A	COPSimpleMotor	-3.4232	0.3488	**3.0×10^{-22}**
Trails A	COPInitiationSpeed	-3.3182	0.3392	**4.0×10^{-22}**
Trails A	COMLatencyVariability	3.3239	0.3434	**1.1×10^{-21}**
Trails A	COPTerminationSpeed	-3.3011	0.3418	**1.3×10^{-21}**
Trails A	COMInitiationSpeed	-3.2691	0.3392	**1.6×10^{-21}**
Trails A	COPSimpleMotor_s	-3.2737	0.3410	**2.3×10^{-21}**
Trails A	COMInitiationSpeed_s	-3.1988	0.3394	**1.1×10^{-20}**
Trails A	COPInitiationSpeed_s	-3.1725	0.3386	**1.9×10^{-20}**
Trails A	COPTerminationSpeed_s	-3.1849	0.3410	**2.4×10^{-20}**
Trails A	COMLatencyVariability_s	-3.1587	0.3396	**3.4×10^{-20}**
Trails A	COMMaxSpeed	-3.1285	0.3383	**5.6×10^{-20}**
Trails A	COMLongLatencyCount_s	-3.1763	0.3440	**6.4×10^{-20}**
Trails A	COPMaxSpeed	-3.0870	0.3400	**2.5×10^{-19}**
Trails A	COMMaxSpeed_s	-3.0499	0.3383	**4.4×10^{-19}**
Trails A	COMLongLatencyCount	3.1982	0.3592	**1.2×10^{-18}**
Trails A	COMLongestLatency	3.0373	0.3430	**1.8×10^{-18}**
Trails A	COPMaxSpeed_s	-2.9324	0.3395	**1.1×10^{-17}**
Trails A	COMLongestLatency_s	-2.8683	0.3398	**5.9×10^{-17}**
Trails A	COMComponentPlacement_s	-2.8654	0.3488	**3.7×10^{-16}**
Trails A	COMComponentPlacement	2.8131	0.3565	**4.9×10^{-15}**
Trails A	COMSpatialReasoning_s	-2.6287	0.3468	**5.3×10^{-14}**
Trails A	COMSpatialReasoning	-2.5343	0.3531	**9.9×10^{-13}**
Trails A	COPPercentThinkTime	2.2867	0.3507	**8.9×10^{-11}**
Trails A	COPPercentInkTime	-2.2495	0.3500	**1.6×10^{-10}**
Trails A	COPPercentInkTime_s	2.2482	0.3513	**1.9×10^{-10}**
Trails A	COPPercentThinkTime_s	-2.2084	0.3506	**3.7×10^{-10}**
Trails A	COPSpatialReasoning	-2.1435	0.3519	**1.3×10^{-9}**
Trails A	COPSpatialReasoning_s	-2.0311	0.3471	**5.7×10^{-9}**
Trails A	COMNoise	2.0016	0.3569	**2.3×10^{-8}**
Trails A	COPComponentPlacement	1.9568	0.3532	**3.4×10^{-8}**
Trails A	COPComponentPlacement_s	-1.9175	0.3462	**3.4×10^{-8}**
Trails A	COMNoise_s	-1.8095	0.3496	**2.5×10^{-7}**
Trails A	COMOscillatoryMotion_s	-1.7081	0.3562	**1.7×10^{-6}**
Trails A	COMOscillatoryMotion	1.7398	0.3682	**2.5×10^{-6}**
Trails A	COPNoise	1.6794	0.3557	**2.5×10^{-6}**
Trails A	COPNoise_s	-1.6321	0.3510	**3.5×10^{-6}**
Trails A	COPOscillatoryMotion_s	-1.6117	0.3485	**4.0×10^{-6}**
Trails A	COPOscillatoryMotion	1.6040	0.3587	**8.2×10^{-6}**
Trails A	COMPercentThinkTime	1.4584	0.3478	**2.9×10^{-5}**

Trails A	COMPercentInkTime	-1.4447	0.3476	**3.4×10^{-5}**
Trails A	COMPercentInkTime_s	1.3763	0.3481	**8.0×10^{-5}**
Trails A	COMPercentThinkTime_s	-1.3636	0.3478	**9.1×10^{-5}**
Trails A	COPDrawingSize_s	-1.1079	0.3464	1.4×10^{-3}
Trails A	COPStrokeCountConformity	1.2014	0.3810	1.6×10^{-3}
Trails A	COPDrawingSize	-1.0684	0.3467	2.1×10^{-3}
Trails A	COPVerticalSpatialPlacement_s	-1.0649	0.3459	2.1×10^{-3}
Trails A	COPVerticalSpatialPlacement	1.0599	0.3471	2.3×10^{-3}
Trails A	COMClockfaceCircularity_s	-1.0432	0.3450	2.5×10^{-3}
Trails A	COMClockfaceCircularity	0.9968	0.3481	4.2×10^{-3}
Trails A	COMStrokeCountConformity	0.9421	0.3647	9.9×10^{-3}
Trails A	COPClockfaceCircularity_s	-0.8508	0.3467	1.4×10^{-2}
Trails A	COMVerticalSpatialPlacement_s	-0.8295	0.3425	1.6×10^{-2}
Trails A	COMStrokeCountConformity_s	-0.7972	0.3488	2.2×10^{-2}
Trails A	COMDrawingSize	-0.7768	0.3500	2.7×10^{-2}
Trails A	COMDrawingSize_s	-0.7510	0.3493	3.2×10^{-2}
Trails A	COMVerticalSpatialPlacement	0.7361	0.3434	3.2×10^{-2}
Trails A	COPStrokeCountConformity_s	-0.7222	0.3496	3.9×10^{-2}
Trails A	COPClockfaceCircularity	0.6993	0.3475	4.4×10^{-2}
Trails A	COMInkLength	-0.5064	0.3512	1.5×10^{-1}
Trails A	COMInkLength_s	-0.5021	0.3513	1.5×10^{-1}
Trails A	COPHorizontalSpatialPlacement_s	-0.4820	0.3469	1.6×10^{-1}
Trails A	COPHorizontalSpatialPlacement	0.4492	0.3461	1.9×10^{-1}
Trails A	COPInkLength_s	-0.4366	0.3488	2.1×10^{-1}
Trails A	COPInkLength	-0.4289	0.3510	2.2×10^{-1}
Trails A	COMHorizontalSpatialPlacement_s	-0.3892	0.3435	2.6×10^{-1}
Trails A	COMHorizontalSpatialPlacement	0.3198	0.3434	3.5×10^{-1}
Trails B	DCTScore	-21.7051	1.5558	**2.8×10^{-42}**
Trails B	COMDrawingProcessEfficiency	-18.4567	1.5177	**7.1×10^{-33}**
Trails B	COPAverageLatency	17.7924	1.5472	**1.1×10^{-29}**
Trails B	COMDrawingProcessEfficiency_s	-16.9340	1.5020	**1.3×10^{-28}**
Trails B	COMTotalTime	17.1034	1.5256	**2.5×10^{-28}**
Trails B	COMAverageLatency	17.1123	1.5307	**3.5×10^{-28}**
Trails B	COPInformationProcessing	-16.8734	1.5480	**6.5×10^{-27}**
Trails B	COMDrawingEfficiency	-16.4883	1.5128	**6.5×10^{-27}**
Trails B	COPDrawingProcessEfficiency	-16.5241	1.5325	**2.2×10^{-26}**
Trails B	COMAverageLatency_s	-16.1927	1.5043	**2.6×10^{-26}**
Trails B	COPRelativeLongLatency	16.6862	1.5561	**4.0×10^{-26}**
Trails B	COMTotalTime_s	-15.9763	1.5005	**8.7×10^{-26}**
Trails B	COPAverageLatency_s	-16.2356	1.5333	**1.6×10^{-25}**
Trails B	COMInformationProcessing	-16.0290	1.5157	**1.8×10^{-25}**
Trails B	COPTotalTime	16.4484	1.5715	**5.4×10^{-25}**

Trails B	COMDrawingEfficiency_s	-15.7126	1.5014	**5.5×10^{-25}**
Trails B	COPDrawingProcessEfficiency_s	-15.8023	1.5204	**1.1×10^{-24}**
Trails B	COMRelativeLongLatency	15.6889	1.5137	**1.5×10^{-24}**
Trails B	COPLatencyVariability	16.0074	1.5465	**1.7×10^{-24}**
Trails B	COMComponentPlacement_s	-15.4587	1.5272	**1.6×10^{-23}**
Trails B	COMInformationProcessing_s	-15.1355	1.5006	**2.3×10^{-23}**
Trails B	COPInformationProcessing_s	-15.3737	1.5431	**7.5×10^{-23}**
Trails B	COPLongLatencyCount	15.7101	1.5991	**2.8×10^{-22}**
Trails B	COMLatencyVariability	14.8878	1.5171	**3.1×10^{-22}**
Trails B	COPRelativeLongLatency_s	-15.1336	1.5460	**3.9×10^{-22}**
Trails B	COMComponentPlacement	15.1905	1.5617	**7.0×10^{-22}**
Trails B	COMSpatialReasoning_s	-14.6750	1.5184	**1.3×10^{-21}**
Trails B	COPTotalTime_s	-14.8312	1.5347	**1.3×10^{-21}**
Trails B	COMRelativeLongLatency_s	-14.4580	1.5011	**1.7×10^{-21}**
Trails B	COPDrawingEfficiency	-14.7617	1.5431	**3.1×10^{-21}**
Trails B	COPDrawingEfficiency_s	-14.4935	1.5293	**7.1×10^{-21}**
Trails B	COMLongestLatency	14.2539	1.5118	**1.1×10^{-20}**
Trails B	COPLongestLatency	14.5224	1.5451	**1.5×10^{-20}**
Trails B	COPLongLatencyCount_s	-14.3237	1.5292	**2.0×10^{-20}**
Trails B	COMLatencyVariability_s	-13.9139	1.5009	**4.7×10^{-20}**
Trails B	COMSpatialReasoning	-14.2881	1.5474	**6.5×10^{-20}**
Trails B	COMLongLatencyCount_s	-13.7960	1.5194	**2.5×10^{-19}**
Trails B	COPLatencyVariability_s	-13.9242	1.5411	**3.8×10^{-19}**
Trails B	COMLongestLatency_s	-13.2792	1.4981	**1.7×10^{-18}**
Trails B	COPSpatialReasoning	-13.2584	1.5368	**1.3×10^{-17}**
Trails B	COPLongestLatency_s	-12.6917	1.5366	**2.6×10^{-16}**
Trails B	COPSpatialReasoning_s	-12.4816	1.5174	**3.5×10^{-16}**
Trails B	COMLongLatencyCount	12.9487	1.5922	**7.3×10^{-16}**
Trails B	COPComponentPlacement_s	-11.6186	1.5188	**3.1×10^{-14}**
Trails B	COPComponentPlacement	11.8318	1.5485	**3.3×10^{-14}**
Trails B	COMSimpleMotor	-11.3193	1.5515	**4.3×10^{-13}**
Trails B	COMAverageSpeed	-10.9189	1.5065	**6.0×10^{-13}**
Trails B	COMInitiationSpeed	-10.7254	1.5076	**1.6×10^{-12}**
Trails B	COPSimpleMotor	-10.8014	1.5552	**5.1×10^{-12}**
Trails B	COMAverageSpeed_s	-10.3045	1.5083	**1.1×10^{-11}**
Trails B	COMInitiationSpeed_s	-10.1944	1.5093	**1.9×10^{-11}**
Trails B	COMSimpleMotor_s	-10.2261	1.5313	**3.1×10^{-11}**
Trails B	COPAverageSpeed	-9.8820	1.5117	**8.0×10^{-11}**
Trails B	COMTerminationSpeed	-9.6187	1.5065	**2.1×10^{-10}**
Trails B	COPInitiationSpeed	-9.5920	1.5034	**2.2×10^{-10}**
Trails B	COPSimpleMotor_s	-9.5143	1.5235	**5.2×10^{-10}**
Trails B	COMMaxSpeed	-9.1656	1.4976	**1.1×10^{-9}**
Trails B	COMTerminationSpeed_s	-8.9192	1.5058	**3.7×10^{-9}**

Trails B	COPTerminationSpeed	-8.8761	1.5200	**6.1×10^{-9}**
Trails B	COPAverageSpeed_s	-8.7260	1.5092	**8.6×10^{-9}**
Trails B	COPMaxSpeed	-8.6584	1.5054	**1.0×10^{-8}**
Trails B	COPInitiationSpeed_s	-8.4764	1.5016	**1.9×10^{-8}**
Trails B	COMMaxSpeed_s	-8.4201	1.4986	**2.2×10^{-8}**
Trails B	COPPercentThinkTime	8.3399	1.5538	**8.9×10^{-8}**
Trails B	COPPercentInkTime	-8.2071	1.5502	**1.3×10^{-7}**
Trails B	COMPercentInkTime	-8.0815	1.5278	**1.4×10^{-7}**
Trails B	COMPercentThinkTime	8.0685	1.5290	**1.5×10^{-7}**
Trails B	COPTerminationSpeed_s	-7.9667	1.5174	**1.7×10^{-7}**
Trails B	COPPercentInkTime_s	7.9265	1.5561	**3.8×10^{-7}**
Trails B	COPPercentThinkTime_s	-7.7896	1.5527	**5.7×10^{-7}**
Trails B	COPMaxSpeed_s	-7.4367	1.5043	**8.3×10^{-7}**
Trails B	COMPercentThinkTime_s	-7.4887	1.5296	**1.1×10^{-6}**
Trails B	COMPercentInkTime_s	7.4671	1.5311	**1.2×10^{-6}**
Trails B	COPDrawingSize_s	-7.2378	1.5262	**2.3×10^{-6}**
Trails B	COMNoise	7.3479	1.5735	**3.2×10^{-6}**
Trails B	COMNoise_s	-7.0397	1.5361	**4.9×10^{-6}**
Trails B	COPDrawingSize	-6.9634	1.5294	**5.6×10^{-6}**
Trails B	COMDrawingSize	-6.9281	1.5360	**6.8×10^{-6}**
Trails B	COMOscillatoryMotion	7.1446	1.6236	**1.1×10^{-5}**
Trails B	COMDrawingSize_s	-6.7184	1.5334	**1.2×10^{-5}**
Trails B	COPClockfaceCircularity_s	-6.5619	1.5183	**1.6×10^{-5}**
Trails B	COPVerticalSpatialPlacement_s	-5.8072	1.5205	**1.4×10^{-4}**
Trails B	COMOscillatoryMotion_s	-5.9898	1.5721	**1.4×10^{-4}**
Trails B	COPOscillatoryMotion_s	-5.8442	1.5414	**1.5×10^{-4}**
Trails B	COPVerticalSpatialPlacement	5.6218	1.5252	**2.3×10^{-4}**
Trails B	COPOscillatoryMotion	5.6889	1.5883	**3.5×10^{-4}**
Trails B	COPClockfaceCircularity	5.1500	1.5238	7.4×10^{-4}
Trails B	COMClockfaceCircularity	5.1199	1.5336	8.6×10^{-4}
Trails B	COMClockfaceCircularity_s	-4.6393	1.5197	2.3×10^{-3}
Trails B	COMHorizontalSpatialPlacement	4.2698	1.5070	4.7×10^{-3}
Trails B	COPNoise	4.3437	1.5693	5.7×10^{-3}
Trails B	COMHorizontalSpatialPlacement_s	-4.1385	1.5085	6.1×10^{-3}
Trails B	COMStrokeCountConformity	4.3302	1.6040	7.0×10^{-3}
Trails B	COMVerticalSpatialPlacement	3.9310	1.5087	9.2×10^{-3}
Trails B	COMVerticalSpatialPlacement_s	-3.7440	1.5057	1.3×10^{-2}
Trails B	COMStrokeCountConformity_s	-3.6386	1.5367	1.8×10^{-2}
Trails B	COMInkLength_s	-3.6043	1.5437	2.0×10^{-2}
Trails B	COMInkLength	-3.4989	1.5435	2.4×10^{-2}
Trails B	COPNoise_s	-3.4988	1.5596	2.5×10^{-2}
Trails B	COPInkLength_s	-3.4213	1.5356	2.6×10^{-2}
Trails B	COPStrokeCountConformity	3.6283	1.6727	3.0×10^{-2}

Trails B	COPInkLength	-3.2264	1.5457	3.7×10^{-2}
Trails B	COPHorizontalSpatialPlacement_s	-1.3685	1.5197	3.7×10^{-1}
Trails B	COPHorizontalSpatialPlacement	0.8961	1.5170	5.5×10^{-1}
Trails B	COPStrokeCountConformity_s	-0.6418	1.5454	6.8×10^{-1}
SIM	COMSpatialReasoning_s	0.7781	0.0768	$\mathbf{1.4\times10^{-23}}$
SIM	COMSpatialReasoning	0.7799	0.0782	$\mathbf{6.3\times10^{-23}}$
SIM	DCTScore	0.7710	0.0805	$\mathbf{2.7\times10^{-21}}$
SIM	COMComponentPlacement_s	0.7406	0.0777	$\mathbf{4.3\times10^{-21}}$
SIM	COMComponentPlacement	-0.7461	0.0794	$\mathbf{1.5\times10^{-20}}$
SIM	COMDrawingProcessEfficiency	0.5832	0.0784	$\mathbf{1.5\times10^{-13}}$
SIM	COMDrawingEfficiency	0.5738	0.0780	$\mathbf{2.7\times10^{-13}}$
SIM	COMDrawingEfficiency_s	0.5370	0.0773	$\mathbf{5.1\times10^{-12}}$
SIM	COMDrawingProcessEfficiency_s	0.5300	0.0774	$\mathbf{1.0\times10^{-11}}$
SIM	COPSpatialReasoning	0.4646	0.0785	$\mathbf{3.8\times10^{-9}}$
SIM	COMLatencyVariability	-0.4413	0.0781	$\mathbf{1.8\times10^{-8}}$
SIM	COPSpatialReasoning_s	0.4330	0.0774	$\mathbf{2.5\times10^{-8}}$
SIM	COMInformationProcessing	0.4374	0.0783	$\mathbf{2.7\times10^{-8}}$
SIM	COMLongestLatency	-0.4237	0.0777	$\mathbf{5.6\times10^{-8}}$
SIM	COMRelativeLongLatency	-0.4116	0.0784	$\mathbf{1.7\times10^{-7}}$
SIM	COMLatencyVariability_s	0.4048	0.0772	$\mathbf{1.7\times10^{-7}}$
SIM	COPComponentPlacement_s	0.3974	0.0775	$\mathbf{3.2\times10^{-7}}$
SIM	COMInformationProcessing_s	0.3966	0.0774	$\mathbf{3.3\times10^{-7}}$
SIM	COPClockfaceCircularity_s	0.3932	0.0770	$\mathbf{3.6\times10^{-7}}$
SIM	COMAverageLatency	-0.4022	0.0793	$\mathbf{4.3\times10^{-7}}$
SIM	COMLongestLatency_s	0.3888	0.0769	$\mathbf{4.7\times10^{-7}}$
SIM	COMDrawingSize	0.3933	0.0779	$\mathbf{4.8\times10^{-7}}$
SIM	COPComponentPlacement	-0.3964	0.0791	$\mathbf{5.9\times10^{-7}}$
SIM	COMDrawingSize_s	0.3836	0.0778	$\mathbf{8.8\times10^{-7}}$
SIM	COMLongLatencyCount_s	0.3741	0.0779	$\mathbf{1.7\times10^{-6}}$
SIM	COMRelativeLongLatency_s	0.3699	0.0775	$\mathbf{2.0\times10^{-6}}$
SIM	COMLongLatencyCount	-0.3826	0.0814	$\mathbf{2.8\times10^{-6}}$
SIM	COMAverageLatency_s	0.3625	0.0779	$\mathbf{3.5\times10^{-6}}$
SIM	COMOscillatoryMotion_s	0.3589	0.0796	$\mathbf{6.8\times10^{-6}}$
SIM	COPClockfaceCircularity	-0.3471	0.0773	$\mathbf{7.6\times10^{-6}}$
SIM	COMSimpleMotor	0.3553	0.0796	$\mathbf{8.5\times10^{-6}}$
SIM	COMOscillatoryMotion	-0.3620	0.0822	$\mathbf{1.1\times10^{-5}}$
SIM	COMTotalTime	-0.3479	0.0794	$\mathbf{1.2\times10^{-5}}$
SIM	COMClockfaceCircularity_s	0.3323	0.0771	$\mathbf{1.7\times10^{-5}}$
SIM	COMClockfaceCircularity	-0.3283	0.0778	$\mathbf{2.6\times10^{-5}}$
SIM	COMInkLength_s	0.3267	0.0783	$\mathbf{3.1\times10^{-5}}$
SIM	COMInkLength	0.3232	0.0783	$\mathbf{3.8\times10^{-5}}$
SIM	COMSimpleMotor_s	0.3128	0.0784	$\mathbf{6.9\times10^{-5}}$

SIM	COMTotalTime_s	0.3016	0.0780	**1.1×10^{-4}**
SIM	COMVerticalSpatialPlacement_s	0.2858	0.0764	**1.9×10^{-4}**
SIM	COMInitiationSpeed	0.2874	0.0772	**2.0×10^{-4}**
SIM	COMHorizontalSpatialPlacement_s	0.2852	0.0766	**2.0×10^{-4}**
SIM	COMPercentInkTime	0.2804	0.0781	**3.4×10^{-4}**
SIM	COPDrawingEfficiency	0.2807	0.0793	**4.1×10^{-4}**
SIM	COMPercentThinkTime	-0.2753	0.0781	**4.3×10^{-4}**
SIM	COPOscillatoryMotion_s	0.2745	0.0779	**4.4×10^{-4}**
SIM	COPOscillatoryMotion	-0.2786	0.0802	5.3×10^{-4}
SIM	COPSimpleMotor	0.2657	0.0792	8.1×10^{-4}
SIM	COMHorizontalSpatialPlacement	-0.2571	0.0767	8.2×10^{-4}
SIM	COPDrawingProcessEfficiency	0.2644	0.0793	8.7×10^{-4}
SIM	COMPercentThinkTime_s	0.2592	0.0781	9.1×10^{-4}
SIM	COMInitiationSpeed_s	0.2563	0.0772	9.2×10^{-4}
SIM	COMNoise	-0.2632	0.0799	1.0×10^{-3}
SIM	COMPercentInkTime_s	-0.2552	0.0781	1.1×10^{-3}
SIM	COMVerticalSpatialPlacement	-0.2468	0.0767	1.3×10^{-3}
SIM	COPVerticalSpatialPlacement	-0.2416	0.0771	1.8×10^{-3}
SIM	COPVerticalSpatialPlacement_s	0.2370	0.0770	2.1×10^{-3}
SIM	COMAverageSpeed	0.2382	0.0774	2.1×10^{-3}
SIM	COPDrawingEfficiency_s	0.2387	0.0785	2.4×10^{-3}
SIM	COPDrawingSize_s	0.2338	0.0771	2.4×10^{-3}
SIM	COPDrawingSize	0.2180	0.0772	4.8×10^{-3}
SIM	COPLongLatencyCount	-0.2317	0.0822	4.8×10^{-3}
SIM	COMNoise_s	0.2153	0.0784	6.1×10^{-3}
SIM	COPTotalTime	-0.2183	0.0812	7.2×10^{-3}
SIM	COPDrawingProcessEfficiency_s	0.2096	0.0785	7.7×10^{-3}
SIM	COPSimpleMotor_s	0.2034	0.0775	8.7×10^{-3}
SIM	COMMaxSpeed	0.1991	0.0770	9.8×10^{-3}
SIM	COMAverageSpeed_s	0.2001	0.0774	9.8×10^{-3}
SIM	COPInformationProcessing	0.1913	0.0800	1.7×10^{-2}
SIM	COMTerminationSpeed	0.1843	0.0775	1.7×10^{-2}
SIM	COPAverageLatency	-0.1859	0.0802	2.0×10^{-2}
SIM	COPStrokeCountConformity_s	-0.1652	0.0780	3.4×10^{-2}
SIM	COMMaxSpeed_s	0.1617	0.0770	3.6×10^{-2}
SIM	COMTerminationSpeed_s	0.1483	0.0773	5.5×10^{-2}
SIM	COPTotalTime_s	0.1502	0.0790	5.7×10^{-2}
SIM	COPLongestLatency	-0.1507	0.0795	5.8×10^{-2}
SIM	COPRelativeLongLatency	-0.1385	0.0803	8.5×10^{-2}
SIM	COPLatencyVariability	-0.1346	0.0798	9.2×10^{-2}
SIM	COPAverageLatency_s	0.1278	0.0791	1.1×10^{-1}
SIM	COPLongLatencyCount_s	0.1261	0.0783	1.1×10^{-1}
SIM	COPAverageSpeed	0.1191	0.0777	1.3×10^{-1}

SIM	COPInformationProcessing_s	0.1177	0.0793	1.4×10^{-1}
SIM	COPTerminationSpeed	0.1097	0.0778	1.6×10^{-1}
SIM	COPNoise_s	0.1097	0.0788	1.6×10^{-1}
SIM	COPLongestLatency_s	0.1032	0.0787	1.9×10^{-1}
SIM	COPHorizontalSpatialPlacement_s	-0.0999	0.0772	2.0×10^{-1}
SIM	COPInitiationSpeed	0.0999	0.0776	2.0×10^{-1}
SIM	COPMaxSpeed	0.0995	0.0773	2.0×10^{-1}
SIM	COPHorizontalSpatialPlacement	0.0984	0.0770	2.0×10^{-1}
SIM	COPInkLength_s	0.0978	0.0774	2.1×10^{-1}
SIM	COPNoise	-0.0970	0.0796	2.2×10^{-1}
SIM	COPInkLength	0.0877	0.0779	2.6×10^{-1}
SIM	COPRelativeLongLatency_s	0.0892	0.0795	2.6×10^{-1}
SIM	COPLatencyVariability_s	0.0839	0.0791	2.9×10^{-1}
SIM	COMStrokeCountConformity	-0.0673	0.0820	4.1×10^{-1}
SIM	COPTerminationSpeed_s	0.0633	0.0775	4.1×10^{-1}
SIM	COMStrokeCountConformity_s	-0.0598	0.0780	4.4×10^{-1}
SIM	COPAverageSpeed_s	0.0586	0.0775	4.5×10^{-1}
SIM	COPInitiationSpeed_s	0.0496	0.0774	5.2×10^{-1}
SIM	COPMaxSpeed_s	0.0436	0.0771	5.7×10^{-1}
SIM	COPPercentInkTime	0.0291	0.0788	7.1×10^{-1}
SIM	COPPercentThinkTime_s	0.0162	0.0789	8.4×10^{-1}
SIM	COPStrokeCountConformity	0.0161	0.0851	8.5×10^{-1}
SIM	COPPercentThinkTime	-0.0076	0.0790	9.2×10^{-1}
SIM	COPPercentInkTime_s	0.0052	0.0791	9.5×10^{-1}
HVOT	DCTScore	0.8695	0.0653	**7.8×10^{-39}**
HVOT	COMComponentPlacement_s	0.7907	0.0633	**1.6×10^{-34}**
HVOT	COMComponentPlacement	-0.8006	0.0644	**2.9×10^{-34}**
HVOT	COMSpatialReasoning_s	0.7792	0.0628	**4.2×10^{-34}**
HVOT	COMSpatialReasoning	0.7876	0.0636	**5.4×10^{-34}**
HVOT	COPComponentPlacement	-0.5713	0.0646	**2.1×10^{-18}**
HVOT	COPComponentPlacement_s	0.5582	0.0634	**2.8×10^{-18}**
HVOT	COPSpatialReasoning	0.5516	0.0643	**1.8×10^{-17}**
HVOT	COMDrawingProcessEfficiency	0.5498	0.0643	**2.5×10^{-17}**
HVOT	COMDrawingEfficiency	0.5347	0.0639	**1.1×10^{-16}**
HVOT	COPSpatialReasoning_s	0.5276	0.0634	**1.6×10^{-16}**
HVOT	COMDrawingEfficiency_s	0.5242	0.0633	**2.3×10^{-16}**
HVOT	COMDrawingProcessEfficiency_s	0.5228	0.0633	**2.7×10^{-16}**
HVOT	COMRelativeLongLatency	-0.5243	0.0640	**4.7×10^{-16}**
HVOT	COMLatencyVariability	-0.5143	0.0638	**1.2×10^{-15}**
HVOT	COMInformationProcessing	0.5142	0.0641	**1.7×10^{-15}**
HVOT	COMTotalTime	-0.5178	0.0650	**2.7×10^{-15}**
HVOT	COMRelativeLongLatency_s	0.5022	0.0633	**3.4×10^{-15}**

HVOT	COMLatencyVariability_s	0.4949	0.0630	**6.2×10^{-15}**
HVOT	COMInformationProcessing_s	0.4948	0.0633	**8.7×10^{-15}**
HVOT	COMLongestLatency	-0.4954	0.0636	**1.1×10^{-14}**
HVOT	COMTotalTime_s	0.4948	0.0638	**1.3×10^{-14}**
HVOT	COMLongestLatency_s	0.4751	0.0629	**6.4×10^{-14}**
HVOT	COMAverageLatency	-0.4886	0.0650	**8.1×10^{-14}**
HVOT	COMAverageLatency_s	0.4695	0.0638	**2.7×10^{-13}**
HVOT	COMLongLatencyCount	-0.4679	0.0666	**2.8×10^{-12}**
HVOT	COPInformationProcessing	0.4321	0.0662	**8.4×10^{-11}**
HVOT	COMLongLatencyCount_s	0.4191	0.0646	**1.1×10^{-10}**
HVOT	COPRelativeLongLatency	-0.4288	0.0665	**1.4×10^{-10}**
HVOT	COMNoise	-0.4159	0.0651	**2.1×10^{-10}**
HVOT	COPDrawingProcessEfficiency	0.4162	0.0654	**2.5×10^{-10}**
HVOT	COPLongLatencyCount	-0.4313	0.0679	**2.6×10^{-10}**
HVOT	COPTotalTime	-0.4245	0.0673	**3.5×10^{-10}**
HVOT	COPRelativeLongLatency_s	0.4082	0.0657	**6.4×10^{-10}**
HVOT	COPLatencyVariability	-0.4084	0.0660	**7.3×10^{-10}**
HVOT	COPInformationProcessing_s	0.4021	0.0656	**1.1×10^{-9}**
HVOT	COPDrawingProcessEfficiency_s	0.3936	0.0648	**1.5×10^{-9}**
HVOT	COPClockfaceCircularity_s	0.3854	0.0635	**1.5×10^{-9}**
HVOT	COPAverageLatency	-0.3978	0.0665	**2.6×10^{-9}**
HVOT	COPTotalTime_s	0.3891	0.0655	**3.3×10^{-9}**
HVOT	COPLatencyVariability_s	0.3872	0.0653	**3.5×10^{-9}**
HVOT	COPDrawingEfficiency	0.3766	0.0655	**1.0×10^{-8}**
HVOT	COPLongestLatency	-0.3771	0.0658	**1.1×10^{-8}**
HVOT	COPAverageLatency_s	0.3733	0.0656	**1.4×10^{-8}**
HVOT	COPClockfaceCircularity	-0.3566	0.0637	**2.5×10^{-8}**
HVOT	COPDrawingEfficiency_s	0.3580	0.0650	**4.0×10^{-8}**
HVOT	COPLongestLatency_s	0.3574	0.0650	**4.4×10^{-8}**
HVOT	COPLongLatencyCount_s	0.3522	0.0653	**7.9×10^{-8}**
HVOT	COMNoise_s	0.3322	0.0640	**2.3×10^{-7}**
HVOT	COMVerticalSpatialPlacement_s	0.3224	0.0624	**2.6×10^{-7}**
HVOT	COMSimpleMotor	0.3337	0.0651	**3.2×10^{-7}**
HVOT	COMOscillatoryMotion_s	0.3231	0.0654	**8.3×10^{-7}**
HVOT	COMStrokeCountConformity	-0.3262	0.0668	**1.1×10^{-6}**
HVOT	COMSimpleMotor_s	0.3122	0.0641	**1.2×10^{-6}**
HVOT	COMOscillatoryMotion	-0.3289	0.0676	**1.2×10^{-6}**
HVOT	COMVerticalSpatialPlacement	-0.2883	0.0626	**4.4×10^{-6}**
HVOT	COPSimpleMotor	0.2945	0.0652	**6.6×10^{-6}**
HVOT	COMDrawingSize_s	0.2635	0.0641	**4.1×10^{-5}**
HVOT	COMDrawingSize	0.2628	0.0642	**4.5×10^{-5}**
HVOT	COPSimpleMotor_s	0.2590	0.0637	**5.0×10^{-5}**
HVOT	COPOscillatoryMotion_s	0.2398	0.0640	**1.9×10^{-4}**

HVOT	COMClockfaceCircularity_s	0.2356	0.0633	**2.1×10^{-4}**
HVOT	COPOscillatoryMotion	-0.2435	0.0659	**2.3×10^{-4}**
HVOT	COPStrokeCountConformity	-0.2594	0.0704	**2.3×10^{-4}**
HVOT	COMHorizontalSpatialPlacement_s	0.2230	0.0630	**4.1×10^{-4}**
HVOT	COMPercentInkTime	0.2250	0.0641	**4.6×10^{-4}**
HVOT	COMPercentThinkTime	-0.2227	0.0642	5.3×10^{-4}
HVOT	COMClockfaceCircularity	-0.2204	0.0640	5.8×10^{-4}
HVOT	COMPercentThinkTime_s	0.2173	0.0641	7.1×10^{-4}
HVOT	COMPercentInkTime_s	-0.2150	0.0641	8.1×10^{-4}
HVOT	COMTerminationSpeed	0.2027	0.0634	1.4×10^{-3}
HVOT	COMAverageSpeed	0.1954	0.0635	2.1×10^{-3}
HVOT	COMStrokeCountConformity_s	0.1978	0.0646	2.2×10^{-3}
HVOT	COPVerticalSpatialPlacement_s	0.1926	0.0631	2.3×10^{-3}
HVOT	COMHorizontalSpatialPlacement	-0.1916	0.0629	2.3×10^{-3}
HVOT	COPNoise	-0.1959	0.0658	2.9×10^{-3}
HVOT	COMTerminationSpeed_s	0.1881	0.0633	3.0×10^{-3}
HVOT	COPVerticalSpatialPlacement	-0.1803	0.0633	4.4×10^{-3}
HVOT	COMAverageSpeed_s	0.1809	0.0635	4.5×10^{-3}
HVOT	COMInitiationSpeed	0.1787	0.0635	5.0×10^{-3}
HVOT	COPTerminationSpeed	0.1799	0.0641	5.1×10^{-3}
HVOT	COMInitiationSpeed_s	0.1656	0.0635	9.2×10^{-3}
HVOT	COPAverageSpeed	0.1661	0.0640	9.5×10^{-3}
HVOT	COPTerminationSpeed_s	0.1615	0.0639	1.1×10^{-2}
HVOT	COPDrawingSize_s	0.1590	0.0635	1.2×10^{-2}
HVOT	COPDrawingSize	0.1510	0.0637	1.8×10^{-2}
HVOT	COPAverageSpeed_s	0.1422	0.0637	2.6×10^{-2}
HVOT	COMMaxSpeed	0.1355	0.0630	3.2×10^{-2}
HVOT	COPPercentInkTime	0.1310	0.0653	4.5×10^{-2}
HVOT	COPInitiationSpeed	0.1277	0.0638	4.5×10^{-2}
HVOT	COPPercentThinkTime_s	0.1300	0.0653	4.7×10^{-2}
HVOT	COMMaxSpeed_s	0.1218	0.0630	5.3×10^{-2}
HVOT	COPPercentThinkTime	-0.1190	0.0655	6.9×10^{-2}
HVOT	COPPercentInkTime_s	-0.1183	0.0655	7.1×10^{-2}
HVOT	COPMaxSpeed	0.1148	0.0636	7.1×10^{-2}
HVOT	COPInitiationSpeed_s	0.1093	0.0636	8.6×10^{-2}
HVOT	COPNoise_s	0.1114	0.0658	9.1×10^{-2}
HVOT	COMInkLength_s	0.1049	0.0647	1.0×10^{-1}
HVOT	COMInkLength	0.1024	0.0646	1.1×10^{-1}
HVOT	COPMaxSpeed_s	0.0928	0.0634	1.4×10^{-1}
HVOT	COPStrokeCountConformity_s	0.0882	0.0651	1.8×10^{-1}
HVOT	COPInkLength_s	0.0319	0.0641	6.2×10^{-1}
HVOT	COPHorizontalSpatialPlacement_s	0.0249	0.0639	7.0×10^{-1}
HVOT	COPInkLength	0.0207	0.0646	7.5×10^{-1}

HVOT	COPHorizontalSpatialPlacement	-0.0186	0.0638	7.7×10^{-1}
BNT30	COMDrawingProcessEfficiency	1.1734	0.1505	**1.0×10^{-14}**
BNT30	DCTScore	1.2029	0.1554	**1.5×10^{-14}**
BNT30	COMDrawingEfficiency	1.1331	0.1496	**5.5×10^{-14}**
BNT30	COMLongLatencyCount	-1.1657	0.1546	**7.0×10^{-14}**
BNT30	COMDrawingProcessEfficiency_s	1.0999	0.1484	**1.8×10^{-13}**
BNT30	COMSpatialReasoning	1.1131	0.1510	**2.5×10^{-13}**
BNT30	COMSpatialReasoning_s	1.0941	0.1486	**2.6×10^{-13}**
BNT30	COMDrawingEfficiency_s	1.0884	0.1483	**3.1×10^{-13}**
BNT30	COMComponentPlacement	-1.1138	0.1529	**4.6×10^{-13}**
BNT30	COMRelativeLongLatency	-1.0831	0.1493	**5.7×10^{-13}**
BNT30	COMComponentPlacement_s	1.0647	0.1498	**1.6×10^{-12}**
BNT30	COMRelativeLongLatency_s	1.0146	0.1478	**8.7×10^{-12}**
BNT30	COMTotalTime	-1.0319	0.1514	**1.2×10^{-11}**
BNT30	COMInformationProcessing	1.0071	0.1497	**2.2×10^{-11}**
BNT30	COMAverageLatency	-1.0114	0.1513	**3.0×10^{-11}**
BNT30	COMInformationProcessing_s	0.9557	0.1479	**1.3×10^{-10}**
BNT30	COMTotalTime_s	0.9569	0.1486	**1.5×10^{-10}**
BNT30	COMLatencyVariability	-0.9588	0.1493	**1.7×10^{-10}**
BNT30	COMAverageLatency_s	0.9278	0.1488	**5.4×10^{-10}**
BNT30	COMLatencyVariability_s	0.8968	0.1476	**1.5×10^{-9}**
BNT30	COMLongestLatency	-0.8675	0.1490	**6.7×10^{-9}**
BNT30	COMLongestLatency_s	0.8056	0.1475	**5.3×10^{-8}**
BNT30	COMLongLatencyCount_s	0.7705	0.1491	**2.6×10^{-7}**
BNT30	COMHorizontalSpatialPlacement_s	0.7501	0.1466	**3.4×10^{-7}**
BNT30	COPSpatialReasoning	0.7478	0.1508	**7.7×10^{-7}**
BNT30	COPLongLatencyCount	-0.7734	0.1569	**8.9×10^{-7}**
BNT30	COPComponentPlacement_s	0.7202	0.1485	**1.3×10^{-6}**
BNT30	COPComponentPlacement	-0.7244	0.1515	**1.9×10^{-6}**
BNT30	COPSpatialReasoning_s	0.7047	0.1488	**2.3×10^{-6}**
BNT30	COMPercentThinkTime	-0.7021	0.1490	**2.6×10^{-6}**
BNT30	COMHorizontalSpatialPlacement	-0.6911	0.1467	**2.6×10^{-6}**
BNT30	COMPercentInkTime	0.6964	0.1489	**3.1×10^{-6}**
BNT30	COMPercentInkTime_s	-0.6813	0.1491	**5.2×10^{-6}**
BNT30	COMPercentThinkTime_s	0.6754	0.1490	**6.1×10^{-6}**
BNT30	COPStrokeCountConformity	-0.7176	0.1620	**1.0×10^{-5}**
BNT30	COMNoise	-0.6725	0.1534	**1.2×10^{-5}**
BNT30	COPTotalTime	-0.6256	0.1554	**5.9×10^{-5}**
BNT30	COPInformationProcessing	0.6022	0.1530	**8.6×10^{-5}**
BNT30	COPRelativeLongLatency	-0.6018	0.1536	**9.2×10^{-5}**
BNT30	COPTotalTime_s	0.5840	0.1510	**1.1×10^{-4}**
BNT30	COPRelativeLongLatency_s	0.5805	0.1519	**1.4×10^{-4}**

BNT30	COMStrokeCountConformity	-0.5812	0.1561	**2.0×10^{-4}**
BNT30	COPDrawingEfficiency	0.5633	0.1520	**2.2×10^{-4}**
BNT30	COMDrawingSize	0.5488	0.1493	**2.4×10^{-4}**
BNT30	COPAverageLatency	-0.5536	0.1535	**3.2×10^{-4}**
BNT30	COMDrawingSize_s	0.5309	0.1491	**3.8×10^{-4}**
BNT30	COPDrawingProcessEfficiency	0.5412	0.1520	**3.8×10^{-4}**
BNT30	COPAverageLatency_s	0.5221	0.1512	5.7×10^{-4}
BNT30	COPDrawingEfficiency_s	0.5149	0.1504	6.3×10^{-4}
BNT30	COPInformationProcessing_s	0.5182	0.1516	6.4×10^{-4}
BNT30	COMAverageSpeed	0.4939	0.1482	8.8×10^{-4}
BNT30	COPDrawingProcessEfficiency_s	0.4798	0.1504	1.4×10^{-3}
BNT30	COPLatencyVariability	-0.4794	0.1527	1.7×10^{-3}
BNT30	COMSimpleMotor	0.4762	0.1528	1.9×10^{-3}
BNT30	COMAverageSpeed_s	0.4582	0.1480	2.0×10^{-3}
BNT30	COMMaxSpeed	0.4528	0.1474	2.2×10^{-3}
BNT30	COPLatencyVariability_s	0.4564	0.1513	2.6×10^{-3}
BNT30	COMInitiationSpeed	0.4461	0.1480	2.6×10^{-3}
BNT30	COMTerminationSpeed	0.4327	0.1483	3.6×10^{-3}
BNT30	COPClockfaceCircularity_s	0.4310	0.1482	3.7×10^{-3}
BNT30	COMSimpleMotor_s	0.4317	0.1505	4.2×10^{-3}
BNT30	COMInitiationSpeed_s	0.4167	0.1479	4.9×10^{-3}
BNT30	COMMaxSpeed_s	0.4102	0.1473	5.4×10^{-3}
BNT30	COMTerminationSpeed_s	0.3941	0.1480	7.8×10^{-3}
BNT30	COMNoise_s	0.3866	0.1505	1.0×10^{-2}
BNT30	COPLongestLatency	-0.3805	0.1522	1.3×10^{-2}
BNT30	COPNoise	-0.3795	0.1532	1.3×10^{-2}
BNT30	COPClockfaceCircularity	-0.3597	0.1486	1.6×10^{-2}
BNT30	COPLongestLatency_s	0.3609	0.1507	1.7×10^{-2}
BNT30	COMVerticalSpatialPlacement_s	0.3370	0.1471	2.2×10^{-2}
BNT30	COPPercentInkTime	0.3281	0.1506	2.9×10^{-2}
BNT30	COPPercentThinkTime_s	0.3218	0.1508	3.3×10^{-2}
BNT30	COPInitiationSpeed	0.3055	0.1483	4.0×10^{-2}
BNT30	COPVerticalSpatialPlacement_s	0.3013	0.1480	4.2×10^{-2}
BNT30	COPVerticalSpatialPlacement	-0.2926	0.1484	4.9×10^{-2}
BNT30	COPSimpleMotor	0.3002	0.1523	4.9×10^{-2}
BNT30	COPStrokeCountConformity_s	-0.2939	0.1493	4.9×10^{-2}
BNT30	COPDrawingSize_s	0.2893	0.1482	5.1×10^{-2}
BNT30	COPLongLatencyCount_s	0.2889	0.1500	5.4×10^{-2}
BNT30	COPAverageSpeed	0.2807	0.1488	5.9×10^{-2}
BNT30	COMClockfaceCircularity_s	0.2718	0.1482	6.7×10^{-2}
BNT30	COMVerticalSpatialPlacement	-0.2677	0.1474	6.9×10^{-2}
BNT30	COPDrawingSize	0.2668	0.1483	7.2×10^{-2}
BNT30	COPPercentThinkTime	-0.2637	0.1510	8.1×10^{-2}

BNT30	COMInkLength_s	0.2621	0.1504	8.2×10^{-2}
BNT30	COPPercentInkTime_s	-0.2587	0.1512	8.7×10^{-2}
BNT30	COMInkLength	0.2506	0.1504	9.6×10^{-2}
BNT30	COPInitiationSpeed_s	0.2424	0.1479	1.0×10^{-1}
BNT30	COMOscillatoryMotion_s	0.2492	0.1538	1.1×10^{-1}
BNT30	COPSimpleMotor_s	0.2322	0.1488	1.2×10^{-1}
BNT30	COMClockfaceCircularity	-0.2304	0.1495	1.2×10^{-1}
BNT30	COPAverageSpeed_s	0.2077	0.1481	1.6×10^{-1}
BNT30	COPTerminationSpeed	0.2087	0.1492	1.6×10^{-1}
BNT30	COPMaxSpeed	0.1974	0.1480	1.8×10^{-1}
BNT30	COMOscillatoryMotion	-0.2109	0.1590	1.8×10^{-1}
BNT30	COPOscillatoryMotion_s	0.1662	0.1499	2.7×10^{-1}
BNT30	COPHorizontalSpatialPlacement_s	0.1621	0.1482	2.7×10^{-1}
BNT30	COPHorizontalSpatialPlacement	-0.1572	0.1481	2.9×10^{-1}
BNT30	COPTerminationSpeed_s	0.1514	0.1486	3.1×10^{-1}
BNT30	COPOscillatoryMotion	-0.1383	0.1544	3.7×10^{-1}
BNT30	COPMaxSpeed_s	0.1262	0.1476	3.9×10^{-1}
BNT30	COPInkLength	-0.1055	0.1499	4.8×10^{-1}
BNT30	COPInkLength_s	-0.0812	0.1490	5.9×10^{-1}
BNT30	COPNoise_s	-0.0681	0.1512	6.5×10^{-1}
BNT30	COMStrokeCountConformity_s	0.0092	0.1495	9.5×10^{-1}
FAS	COMDrawingProcessEfficiency	2.7086	0.2781	**6.3×10^{-22}**
FAS	COMDrawingEfficiency	2.6606	0.2766	**1.9×10^{-21}**
FAS	DCTScore	2.6690	0.2875	**4.2×10^{-20}**
FAS	COMDrawingEfficiency_s	2.5459	0.2744	**4.3×10^{-20}**
FAS	COMDrawingProcessEfficiency_s	2.5355	0.2745	**6.3×10^{-20}**
FAS	COMSpatialReasoning_s	2.2251	0.2769	**1.6×10^{-15}**
FAS	COMSpatialReasoning	2.2573	0.2813	**1.7×10^{-15}**
FAS	COMComponentPlacement	-2.2672	0.2855	**3.3×10^{-15}**
FAS	COMComponentPlacement_s	2.1956	0.2794	**6.3×10^{-15}**
FAS	COMAverageLatency	-2.1279	0.2823	**7.2×10^{-14}**
FAS	COMInformationProcessing	2.0975	0.2794	**8.9×10^{-14}**
FAS	COMAverageLatency_s	2.0018	0.2776	**7.8×10^{-13}**
FAS	COMInformationProcessing_s	1.9636	0.2764	**1.7×10^{-12}**
FAS	COMLatencyVariability	-1.9640	0.2785	**2.4×10^{-12}**
FAS	COMLatencyVariability_s	1.8597	0.2755	**1.9×10^{-11}**
FAS	COMLongLatencyCount	-1.9621	0.2907	**1.9×10^{-11}**
FAS	COMRelativeLongLatency	-1.8801	0.2800	**2.5×10^{-11}**
FAS	COMLongestLatency	-1.8594	0.2779	**2.9×10^{-11}**
FAS	COMLongLatencyCount_s	1.8570	0.2796	**4.0×10^{-11}**
FAS	COMTotalTime	-1.8491	0.2840	**9.4×10^{-11}**
FAS	COMLongestLatency_s	1.7625	0.2752	**1.9×10^{-10}**

FAS	COMRelativeLongLatency_s	1.7585	0.2773	**2.8×10^{-10}**
FAS	COMTotalTime_s	1.7232	0.2789	**7.8×10^{-10}**
FAS	COPTotalTime	-1.6110	0.2886	**2.7×10^{-8}**
FAS	COPClockfaceCircularity_s	1.5333	0.2754	**2.9×10^{-8}**
FAS	COPAverageLatency	-1.5673	0.2849	**4.3×10^{-8}**
FAS	COMDrawingSize	1.4952	0.2780	**8.4×10^{-8}**
FAS	COPDrawingProcessEfficiency	1.4985	0.2828	**1.3×10^{-7}**
FAS	COPDrawingEfficiency	1.4890	0.2823	**1.5×10^{-7}**
FAS	COPInformationProcessing	1.4962	0.2847	**1.6×10^{-7}**
FAS	COPLongLatencyCount	-1.5281	0.2923	**1.9×10^{-7}**
FAS	COMDrawingSize_s	1.4469	0.2775	**2.0×10^{-7}**
FAS	COMSimpleMotor	1.4405	0.2841	**4.3×10^{-7}**
FAS	COPRelativeLongLatency	-1.4164	0.2858	**7.8×10^{-7}**
FAS	COPClockfaceCircularity	-1.3559	0.2763	**1.0×10^{-6}**
FAS	COPDrawingEfficiency_s	1.3728	0.2799	**1.0×10^{-6}**
FAS	COPTotalTime_s	1.3713	0.2815	**1.2×10^{-6}**
FAS	COPAverageLatency_s	1.3624	0.2814	**1.4×10^{-6}**
FAS	COPDrawingProcessEfficiency_s	1.3262	0.2804	**2.4×10^{-6}**
FAS	COMSimpleMotor_s	1.2993	0.2797	**3.6×10^{-6}**
FAS	COPInformationProcessing_s	1.2588	0.2825	**8.8×10^{-6}**
FAS	COPRelativeLongLatency_s	1.2388	0.2832	**1.3×10^{-5}**
FAS	COMInkLength	1.2125	0.2798	**1.5×10^{-5}**
FAS	COMAverageSpeed	1.1874	0.2756	**1.7×10^{-5}**
FAS	COMInkLength_s	1.2043	0.2800	**1.8×10^{-5}**
FAS	COMInitiationSpeed	1.1771	0.2752	**2.0×10^{-5}**
FAS	COPLatencyVariability	-1.2065	0.2846	**2.3×10^{-5}**
FAS	COMMaxSpeed	1.1492	0.2741	**2.9×10^{-5}**
FAS	COMPercentInkTime	1.1611	0.2784	**3.2×10^{-5}**
FAS	COMPercentThinkTime	-1.1295	0.2786	**5.2×10^{-5}**
FAS	COPSimpleMotor	1.1285	0.2830	**6.9×10^{-5}**
FAS	COMInitiationSpeed_s	1.0769	0.2753	**9.5×10^{-5}**
FAS	COMPercentThinkTime_s	1.0819	0.2787	**1.1×10^{-4}**
FAS	COMClockfaceCircularity	-1.0786	0.2784	**1.1×10^{-4}**
FAS	COMAverageSpeed_s	1.0660	0.2754	**1.1×10^{-4}**
FAS	COPLongestLatency	-1.0948	0.2838	**1.2×10^{-4}**
FAS	COPSpatialReasoning	1.0835	0.2825	**1.3×10^{-4}**
FAS	COMClockfaceCircularity_s	1.0542	0.2761	**1.4×10^{-4}**
FAS	COMMaxSpeed_s	1.0335	0.2741	**1.7×10^{-4}**
FAS	COMPercentInkTime_s	-1.0497	0.2790	**1.7×10^{-4}**
FAS	COMOscillatoryMotion	-1.1011	0.2965	**2.1×10^{-4}**
FAS	COPComponentPlacement_s	1.0269	0.2776	**2.2×10^{-4}**
FAS	COMTerminationSpeed	1.0185	0.2762	**2.3×10^{-4}**
FAS	COPLatencyVariability_s	1.0367	0.2822	**2.4×10^{-4}**

FAS	COMOscillatoryMotion_s	1.0431	0.2866	**2.8×10^{-4}**
FAS	COPSpatialReasoning_s	0.9995	0.2786	**3.4×10^{-4}**
FAS	COPLongLatencyCount_s	0.9795	0.2803	4.9×10^{-4}
FAS	COMNoise	-1.0005	0.2876	5.2×10^{-4}
FAS	COPSimpleMotor_s	0.9519	0.2768	6.0×10^{-4}
FAS	COPDrawingSize_s	0.9452	0.2758	6.2×10^{-4}
FAS	COPLongestLatency_s	0.9450	0.2811	7.9×10^{-4}
FAS	COPComponentPlacement	-0.9522	0.2835	8.0×10^{-4}
FAS	COMTerminationSpeed_s	0.8944	0.2758	1.2×10^{-3}
FAS	COPDrawingSize	0.8856	0.2761	1.4×10^{-3}
FAS	COPOscillatoryMotion_s	0.8696	0.2789	1.8×10^{-3}
FAS	COPOscillatoryMotion	-0.8382	0.2873	3.6×10^{-3}
FAS	COPTerminationSpeed	0.7934	0.2780	4.4×10^{-3}
FAS	COPAverageSpeed	0.7822	0.2772	4.8×10^{-3}
FAS	COPPercentInkTime	0.7320	0.2811	9.3×10^{-3}
FAS	COPMaxSpeed	0.7136	0.2757	9.7×10^{-3}
FAS	COPPercentThinkTime_s	0.7002	0.2814	1.3×10^{-2}
FAS	COMNoise_s	0.6915	0.2826	1.4×10^{-2}
FAS	COMVerticalSpatialPlacement_s	0.6710	0.2746	1.5×10^{-2}
FAS	COPInitiationSpeed	0.6742	0.2762	1.5×10^{-2}
FAS	COPPercentThinkTime	-0.6708	0.2820	1.7×10^{-2}
FAS	COPTerminationSpeed_s	0.6466	0.2773	2.0×10^{-2}
FAS	COPPercentInkTime_s	-0.6423	0.2822	2.3×10^{-2}
FAS	COPNoise	-0.6408	0.2866	2.5×10^{-2}
FAS	COMVerticalSpatialPlacement	-0.6119	0.2756	2.6×10^{-2}
FAS	COPAverageSpeed_s	0.5932	0.2764	3.2×10^{-2}
FAS	COPMaxSpeed_s	0.5428	0.2752	4.9×10^{-2}
FAS	COPInitiationSpeed_s	0.5162	0.2756	6.1×10^{-2}
FAS	COPHorizontalSpatialPlacement_s	-0.5135	0.2761	6.3×10^{-2}
FAS	COPHorizontalSpatialPlacement	0.5098	0.2758	6.5×10^{-2}
FAS	COPStrokeCountConformity	-0.5017	0.3047	1.0×10^{-1}
FAS	COPStrokeCountConformity_s	-0.4499	0.2811	1.1×10^{-1}
FAS	COPNoise_s	0.3962	0.2839	1.6×10^{-1}
FAS	COMHorizontalSpatialPlacement_s	0.3498	0.2758	2.0×10^{-1}
FAS	COMHorizontalSpatialPlacement	-0.3398	0.2752	2.2×10^{-1}
FAS	COMStrokeCountConformity	-0.3439	0.2929	2.4×10^{-1}
FAS	COPVerticalSpatialPlacement_s	0.2418	0.2767	3.8×10^{-1}
FAS	COPVerticalSpatialPlacement	-0.2313	0.2768	4.0×10^{-1}
FAS	COPInkLength_s	0.1254	0.2774	6.5×10^{-1}
FAS	COMStrokeCountConformity_s	-0.1047	0.2808	7.1×10^{-1}
FAS	COPInkLength	0.0983	0.2793	7.2×10^{-1}
FAS-animal	COMInformationProcessing	1.0223	0.1324	**1.8×10^{-14}**

FAS-animal	COMSpatialReasoning_s	1.0168	0.1317	**1.8×10^{-14}**
FAS-animal	COMComponentPlacement_s	1.0209	0.1328	**2.3×10^{-14}**
FAS-animal	COMSpatialReasoning	1.0221	0.1340	**3.6×10^{-14}**
FAS-animal	COMComponentPlacement	-1.0299	0.1356	**4.6×10^{-14}**
FAS-animal	COMAverageLatency	-1.0028	0.1340	**1.1×10^{-13}**
FAS-animal	COMInformationProcessing_s	0.9784	0.1309	**1.1×10^{-13}**
FAS-animal	DCTScore	1.0181	0.1382	**2.5×10^{-13}**
FAS-animal	COMDrawingProcessEfficiency	0.9846	0.1339	**2.7×10^{-13}**
FAS-animal	COMRelativeLongLatency	-0.9721	0.1325	**3.1×10^{-13}**
FAS-animal	COMLatencyVariability	-0.9694	0.1321	**3.2×10^{-13}**
FAS-animal	COMAverageLatency_s	0.9621	0.1316	**3.8×10^{-13}**
FAS-animal	COMLongLatencyCount	-0.9939	0.1374	**6.6×10^{-13}**
FAS-animal	COMLatencyVariability_s	0.9279	0.1306	**1.7×10^{-12}**
FAS-animal	COMRelativeLongLatency_s	0.9227	0.1311	**2.7×10^{-12}**
FAS-animal	COMDrawingProcessEfficiency_s	0.9182	0.1320	**4.7×10^{-12}**
FAS-animal	COMLongestLatency	-0.9115	0.1318	**6.3×10^{-12}**
FAS-animal	COMDrawingEfficiency	0.9193	0.1331	**6.7×10^{-12}**
FAS-animal	COMDrawingEfficiency_s	0.8812	0.1320	**3.1×10^{-11}**
FAS-animal	COMLongestLatency_s	0.8683	0.1305	**3.7×10^{-11}**
FAS-animal	COMTotalTime	-0.8892	0.1345	**4.8×10^{-11}**
FAS-animal	COMLongLatencyCount_s	0.8697	0.1318	**5.3×10^{-11}**
FAS-animal	COMTotalTime_s	0.8300	0.1320	**3.9×10^{-10}**
FAS-animal	COMPercentInkTime	0.7341	0.1319	**3.0×10^{-8}**
FAS-animal	COMPercentThinkTime	-0.7310	0.1320	**3.5×10^{-8}**
FAS-animal	COMPercentThinkTime_s	0.7139	0.1320	**7.1×10^{-8}**
FAS-animal	COMPercentInkTime_s	-0.7118	0.1321	**7.9×10^{-8}**
FAS-animal	COPLongLatencyCount	-0.5546	0.1396	**7.3×10^{-5}**
FAS-animal	COPInformationProcessing	0.5285	0.1359	**1.0×10^{-4}**
FAS-animal	COPAverageLatency	-0.5094	0.1362	**1.9×10^{-4}**
FAS-animal	COPRelativeLongLatency	-0.5042	0.1364	**2.3×10^{-4}**
FAS-animal	COPDrawingEfficiency	0.4719	0.1350	4.8×10^{-4}
FAS-animal	COPTotalTime	-0.4820	0.1381	4.9×10^{-4}
FAS-animal	COPDrawingProcessEfficiency	0.4623	0.1350	6.3×10^{-4}
FAS-animal	COPRelativeLongLatency_s	0.4587	0.1350	6.9×10^{-4}
FAS-animal	COPAverageLatency_s	0.4510	0.1343	8.0×10^{-4}
FAS-animal	COPInformationProcessing_s	0.4446	0.1347	9.8×10^{-4}
FAS-animal	COPOscillatoryMotion_s	0.4202	0.1328	1.6×10^{-3}
FAS-animal	COPDrawingEfficiency_s	0.4194	0.1336	1.7×10^{-3}
FAS-animal	COPLatencyVariability	-0.4241	0.1356	1.8×10^{-3}
FAS-animal	COMNoise	-0.4267	0.1365	1.8×10^{-3}
FAS-animal	COPTotalTime_s	0.4098	0.1343	2.3×10^{-3}
FAS-animal	COPOscillatoryMotion	-0.4086	0.1368	2.9×10^{-3}
FAS-animal	COPDrawingProcessEfficiency_s	0.3970	0.1336	3.0×10^{-3}

FAS-animal	COPClockfaceCircularity_s	0.3899	0.1316	3.1×10^{-3}
FAS-animal	COMVerticalSpatialPlacement_s	0.3723	0.1305	4.4×10^{-3}
FAS-animal	COMInitiationSpeed	0.3735	0.1314	4.5×10^{-3}
FAS-animal	COPSimpleMotor	0.3823	0.1351	4.7×10^{-3}
FAS-animal	COMClockfaceCircularity_s	0.3682	0.1315	5.1×10^{-3}
FAS-animal	COPLatencyVariability_s	0.3751	0.1344	5.3×10^{-3}
FAS-animal	COPSpatialReasoning	0.3637	0.1345	6.9×10^{-3}
FAS-animal	COPLongestLatency	-0.3618	0.1351	7.5×10^{-3}
FAS-animal	COMClockfaceCircularity	-0.3490	0.1326	8.5×10^{-3}
FAS-animal	COMInitiationSpeed_s	0.3426	0.1314	9.2×10^{-3}
FAS-animal	COMSimpleMotor	0.3473	0.1358	1.1×10^{-2}
FAS-animal	COPComponentPlacement_s	0.3351	0.1324	1.1×10^{-2}
FAS-animal	COMNoise_s	0.3354	0.1336	1.2×10^{-2}
FAS-animal	COMOscillatoryMotion	-0.3466	0.1410	1.4×10^{-2}
FAS-animal	COMOscillatoryMotion_s	0.3335	0.1365	1.5×10^{-2}
FAS-animal	COPSpatialReasoning_s	0.3238	0.1326	1.5×10^{-2}
FAS-animal	COPComponentPlacement	-0.3271	0.1351	1.6×10^{-2}
FAS-animal	COPLongLatencyCount_s	0.3219	0.1332	1.6×10^{-2}
FAS-animal	COPLongestLatency_s	0.3218	0.1338	1.6×10^{-2}
FAS-animal	COMVerticalSpatialPlacement	-0.3131	0.1308	1.7×10^{-2}
FAS-animal	COPPercentInkTime	0.3148	0.1337	1.9×10^{-2}
FAS-animal	COPClockfaceCircularity	-0.3054	0.1319	2.1×10^{-2}
FAS-animal	COPSimpleMotor_s	0.3037	0.1320	2.2×10^{-2}
FAS-animal	COPPercentThinkTime_s	0.3076	0.1339	2.2×10^{-2}
FAS-animal	COMDrawingSize	0.2960	0.1329	2.6×10^{-2}
FAS-animal	COPDrawingSize_s	0.2885	0.1315	2.8×10^{-2}
FAS-animal	COMSimpleMotor_s	0.2877	0.1338	3.2×10^{-2}
FAS-animal	COMDrawingSize_s	0.2822	0.1326	3.3×10^{-2}
FAS-animal	COPPercentThinkTime	-0.2752	0.1341	4.0×10^{-2}
FAS-animal	COPDrawingSize	0.2689	0.1316	4.1×10^{-2}
FAS-animal	COPPercentInkTime_s	-0.2689	0.1342	4.5×10^{-2}
FAS-animal	COMAverageSpeed	0.2485	0.1318	6.0×10^{-2}
FAS-animal	COMMaxSpeed	0.2409	0.1311	6.6×10^{-2}
FAS-animal	COMStrokeCountConformity	-0.2338	0.1390	9.3×10^{-2}
FAS-animal	COMAverageSpeed_s	0.2065	0.1317	1.2×10^{-1}
FAS-animal	COMMaxSpeed_s	0.1990	0.1310	1.3×10^{-1}
FAS-animal	COPStrokeCountConformity	-0.2119	0.1445	1.4×10^{-1}
FAS-animal	COMHorizontalSpatialPlacement_s	0.1678	0.1309	2.0×10^{-1}
FAS-animal	COPInitiationSpeed	0.1689	0.1318	2.0×10^{-1}
FAS-animal	COPMaxSpeed	0.1640	0.1314	2.1×10^{-1}
FAS-animal	COMInkLength_s	0.1662	0.1336	2.1×10^{-1}
FAS-animal	COMInkLength	0.1558	0.1336	2.4×10^{-1}
FAS-animal	COMHorizontalSpatialPlacement	-0.1513	0.1309	2.5×10^{-1}

FAS-animal	COPAverageSpeed	0.1525	0.1322	2.5×10^{-1}
FAS-animal	COMTerminationSpeed	0.1384	0.1319	2.9×10^{-1}
FAS-animal	COPVerticalSpatialPlacement_s	0.1354	0.1315	3.0×10^{-1}
FAS-animal	COPNoise	-0.1365	0.1361	3.2×10^{-1}
FAS-animal	COPTerminationSpeed	0.1255	0.1325	3.4×10^{-1}
FAS-animal	COPVerticalSpatialPlacement	-0.1238	0.1318	3.5×10^{-1}
FAS-animal	COPInitiationSpeed_s	0.1232	0.1313	3.5×10^{-1}
FAS-animal	COPStrokeCountConformity_s	-0.1083	0.1326	4.1×10^{-1}
FAS-animal	COPMaxSpeed_s	0.1038	0.1311	4.3×10^{-1}
FAS-animal	COMTerminationSpeed_s	0.0938	0.1316	4.8×10^{-1}
FAS-animal	COPAverageSpeed_s	0.0896	0.1316	5.0×10^{-1}
FAS-animal	COPHorizontalSpatialPlacement_s	0.0746	0.1316	5.7×10^{-1}
FAS-animal	COPTerminationSpeed_s	0.0720	0.1320	5.9×10^{-1}
FAS-animal	COPHorizontalSpatialPlacement	-0.0527	0.1315	6.9×10^{-1}
FAS-animal	COPInkLength_s	0.0476	0.1323	7.2×10^{-1}
FAS-animal	COPInkLength	0.0365	0.1331	7.8×10^{-1}
FAS-animal	COPNoise_s	0.0240	0.1342	8.6×10^{-1}
FAS-animal	COMStrokeCountConformity_s	-0.0059	0.1327	9.6×10^{-1}

The association of each dCDT feature with each NP test was tested by linear regression models adjusted for age and sex.

[a] Bonferroni correction was used to adjusted for multiple testing, and significant dCDT features for each NP test were claimed if *p-value* < 0.05/105 (4.76×10^{-4}), where 105 was the number of tests performed. Significant *p-value* were indicated in bold.

Supplemental Table 2 Cognitive domains and the associated dCDT features

Major domain	NP tests	Number of significant features	Significant dCDT features
Verbal memory	LMi<br>LMr<br>LMd<br>PASi<br>PASd<br>PASr	62	COMAverageLatency<br>COMAverageLatency_s<br>COMClockfaceCircularity<br>COMClockfaceCircularity_s<br>COMComponentPlacement<br>COMComponentPlacement_s<br>COMDrawingEfficiency<br>COMDrawingEfficiency_s<br>COMDrawingProcessEfficiency<br>COMDrawingProcessEfficiency_s<br>COMDrawingSize<br>COMDrawingSize_s<br>COMInformationProcessing<br>COMInformationProcessing_s<br>COMLatencyVariability<br>COMLatencyVariability_s<br>COMLongLatencyCount<br>COMLongLatencyCount_s<br>COMLongestLatency<br>COMLongestLatency_s<br>COMOscillatoryMotion_s<br>COMPercentInkTime<br>COMPercentInkTime_s<br>COMPercentThinkTime<br>COMPercentThinkTime_s<br>COMRelativeLongLatency<br>COMRelativeLongLatency_s<br>COMSpatialReasoning<br>COMSpatialReasoning_s<br>COMTotalTime<br>COMTotalTime_s<br>COPAverageLatency<br>COPAverageLatency_s<br>COPClockfaceCircularity<br>COPClockfaceCircularity_s<br>COPComponentPlacement<br>COPComponentPlacement_s<br>COPDrawingEfficiency<br>COPDrawingEfficiency_s<br>COPDrawingProcessEfficiency<br>COPDrawingProcessEfficiency_s<br>COPDrawingSize

			COPDrawingSize_s<br>COPInformationProcessing<br>COPInformationProcessing_s<br>COPLatencyVariability<br>COPLatencyVariability_s<br>COPLongLatencyCount<br>COPLongLatencyCount_s<br>COPLongestLatency<br>COPLongestLatency_s<br>COPOscillatoryMotion<br>COPOscillatoryMotion_s<br>COPRelativeLongLatency<br>COPRelativeLongLatency_s<br>COPSimpleMotor<br>COPSimpleMotor_s<br>COPSpatialReasoning<br>COPSpatialReasoning_s<br>COPTotalTime<br>COPTotalTime_s<br>DCTScore
Visual memory	VRi<br>VRd<br>VRr	71	COMAverageLatency<br>COMAverageLatency_s<br>COMAverageSpeed<br>COMAverageSpeed_s<br>COMClockfaceCircularity<br>COMClockfaceCircularity_s<br>COMComponentPlacement<br>COMComponentPlacement_s<br>COMDrawingEfficiency<br>COMDrawingEfficiency_s<br>COMDrawingProcessEfficiency<br>COMDrawingProcessEfficiency_s<br>COMDrawingSize<br>COMDrawingSize_s<br>COMInformationProcessing<br>COMInformationProcessing_s<br>COMInitiationSpeed<br>COMInitiationSpeed_s<br>COMLatencyVariability<br>COMLatencyVariability_s<br>COMLongLatencyCount<br>COMLongLatencyCount_s<br>COMLongestLatency<br>COMLongestLatency_s<br>COMNoise<br>COMNoise_s

			COMOscillatoryMotion<br>COMOscillatoryMotion_s<br>COMRelativeLongLatency<br>COMRelativeLongLatency_s<br>COMSimpleMotor<br>COMSimpleMotor_s<br>COMSpatialReasoning<br>COMSpatialReasoning_s<br>COMTerminationSpeed<br>COMTotalTime<br>COMTotalTime_s<br>COMVerticalSpatialPlacement<br>COMVerticalSpatialPlacement_s<br>COPAverageLatency<br>COPAverageLatency_s<br>COPClockfaceCircularity<br>COPClockfaceCircularity_s<br>COPComponentPlacement<br>COPComponentPlacement_s<br>COPDrawingEfficiency<br>COPDrawingEfficiency_s<br>COPDrawingProcessEfficiency<br>COPDrawingProcessEfficiency_s<br>COPDrawingSize<br>COPDrawingSize_s<br>COPInformationProcessing<br>COPInformationProcessing_s<br>COPLatencyVariability<br>COPLatencyVariability_s<br>COPLongLatencyCount<br>COPLongLatencyCount_s<br>COPLongestLatency<br>COPLongestLatency_s<br>COPOscillatoryMotion<br>COPOscillatoryMotion_s<br>COPRelativeLongLatency<br>COPRelativeLongLatency_s<br>COPSimpleMotor<br>COPSimpleMotor_s<br>COPSpatialReasoning<br>COPSpatialReasoning_s<br>COPTotalTime<br>COPTotalTime_s<br>COPVerticalSpatialPlacement_s<br>DCTScore
Attention	DSf	87	COMAverageLatency

	Trails A		COMAverageLatency_s<br>COMAverageSpeed<br>COMAverageSpeed_s<br>COMClockfaceCircularity<br>COMComponentPlacement<br>COMComponentPlacement_s<br>COMDrawingEfficiency<br>COMDrawingEfficiency_s<br>COMDrawingProcessEfficiency<br>COMDrawingProcessEfficiency_s<br>COMDrawingSize<br>COMDrawingSize_s<br>COMInformationProcessing<br>COMInformationProcessing_s<br>COMInitiationSpeed<br>COMInitiationSpeed_s<br>COMLatencyVariability<br>COMLatencyVariability_s<br>COMLongLatencyCount<br>COMLongLatencyCount_s<br>COMLongestLatency<br>COMLongestLatency_s<br>COMMaxSpeed<br>COMMaxSpeed_s<br>COMNoise<br>COMNoise_s<br>COMOscillatoryMotion<br>COMOscillatoryMotion_s<br>COMPercentInkTime<br>COMPercentInkTime_s<br>COMPercentThinkTime<br>COMPercentThinkTime_s<br>COMRelativeLongLatency<br>COMRelativeLongLatency_s<br>COMSimpleMotor<br>COMSimpleMotor_s<br>COMSpatialReasoning<br>COMSpatialReasoning_s<br>COMTerminationSpeed<br>COMTerminationSpeed_s<br>COMTotalTime<br>COMTotalTime_s<br>COPAverageLatency<br>COPAverageLatency_s<br>COPAverageSpeed<br>COPAverageSpeed_s

			COPClockfaceCircularity<br>COPClockfaceCircularity_s<br>COPComponentPlacement<br>COPComponentPlacement_s<br>COPDrawingEfficiency<br>COPDrawingEfficiency_s<br>COPDrawingProcessEfficiency<br>COPDrawingProcessEfficiency_s<br>COPInformationProcessing<br>COPInformationProcessing_s<br>COPInitiationSpeed<br>COPInitiationSpeed_s<br>COPLatencyVariability<br>COPLatencyVariability_s<br>COPLongLatencyCount<br>COPLongLatencyCount_s<br>COPLongestLatency<br>COPLongestLatency_s<br>COPMaxSpeed<br>COPMaxSpeed_s<br>COPNoise<br>COPNoise_s<br>COPOscillatoryMotion<br>COPOscillatoryMotion_s<br>COPPercentInkTime<br>COPPercentInkTime_s<br>COPPercentThinkTime<br>COPPercentThinkTime_s<br>COPRelativeLongLatency<br>COPRelativeLongLatency_s<br>COPSimpleMotor<br>COPSimpleMotor_s<br>COPSpatialReasoning<br>COPSpatialReasoning_s<br>COPStrokeCountConformity<br>COPTerminationSpeed<br>COPTerminationSpeed_s<br>COPTotalTime<br>COPTotalTime_s<br>DCTScore
Executive function	DSb<br>Trails B	89	COMAverageLatency<br>COMAverageLatency_s<br>COMAverageSpeed<br>COMAverageSpeed_s<br>COMClockfaceCircularity<br>COMClockfaceCircularity_s

			COMComponentPlacement<br>COMComponentPlacement_s<br>COMDrawingEfficiency<br>COMDrawingEfficiency_s<br>COMDrawingProcessEfficiency<br>COMDrawingProcessEfficiency_s<br>COMDrawingSize<br>COMDrawingSize_s<br>COMInformationProcessing<br>COMInformationProcessing_s<br>COMInitiationSpeed<br>COMInitiationSpeed_s<br>COMLatencyVariability<br>COMLatencyVariability_s<br>COMLongLatencyCount<br>COMLongLatencyCount_s<br>COMLongestLatency<br>COMLongestLatency_s<br>COMMaxSpeed<br>COMMaxSpeed_s<br>COMNoise<br>COMNoise_s<br>COMOscillatoryMotion<br>COMOscillatoryMotion_s<br>COMPercentInkTime<br>COMPercentInkTime_s<br>COMPercentThinkTime<br>COMPercentThinkTime_s<br>COMRelativeLongLatency<br>COMRelativeLongLatency_s<br>COMSimpleMotor<br>COMSimpleMotor_s<br>COMSpatialReasoning<br>COMSpatialReasoning_s<br>COMTerminationSpeed<br>COMTerminationSpeed_s<br>COMTotalTime<br>COMTotalTime_s<br>COPAverageLatency<br>COPAverageLatency_s<br>COPAverageSpeed<br>COPAverageSpeed_s<br>COPClockfaceCircularity<br>COPClockfaceCircularity_s<br>COPComponentPlacement<br>COPComponentPlacement_s

			COPDrawingEfficiency<br>COPDrawingEfficiency_s<br>COPDrawingProcessEfficiency<br>COPDrawingProcessEfficiency_s<br>COPDrawingSize<br>COPDrawingSize_s<br>COPInformationProcessing<br>COPInformationProcessing_s<br>COPInitiationSpeed<br>COPInitiationSpeed_s<br>COPLatencyVariability<br>COPLatencyVariability_s<br>COPLongLatencyCount<br>COPLongLatencyCount_s<br>COPLongestLatency<br>COPLongestLatency_s<br>COPMaxSpeed<br>COPMaxSpeed_s<br>COPOscillatoryMotion<br>COPOscillatoryMotion_s<br>COPPercentInkTime<br>COPPercentInkTime_s<br>COPPercentThinkTime<br>COPPercentThinkTime_s<br>COPRelativeLongLatency<br>COPRelativeLongLatency_s<br>COPSimpleMotor<br>COPSimpleMotor_s<br>COPSpatialReasoning<br>COPSpatialReasoning_s<br>COPTerminationSpeed<br>COPTerminationSpeed_s<br>COPTotalTime<br>COPTotalTime_s<br>COPVerticalSpatialPlacement<br>COPVerticalSpatialPlacement_s<br>DCTScore
Abstract reasoning	SIM	46	COMSpatialReasoning_s<br>COMSpatialReasoning<br>DCTScore<br>COMComponentPlacement_s<br>COMComponentPlacement<br>COMDrawingProcessEfficiency<br>COMDrawingEfficiency<br>COMDrawingEfficiency_s<br>COMDrawingProcessEfficiency_s

			COPSpatialReasoning<br>COMLatencyVariability<br>COPSpatialReasoning_s<br>COMInformationProcessing<br>COMLongestLatency<br>COMRelativeLongLatency<br>COMLatencyVariability_s<br>COPComponentPlacement_s<br>COMInformationProcessing_s<br>COPClockfaceCircularity_s<br>COMAverageLatency<br>COMLongestLatency_s<br>COMDrawingSize<br>COPComponentPlacement<br>COMDrawingSize_s<br>COMLongLatencyCount_s<br>COMRelativeLongLatency_s<br>COMLongLatencyCount<br>COMAverageLatency_s<br>COMOscillatoryMotion_s<br>COPClockfaceCircularity<br>COMSimpleMotor<br>COMOscillatoryMotion<br>COMTotalTime<br>COMClockfaceCircularity_s<br>COMClockfaceCircularity<br>COMInkLength_s<br>COMInkLength<br>COMSimpleMotor_s<br>COMTotalTime_s<br>COMVerticalSpatialPlacement_s<br>COMInitiationSpeed<br>COMHorizontalSpatialPlacement_s<br>COMPercentInkTime<br>COPDrawingEfficiency<br>COMPercentThinkTime<br>COPOscillatoryMotion_s
Visuoperceptual organization	HVOT	66	DCTScore<br>COMComponentPlacement_s<br>COMComponentPlacement<br>COMSpatialReasoning_s<br>COMSpatialReasoning<br>COPComponentPlacement<br>COPComponentPlacement_s<br>COPSpatialReasoning<br>COMDrawingProcessEfficiency

			COMDrawingEfficiency<br>COPSpatialReasoning_s<br>COMDrawingEfficiency_s<br>COMDrawingProcessEfficiency_s<br>COMRelativeLongLatency<br>COMLatencyVariability<br>COMInformationProcessing<br>COMTotalTime<br>COMRelativeLongLatency_s<br>COMLatencyVariability_s<br>COMInformationProcessing_s<br>COMLongestLatency<br>COMTotalTime_s<br>COMLongestLatency_s<br>COMAverageLatency<br>COMAverageLatency_s<br>COMLongLatencyCount<br>COPInformationProcessing<br>COMLongLatencyCount_s<br>COPRelativeLongLatency<br>COMNoise<br>COPDrawingProcessEfficiency<br>COPLongLatencyCount<br>COPTotalTime<br>COPRelativeLongLatency_s<br>COPLatencyVariability<br>COPInformationProcessing_s<br>COPDrawingProcessEfficiency_s<br>COPClockfaceCircularity_s<br>COPAverageLatency<br>COPTotalTime_s<br>COPLatencyVariability_s<br>COPDrawingEfficiency<br>COPLongestLatency<br>COPAverageLatency_s<br>COPClockfaceCircularity<br>COPDrawingEfficiency_s<br>COPLongestLatency_s<br>COPLongLatencyCount_s<br>COMNoise_s<br>COMVerticalSpatialPlacement_s<br>COMSimpleMotor<br>COMOscillatoryMotion_s<br>COMStrokeCountConformity<br>COMSimpleMotor_s<br>COMOscillatoryMotion

			COMVerticalSpatialPlacement<br>COPSimpleMotor<br>COMDrawingSize_s<br>COMDrawingSize<br>COPSimpleMotor_s<br>COPOscillatoryMotion_s<br>COMClockfaceCircularity_s<br>COPOscillatoryMotion<br>COPStrokeCountConformity<br>COMHorizontalSpatialPlacement_s<br>COMPercentInkTime
Language	BNT30	47	COMDrawingProcessEfficiency<br>DCTScore<br>COMDrawingEfficiency<br>COMLongLatencyCount<br>COMDrawingProcessEfficiency_s<br>COMSpatialReasoning<br>COMSpatialReasoning_s<br>COMDrawingEfficiency_s<br>COMComponentPlacement<br>COMRelativeLongLatency<br>COMComponentPlacement_s<br>COMRelativeLongLatency_s<br>COMTotalTime<br>COMInformationProcessing<br>COMAverageLatency<br>COMInformationProcessing_s<br>COMTotalTime_s<br>COMLatencyVariability<br>COMAverageLatency_s<br>COMLatencyVariability_s<br>COMLongestLatency<br>COMLongestLatency_s<br>COMLongLatencyCount_s<br>COMHorizontalSpatialPlacement_s<br>COPSpatialReasoning<br>COPLongLatencyCount<br>COPComponentPlacement_s<br>COPComponentPlacement<br>COPSpatialReasoning_s<br>COMPercentThinkTime<br>COMHorizontalSpatialPlacement<br>COMPercentInkTime<br>COMPercentInkTime_s<br>COMPercentThinkTime_s<br>COPStrokeCountConformity

			COMNoise<br>COPTotalTime<br>COPInformationProcessing<br>COPRelativeLongLatency<br>COPTotalTime_s<br>COPRelativeLongLatency_s<br>COMStrokeCountConformity<br>COPDrawingEfficiency<br>COMDrawingSize<br>COPAverageLatency<br>COMDrawingSize_s<br>COPDrawingProcessEfficiency
